# Frontiers of protected areas versus forest exploitation: Assessing habitat network functionality in 16 case study regions globally

**DOI:** 10.1007/s13280-021-01628-5

**Published:** 2021-10-17

**Authors:** Per Angelstam, Andra-Cosmina Albulescu, Ollier Duranton F. Andrianambinina, Réka Aszalós, Eugene Borovichev, Walter Cano Cardona, Denis Dobrynin, Mariia Fedoriak, Dejan Firm, Malcolm L. Hunter, Wil de Jong, David Lindenmayer, Michael Manton, Juan J. Monge, Pavel Mezei, Galina Michailova, Carlos L. Muñoz Brenes, Guillermo Martínez Pastur, Olga V. Petrova, Victor Petrov, Benny Pokorny, Serge C. Rafanoharana, Yamina Micaela Rosas, Bob Robert Seymour, Patrick O. Waeber, Lucienne Wilmé, Taras Yamelynets, Tzvetan Zlatanov

**Affiliations:** 1 School for Forest Management, Faculty of Forest Sciences, Swedish University of Agricultural Sciences, PO Box 43, 73921 Skinnskatteberg, Sweden; 2 Department of Forestry and Wildlife Management, Inland Norway University of Applied Sciences, Campus Evenstad, 2480 Koppang, Norway; 3 Department of Geography, Faculty of Geography and Geology, “Alexandru Ioan Cuza” University of Iaşi, Carol I Boulevard No. 11, 700506 Iasi, Romania; 4 Madagascar National Parks, BP 1424, Ambatobe, Lot AI 10 C Mandrosoa, 103 Antananarivo, Madagascar; 5 Centre for Ecological Research, Institute of Ecology and Botany, Alkotmány u. 2-4, Vácrátót, 2163 Hungary; 6 Institute of the Industrial Ecology Problems of the North of the Kola Science Center of RAS, Akademgorodok Street 14a, Apatity, Murmansk, Russia; 7 International Union for Conservation of Nature-Project Integration of Protected Areas from Amazon Biome, República del Salvador Av. N 34-127 and Suiza, PO Box 170515, Quito, Ecuador; 8 Department of Geographical and Historical Studies, University of Eastern Finland, P.O. Box 111, 80101 Joensuu, Finland; 9 Department of Ecology and Biomonitoring, Institute of Biology, Chemistry and Bioresources, Yuriy Fedkovych Chernivtsi National University, 2 Kotsyubynskyi Street, Chernivtsi, 58012 Ukraine; 10 New Zealand Forest Research Institute-Scion, 49 Sala Street, Rotorua, 3010 New Zealand; 11 Department of Wildlife, Fisheries, and Conservation Biology, University of Maine, 5755 Nutting Hall, Room 226, Orono, ME 04469-5775 USA; 12 Kyoto University, 46 Shimoadachichou, Sakyoku, Kyoto, 606‐8501 Japan; 13 Fenner School of Environment and Society, The Australian National University, Canberra, ACT 2601 Australia; 14 Faculty of Forest Science and Ecology, Vytautas Magnus University, Studentu˛ g. 13, Akademija, Kauno r., 53362 Kaunas, Lithuania; 15 Market Economics Ltd, Digital Basecamp, 1132 Hinemoa Street, Rotorua, 3010 New Zealand; 16 Institute of Forest Ecology, Slovak Academy of Sciences, Ľ. Štúra 2, 960 53 Zvolen, Slovakia; 17 Faculty of Forestry, Technical University in Zvolen, T.G. Masaryka 24, 960 53 Zvolen, Slovakia; 18 N. Laverov Federal Center for Integrated Arctic Research, Russian Academy of Science (FCIArctic RAS), 23 Northern Dvina Embankment, Arkhangel’sk, Russia 163000; 19 Social Science, Betty and Gordon Moore Center for Science, Conservation International, 2011 Crystal Drive, Suite 600, Arlington, VA 22202 USA; 20 Centro Austral de Investigaciones Científicas (CADIC), Consejo Nacional de Investigaciones Científicas y Técnicas (CONICET), Houssay, 200 (9410) Ushuaia, Tierra del Fuego Argentina; 21 Kola Biodiversity Conservation Center, Lenina st. 6-29, Apatity, Murmansk, Russia 184209; 22 Waldbau-Institut, University of Freiburg, Tennenbacherstr. 4, 79106 Freiburg, Germany; 23 World Resources Institute Africa, Madagascar Program, Hôtel Colbert, Business Center Area, 29 Lalana Printsy Ratsimamanga, BP 3884, 101 Antananarivo, Madagascar; 24 Forest Management and Development, Department of Environmental Sciences, Swiss Federal Institute of Technology (ETH) Zurich, Universitätsstrasse 16, 8092 Zurich, Switzerland; 25 Missouri Botanical Garden, Madagascar Research & Conservation Program, BP 3391, 101 Antananarivo, Madagascar; 26 Faculty of Geography, Ivan Franko National University of Lviv, Doroshenko Street 41, L’viv, 79000 Ukraine; 27 Institute of Biodiversity and Ecosystem Research, Bulgarian Academy of Sciences, 2 Gagarin Street, 1113 Sofia, Bulgaria

**Keywords:** Biodiversity conservation targets, Green infrastructure, Governance effectiveness, Landscape approach, Matrix effects, Policy instruments

## Abstract

Exploitation of natural forests forms expanding frontiers. Simultaneously, protected area frontiers aim at maintaining functional habitat networks. To assess net effects of these frontiers, we examined 16 case study areas on five continents. We (1) mapped protected area instruments, (2) assessed their effectiveness, (3) mapped policy implementation tools, and (4) effects on protected areas originating from their surroundings. Results are given as follows: (1) conservation instruments covered 3–77%, (2) effectiveness of habitat networks depended on representativeness, habitat quality, functional connectivity, resource extraction in protected areas, time for landscape restoration, “paper parks”, “fortress conservation”, and data access, (3) regulatory policy instruments dominated over economic and informational, (4) negative matrix effects dominated over positive ones (protective forests, buffer zones, inaccessibility), which were restricted to former USSR and Costa Rica. Despite evidence-based knowledge about conservation targets, the importance of spatial segregation of conservation and use, and traditional knowledge, the trajectories for biodiversity conservation were generally negative.

**Supplementary Information:**

The online version contains supplementary material available at 10.1007/s13280-021-01628-5.

## Introduction

More than 150 years ago, Marsh (1864) highlighted the negative effects of human actions on the environment. Almost a century later, Thomas (1956) delivered another seminal milestone addressing the need to cope with the human footprint on landscapes. Their conclusion that our planet is not “healthy”, and that the trends in environmental conditions are negative, has not changed. In fact, repeatedly over the past half century, international, national, and business policies have continued to highlight the need to conserve biodiversity and natural capital, and terms as ecosystem or landscape services, or nature’s contributions to people (e.g., Angelstam et al. 2019). For example, the Convention on Biological Diversity (CBD 2002) stated that the international aim was “to achieve by 2010 a significant reduction of the current rate of biodiversity loss” (Walpole et al. 2009; Sachs et al. 2009). In this context, Butchart et al. (2010) compiled trend data from 1970 to 2010 for 31 indicators of state, pressure, and response. They found that biodiversity state indicators, such as species’ population trends, habitat extent, and condition had declined, whereas indicators of pressures on biodiversity such as resource consumption and overexploitation had increased. Thus, despite responses such as more protected areas and new sustainable forest management policies, the rate of forest biodiversity loss had not slowed down. Butchart et al. (2010) concluded that “… efforts to address the loss of biodiversity need to be substantially strengthened by reversing detrimental policies, fully integrating biodiversity into broad-scale land use planning…”. According to IPBES (2019), the European Commission (2020) and Secretariat of the Convention on Biological Diversity (2020) this challenge remains. Two key tasks are to define performance targets and planetary boundaries for safe operation (e.g., Svancara et al. 2005; Rockström et al. 2009; European Commission 2021), and approaches to stewardship toward ecological, economic, and social sustainability (e.g., Steffen et al. 2011). This calls for assessments in terms of diagnosing the consequences on the ground in social–ecological systems (Rauschmayer et al. 2009; Angelstam and Elbakidze 2017).

Creation of protected areas that form functional habitat networks as a tool to support biodiversity conservation in the context of sustainable forest management is crucially important. Increased and expanding demands for natural resources in space and time have created frontiers of land use and land cover change, which has triggered the creation of different kinds of protected areas and other effective area-based conservation measures (Dudley 2013). This “protected area frontier” can be viewed as a response to the loss of natural and semi-natural habitats.

Forests form a prime example of a land cover that provides multiple natural resources and other benefits. Transforming naturally dynamic forest landscapes through management for wood production and deforestation for agriculture can take a long time and has a long recurring history of being replicated globally (e.g., Thomas 1956; Angelstam et al. 2021a). Williams (2003, p. 146) highlighted two “theaters of action” based on the connection between demand and supply, which were linked by flow of wood using seas and other waterways, and later by expanding frontiers of forest use and value-added production.

The first action is focused on regional centers of strong economic development. Deforestation to satisfy both local demands for pasture and agricultural land, and regional demands for wood, therefore, has a very long history in some European regions. For example, Anatolia in Turkey had 60–70% forest cover ca. 4000 years ago, but as a result of grazing, harvesting, fires, and spread of agricultural lands, this has declined to 26% today (Colak and Rotherham 2006) and area-demanding species became extirpated. Similar patterns occurred when agriculture expanded in China over the past four millennia (Elvin 2004). The expansion continued in northern China during the Xin dynasty in the eighteenth century, which resulted in the reduction of wildlife, deforestation, and changed hydrological regimes (Reardon-Anderson 2000). Comparing the eastern and western extremes of the Eurasian continent, Saito (2009) found that deforestation rates were homogenous according to the range expected from varying rates of human population growth.

The second theater of action can be related to the subsequent expansion toward global peripheries. Because most of the northern boreal forest rivers drain away from markets into the Arctic in both Russia and Canada, the rivers that flow toward markets were of special importance as they allowed long-distance transport of bulky natural resources such as wood (e.g., Lotz 2015). The industrial revolution in Western Europe thus triggered wood mining in intact forest landscapes in Eastern Europe (Naumov et al. 2016, 2018), as well as selective felling of white pine along the St. Lawrence River in North America (Greeley 1925). Such expanding frontiers that reduce naturalness are profoundly active also in tropical forests (Margono et al. 2014). Thus, the areas of remnant forest with higher levels of naturalness, and intact forest landscapes in particular, are shrinking globally (e.g., Watson et al. 2018), except where inaccessibility due to remote location or rough terrain offers protection. At the same time, connectivity among such remnants is poor (Ward et al. 2020), and “forest transitions” increase the area of planted forests with low levels of naturalness in the matrix surrounding remnant natural areas (FAO FRA 2020). The net effects on biodiversity are, therefore, negative (e.g., Angelstam and Manton 2021).

The Convention of Biological Diversity’s Aichi target #11 of 17% protected areas is a negotiated quantitative conservation target (CBD 2010), with input from evidence-based knowledge from conservation biology and landscape ecology (e.g., Wiens et al. 2006), about how much habitat is sufficient for conservation of viable populations of species. This target for protected areas and other effective area-based conservation measures has also qualitative criteria (e.g., effectively and equitably managed, representative for different ecoregions, well-connected, integrated CBD 2010). Visconti et al. (2019) identified and discussed four problems with Aichi Target #11 that have contributed to its limited achievement. These were (1) new protected areas being established mainly in locations that are less important for biodiversity, (2) effectiveness of protected areas not being measured as biodiversity outcomes, but as staff, equipment, law enforcement and type of management, (3) ambiguous representation of ecosystems, and (4) national-level contributions to the total global ambition being difficult to estimate, for example because of different portfolios of protected area categories.

The aim of this study is to document barriers and bridges regarding the contribution of different types of protected areas and other set-asides to functional habitat networks, which affect the opportunity to conserve biodiversity, and provide broad portfolios of ecosystem services. Is there a positive, neutral or negative net effect of protected area versus forest exploitation frontiers? We focus on exploring the situation and approaches in 16 case study areas located in boreal, temperate and tropical forest regions on five continents.

## Materials and methods

### Framework, case studies and policy implementation questions

Spatial planning to support the conservation, management and restoration of functional habitat networks can be divided into strategic, tactical and operational steps. This study focuses on a diagnostic assessment of protected area systems as a base for strategic biodiversity conservation planning in entire landscapes. In the context of diagnosing the state of protected areas and the functionality of the habitat networks they aim at forming, both the pressures affecting their state, and the responses to both states and pressures, need to be addressed (e.g., Butchart et al. 2010). We use CBD’s Aichi target #11 quantitative and qualitative criteria (Table 1) as a normative model (cf. Hong and Shim 2018; Angelstam et al. 2020a). This target is consistent with policy about green infrastructures (GIs) for biodiversity conservation and human well-being (e.g., European Commission 2013).

**Table 1:** Examples of foundation papers for the Aichi target #11. Other Aichi targets are of equal importance and complement each other; e.g., Target 14: “By 2020, ecosystems that provide essential services, including services related to water, and contributed to health, livelihoods and well-being, are restored and safeguarded”, and Target 15: “By 2020, ecosystem resilience and the contribution of biodiversity to carbon stocks have been enhanced, through conservation and restoration, including restoration of at least 15 percent of degraded ecosystems, thereby contributing to climate change mitigation and adaptation and to combating desertification”

	Wording in CBD’s target #11	Examples explaining the rationale
Quantitative target	At least 17% of terrestrial and inland water areas and 10% of coastal and marine areas, especially areas of particular importance for biodiversity and ecosystem services	Andrén (1994), Svancara et al. (2005), and Fahrig (2003) all focus on fragmentation thresholds and performance targets
Qualitative targets	Effectively and equitably managed	Antrop (2000) and Wiens et al (2006)
	Ecologically representative	Nilsson and Götmark (1992)
	Well-connected systems of protected areas and other effective area-based conservation measures	Taylor et al. (1993)
	Integrated into the wider landscape and seascape	Hobbs et al. (1993) and Wiens et al (2006)

**Fig. 1:**
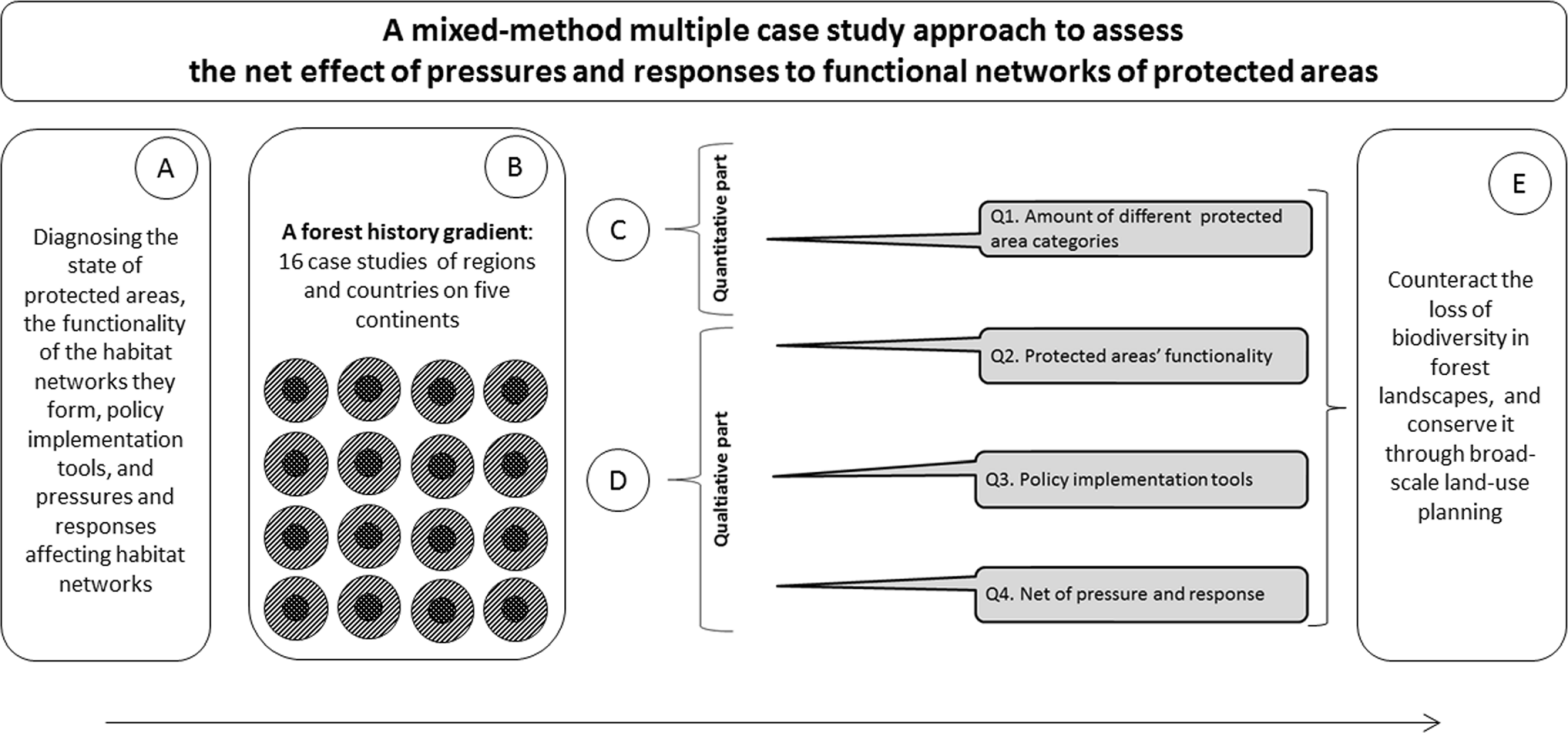
Overview of the research process from the general aim (A), through the selection of countries and regions case study areas (B), as well as the quantitative and qualitative methods (C, D) and four research questions, all aiming at counteracting the loss of biodiversity in forest landscapes, and conserve it through broad-scale land-use planning. Finally, E lists the key topics for discussion

In Fig. 1, we present an overview of our comparative mixed-method approach built on multiple case study area narratives written by the co-authors who are experts on the topics addressed in the different case study countries and regions selected (see Appendix S1, from which data were extracted, see e.g., Angelstam et al. 2021a, b). In this study the co-authors were academic experts involved with research or conservation, or both, with in-depth knowledge of the 16 case study regions, respectively. Together with their professional networks they produced comprehensive accounts of relevance for this study, and consulted a wide large of peer-review and gray literature (*n* = 282), all quoted in the Appendix S1. This approach was inspired by Rapid Rural Appraisal, which aims at learning in a cost-effective manner. This implies ignoring what Chambers (1981, 1994) terms “inappropriate professional standards” because they are too costly. Instead another rigor is applied, which is based on the two principles of “optimal ignorance” (knowing what it is not worth knowing), and “proportionate accuracy” (recognizing the degree of accuracy required).

**Fig. 2:**
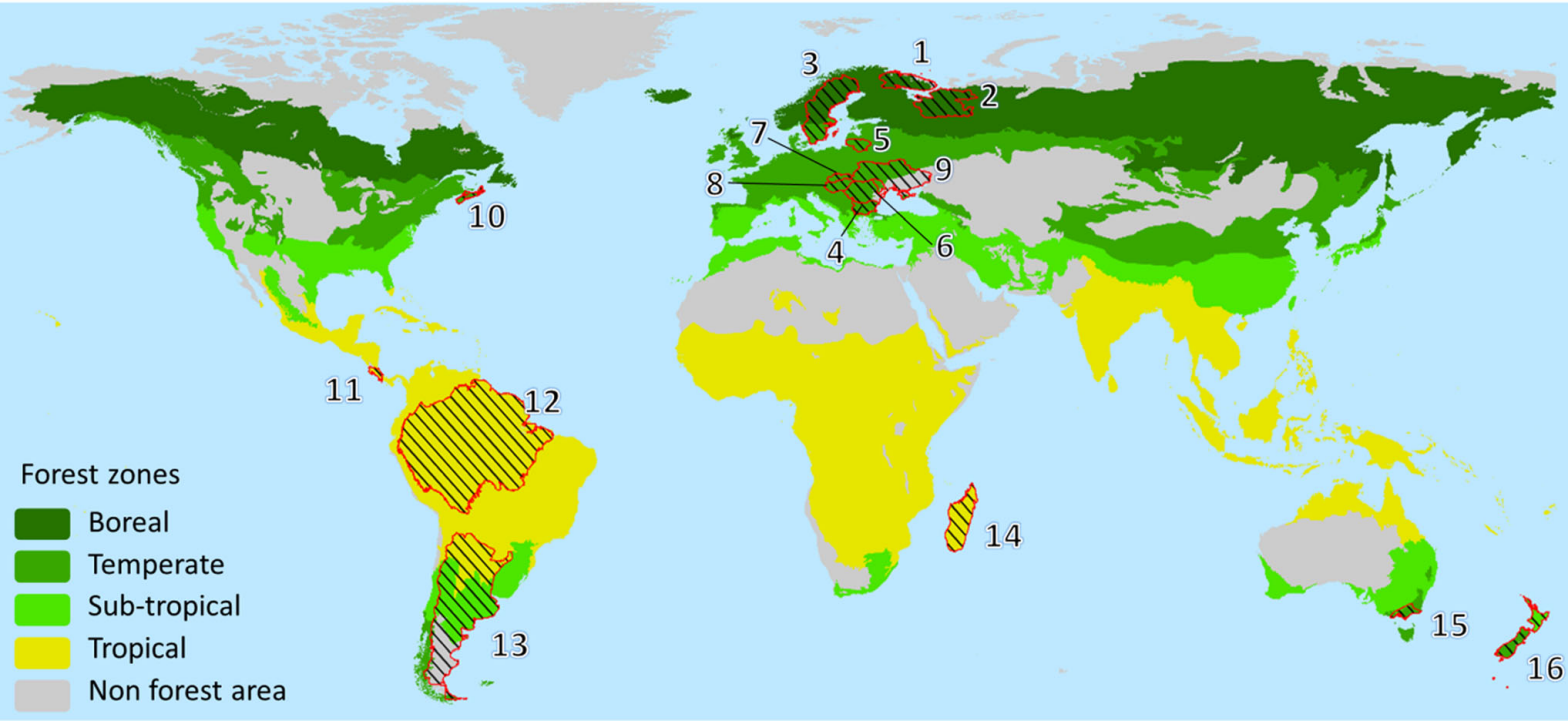
Map showing the location of the 16 case study areas, and where forests and woodlands in green form the potential natural vegetation based on ecofloristic zones (FAO 2000). These areas were selected to cover the deforestation gradient on the European continent (top) ranging from those with some remaining intact forest landscapes [Murmansk (1) and Arkhangelsk (2) regions in NW Russia, Sweden (3)], areas still having a high proportion of forest [(Bulgaria (4), Lithuania (5), Romania (6), Slovakia (7)], and fragmented forests [(Hungary (8) and Ukraine (9)]. Additionally, the province Nova Scotia in Canada (10), Costa Rica (11), the Amazon Biome (12), Argentina (13), Madagascar (14), SE Australia (15), and New Zealand (16) were selected. The numbers refer to the country column in Table 2

To address the aim (Fig. 1A), 16 case studies were selected (Figs. 1B, 2) and both quantitative and qualitative methods were applied (Fig. 1C, D) to address four questions (Q1–4). We mapped the protected area categories employed in each case study and compiled the area proportions of these categories (Q1); reviewed if and how Aichi target #11’s qualitative criteria (e.g., effectiveness, representativeness and connectivity) are addressed (Q2); and mapped the types of policy instruments applied to implement the establishment of protected areas(Q3); and assessed the net effect of pressures and responses on the state of protected areas as habitat networks supporting biodiversity conservation in entire landscapes (Q4). Finally, we discuss how to counteract the loss of biodiversity in forest landscapes, and maintain biodiversity through broad-scale land-use planning (Fig. 1E).

**Table 2:** Overview of the 16 case study areas’ cover of forest, plantations, other land covers, and water (see Appendix S1). Numbers in brackets refer to Fig. 2. Data extracted from the Appendix S1

Continent	Country or biome	Region or other entity	Total area (km^2^)	Land area (% of total)	Forest area (% of land)	Plantation area (% of land)	Other land (% of land)	Water (% of total area)
Europe	Bulgaria (4)	All	110 993	98	34	0	65	2
	Hungary (8)	All	93 030	96	11	11	82	4
	Lithuania (5)	All	65 300	99	36	0	65	1
	Romania (6)	All	238 397	97	29	5	69	3
	Russia (2)	Arkhangelsk*	413 400	98	57	0	43	2
	Russia (1)	Murmansk	144 900	92	44	0	55	8
	Slovakia (7)	All	49 035	99	41	0	59	1
	Sweden (3)	All	450 295	92	67	0	31	8
	Ukraine (9)	All	603 628	99	19	0	82	1
America N	Canada (10)	Nova Scotia	55 284	96	87	0	18	4
	Costa Rica (11)	All	51 100	99	76	2	24	1
America S	Amazon Biome (12)	Brazil 59%, Peru 11%, Colombia 8%	6 800 000	98	14	0	88	2
	Argentina (13)	All	2 780 400	98	78	0	20	2
Africa	Madagascar (14)	all	591 144	99	29	0	72	1
Australia/Oceania	Australia (15)	Victoria	237 659	96	27	1	72	4
	New Zealand (16)	All	268 021	98	36	6	59	2

*Excluding Nenets okrug

When focusing on particular regions or countries as units of policy and government, a mixed-method multiple case study approach is suitable. Following the terminology of Stake (2003) each unit of study in this article is a “bounded” separate entity hosting a particular portfolio of environmental histories. With a multiple case study area approach, one can do in-depth exploration of a specific bounded system (Yin 2002), and relate those to differences in policy instruments and their implementation, as well as phases of forest landscape development among the case study areas. Based on 16 different case study areas as a “collective case design”, with several instrumental bounded cases, we aimed to produce an in-depth exploration of the net result of pressures and responses affecting the state of protected areas as green, or ecological, infrastructures for biodiversity conservation.

Nine Pan-European case study areas were selected to mirror the gradient from the last Intact Forest Landscapes in the north (Potapov et al. 2008; Watson et al. 2018) via regions with contiguous forest cover (> 50%) and fragmented forests (20–50% forest cover) to regions that have < 20% forest cover in the south (see Angelstam et al. 2021a) (Fig. 2; Table 2). Two case study areas (Arkhangelsk and Murmansk) are regional subjects of the Russian Federation in NW Russia, and the other are the countries Sweden, Lithuania, Slovakia, Romania and Bulgaria, as well as Hungary and Ukraine. Additionally, seven case study areas were chosen from four other continents including North and South America (the province Nova Scotia in easternmost Canada, Costa Rica, the Amazon Biome covering parts of nine countries, and Argentina), Africa (Madagascar), and Australia/Oceania (a region in the Australian state Victoria, and New Zealand) (Fig. 2; Table 2). For each of the 16 selected case study areas we address four policy implementation questions regarding CBD’s Aichi target #11:

Question 1. What are the protected area categories, and their area proportions in relation to the quantitative target of 17%?

Question 2. To what extent are the qualitative criteria of the Aichi target #11 (e.g., effectiveness, representativeness and connectivity) satisfied?

Question 3. What are the roles of different policy implementation tools?

Question 4. What negative and positive factors affecting the effectiveness of biodiversity conservation and integration into the wider landscape of protected areas and their matrix?

#### Protected area categories and their area proportions (Question 1)

We compiled the portfolios of conservation instruments aiming at biodiversity conservation through the maintenance of representative habitat networks that can sustain viable populations of naturally occurring species. We focus on four groups of conservation instruments matching IUCN categories (Dudley 2013): formally protected (IUCN categories I, II, III, IV) and multiple use areas (IUCN categories V, VI), and if relevant also other set-asides, such as forests with protective functions, buffer zones and unproductive unmanaged forests. In addition, we quantified the area proportions of these categories using official statistics (see Appendix S1).

#### Functionality of protected areas (Question 2)

Inspired by the qualitative criteria of CBD’s Aichi target #11, to estimate the effectiveness of the conservation instruments, we relied on the contributors of the case study narratives to address Question 2 for each case study area. We included several approaches to assess effectiveness, including protected areas’ size, duration, decision-making processes, control and method for monitoring, which can vary considerably (e.g., Angelstam et al. 2020a). To address representativeness, especially if the case study area had large ecoregional variation, we estimated the contributions to Aichi targets #11 at both coarser (e.g., national, regional) versus finer (e.g., ecoregions) scales. To address functional connectivity of protected areas the proportion of any land cover of a particular quality that satisfies both minimum patch size requirements (e.g., forest stands) and sufficient patch density to form a functional habitat network (e.g., tracts) for a focal species can be used as such a “correction factor” (e.g., Angelstam et al. 2011, 2020a). The role of silvicultural systems (e.g., Duncker et al. 2012) in the managed forest matrix is an additional factor—how well do forest management systems match natural forest disturbance regimes (Attiwill 1994)? This can also be viewed as a conservation practice.

#### Portfolios of policy instruments (Question 3)

Different protected area categories and other set-asides supporting biodiversity conservation can be viewed as tools of action to implement biodiversity policy by overcoming problems and achieving objectives. To classify policy implementation instruments, we adopt the trichotomy of economic (carrots), regulative (sticks), and informational (sermons) instruments advocated by Vedung (1998). Economic instruments may include subsidies, certification schemes and premiums; regulative instruments may include rules, restrictions and control; and informational may include training, extension and information campaigns. Following Brukas and Sallnäs (2012) the contributors to each case study area text presented in the Appendix S1 assessed the relative importance of economic, regulative, and informational tools by distributing a total of 10 points, the results of which was presented as a star diagram.

#### Net effect of protected areas and their surrounding matrix (Question 4)

For each case study area the contributing authors endeavored mapping of different kinds of pressures on protected areas and habitat network functionality on the one hand, and responses in terms of improved satisfaction of CBD’s Aichi target’s #11 quantitative and qualitative criteria on the other. This was then summarized in tabular form.

## Results

### Protected area categories and their area proportions (Questions 1)

**Fig. 3:**
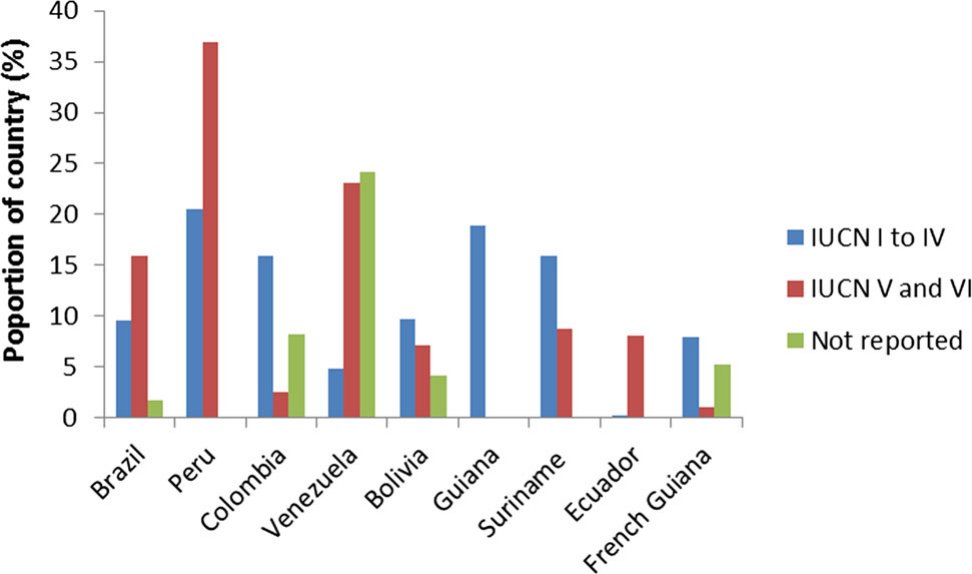
Illustration of the diverse portfolios of protected area categories according to IUCN in the Amazon Biome’s nine countries ranked from the largest (Brazil with 4 050 000 km^2^) to the smallest (French Guiana 90 000 km^2^) (data from Prüssmann et al. 2017). This makes comparisons of the area proportions of different protected area categories difficult

A wide range of conservation instruments have been employed in the 16 case study areas, and their forest proportions varied widely (Table 3). The situation in the Amazon Biome’s 9 countries illustrates this. While the average proportion of areas with higher levels of protection (IUCN categories I to IV) and those focused on multiple use (IUCN V and VI) was 12% and 11%, respectively, different countries in the Amazon Biome had widely different portfolios of protected area categories (Fig. 3 and Appendix S1). This means that attempts to add different percentage points without attempting to address what different categories imply on the ground are not meaningful.

**Table 3:** (Q1) Types of area protection and their proportion of current native forest cover in the 16 case study areas (numbers in Appendix S1 rounded to integers). Note that the figures cannot be summed because the different categories have different meanings and may overlap spatially (see Q2). Data extracted from the Appendix S1

Continent	Country or biome	Region or other entity	Formal protection (%) (IUCN I to IV)	Restoration/management (%) (IUCN V to VI)	Voluntary set-aside (%)	Protective forests (%)
Europe	Bulgaria	All	6	3	0	0
	Hungary	All	22	~ 20**	4	0
	Lithuania	All	9	3	NA	15
	Romania	All	3	0	21	NA
	Russia	Arkhangelsk*	9	0	1	23
	Russia	Murmansk	30	0	0	11
	Slovakia	All	23	26	0	17
	Sweden	All	8	4	2	0
	Ukraine	All	7	0	0	NA
America N	Canada	Nova Scotia	13	5	NA	NA
	Costa Rica	All	33	3	NA	NA
America S	Amazon Biome	Brazil 59%, Peru 11%, Colombia 8%	12	11	NA	NA
	Argentina	All	5	0	17	0
Africa	Madagascar	all	23	20	NA	NA
Australia/Oceania	Australia	Victoria	20	NA	NA	NA
	New Zealand	All	77	NA	3	NA

*Excluding Nenets okrug

**Natura 2000 nominations cover 40% of Hungary’s forests, half of which are also under national protection

Some countries have chosen ways of assigning conservation instruments. For example, as reviewed in Angelstam et al. (2020a), in Sweden officially acknowledged contributions to the pool of “protected” areas for biodiversity conservation have changed over time. Initially, only formally protected areas were considered as conservation area assets (Angelstam et al. 2011). However, currently also voluntary set-asides under forest certification programs, as well as retention tree groups on harvested areas, and unproductive forests (producing < 1 m^3^ ha^−1^ of wood year^−1^), are officially included in estimates of the amount of protected areas (Table 4). This can determine whether or not agreed performance targets are met.

**Table 4:** Basic information about four groups of conservation instruments officially considered as protected areas in Sweden, including two types of formal protection, voluntary set-aside areas, nature consideration areas, and unproductive forests (from Angelstam et al. 2020a)

	(i.i) Formal according to the Environmental Code	(i.i) Formal; according to the Environmental Code	(i.ii) Formal; according to the Land Code	(ii) Voluntary set-aside	(iii) Nature considerations (§ 30, Forestry law)	(iv) Unproductive (< 1 m^3^ ha^−1^ year^−1^; § 13a, Forestry law)
Area and proportion of all forest land in 2019		(i.i and i.ii) 2335 × 10^3^ (8.3%)		(ii) 426 × 10^3^ (1.5%)	(iii) 426 × 10^3^ ha (1.5%)	(iv) 3239 × 10^3^ (11.5%)
Aim	National park, nature reserve: conserve and develop nature of high value for plants, animals and people	Biotope protection: conserve terrestrial or aquatic habitat for threatened species	Conservation agreement: conserve and develop qualities for biodiversity	A complement to formal protection	Consideration to biodiversity conservation in managed forest	Wood harvest not recommended
Establishment	1909 and 1964, respectively	1998	1993	1995	1979	1979
Target size	Usually > 20 ha	Usually < 20 ha	Variable	> 0.5 ha	< ca 0.5 ha	> 0.1 ha
Duration	Permanent	Permanent	Variable	Unknown	Unknown	Permanent
Decision by	Parliament, Government, County, Municipality	Forest Agency, Municipality	Agreement between the State or Municipality and owner	Land owner	Parliament, Government, Forest Agency	Parliament, Government
Control	County	Forest Agency, Municipality	State	Forest certification	Forest Agency	Forest Agency
Monitoring	Georeferenced GIS polygons	Georeferenced GIS polygons	Georeferenced GIS polygons	GIS data and questionnaires	Random field sampling	National Forest Inventory

### Functionality of protected areas as habitat networks (Question 2)

The observations from the case study areas can be viewed as a horizon scanning of different factors hampering the effectiveness of protected areas as parts of habitat networks for species populations, and where necessary habitats and ecological processes can be sustained. Representativeness (1), habitat quality (2), functional connectivity (3), what kinds of resource extraction is allowed in protected areas (4), long time needed to deliver habitat by restoration (5), “paper parks” (6), “fortress conservation” (7), and lack of open access data about protected areas (8) were eight examples of factors highlighted in the 16 case studies (Table 5).

**Table 5:** (Q2) Distribution of eight factors affecting effectiveness of protected areas and networks (i.e., green infrastructure) among the16 case study areas. Data extracted from the Appendix S1

Continent	Country or biome	Region or other entity	Number of ecoregions	Variation in PAs’ among ecoregions (%)	Limited habitat quality	Poor connectivity	Logging in PAs	Long time for restoration	“Paper parks”	“Fortress conservation”	No spatial data
Europe	Bulgaria	All	3	2–12	1	1	1	1			1
	Hungary	All	1	NA	1	1	1	1	1		
	Lithuania	All	1	NA	1	1	1	1	1		
	Romania	All	5	4–28			1				
	Russia	Arkhangelsk	1	NA		1			1	1	
	Russia	Murmansk	2	NA					1		
	Slovakia	All	2	23–44	1	1	1	1		1	
	Sweden	All	3	7–48	1	1		1			
	Ukraine	All	5	11–29	1	1	1	1	1		
America N	Canada	Nova Scotia	1	NA							
	Costa Rica	All	12	NA							
America S	Brazil 59%, Peru 11%, Colombia 8%	Amazon Biome	36	2–23			1		1	1	
	Argentina	All	9	0–20		1			1		
Africa	Madagascar	all	5	1–63	1	1	1	1	1	1	
Australia/Oceania	Australia	Victoria	1	NA	1	1		1	1		
	New Zealand	All	2	48–93	1	1		1		1	

First, regarding representativeness of different forest ecosystems, with ecoregions as a proxy, the number of ecoregions in each case study ranged from 1 (Hungary, Lithuania, Canada with Nova Scotia, Australia with the state of Victoria’s mountain ash forests) to 36 (the Amazon Biome). Based on estimates from nine of the case study areas of the proportion of protected areas representing IUCN categories I to IV, the variation among ecoregions was considerable (Table 5). The pattern in common was that the least suitable ecoregions for forestry and forest clearing aiming at sustained yield forestry and agriculture (i.e., those at higher altitudes and latitudes) had a higher proportion of protected areas. Thus, in Argentina, Sweden and Ukraine areas of limited interest for forestry intensification “help” making the national figures for protected area amounts high (see Appendix S1). While in Ukraine’s Carpathian and Crimean mountains 8% is protected, the proportion declines with increasing historic deforestation impact among ecoregions to about 1% protected (see Appendix S1).

Second, habitat quality in terms of low levels of forest naturalness reduces effectiveness of protected areas and habitat networks for biodiversity conservation. In countries with a long history of forest use the proportion of strictly protected forests is low. For example, in Hungary only 1.8%, in Bulgaria < 2.0% and in Slovakia 0.5% have high levels of naturalness judged by their old-growth character.

Third, for a given amount and habitat quality of individual protected areas, habitat network functionality depends on their size and spatial configuration. Attempts to estimate the proportion of areas that form functional habitat networks have been made for different taxa (e.g., Angelstam et al. 2011; Abrego et al. 2015; Nordén et al. 2018). For example, using evidence-based knowledge about focal resident bird species Angelstam et al. (2020a) estimated the amount, regional representation, and functional connectivity of all mapped forest patches with high levels of naturalness in Sweden. The resulting habitat networks were validated using independent field surveys of focal bird species. Finally, they assessed fulfillment of international and national conservation targets of 17–20% protected areas in functional habitat networks among Swedish ecoregions. Even if 31% of forest land in all Sweden is formally protected and voluntarily set-aside, or not used for wood production now and in the future (Table 4), they showed that applying representation and connectivity criteria, as well as an estimate of habitat quality for unproductive forests, reduced this figure to an effective GI of 12%. When disaggregating the different ecoregions the effective GI was 54% for the sub-alpine forest ecoregion, which hosts EU’s last intact forest landscapes (Jonsson et al. 2019). However, the figures were only 3–8% of the ecoregions where the focus is on wood production. In Sweden there are thus both industry-driven narratives and evidence-based interpretations regarding the extent to which Aichi target #11 is satisfied.

Fourth, in several categories of protected areas wood harvesting takes place (see Appendix S1). For example, in Hungary’s specially protected forests, shelterwood and clear-cutting systems are applied to 48% of them, and in 29% regular timber extraction is prohibited. In other protected forest types more aimed at multiple use, the corresponding figures are 78% and 10%, respectively. While this can be justified as a type of conservation management to restore naturalness components such as dead wood and foliage height diversity, the aim can also be to extract wood. Similarly, 29% of the forest area in Romania is under uncertain protection status because intensive regeneration treatments and clear cuts are allowed. Both Slovakian and Lithuania National parks vary from the strict protection of the westernized National Parks approach (Lockwood et al. 2012) and undergo regular forest management, and nature conservation bodies can usually participate in the planning. However, the forest department makes the final decision. It should, however, be noted that protected areas in Central Europe aim at conserving cultural woodland landscapes, the conservation of which may require wood harvesting (Angelstam et al. 2021a).

Fifth, the time needed to deliver habitat by landscape restoration management is generally much longer than regular forest rotations. For example, in Bulgaria there were attempts in “forests designated to old-growth transformation” to introduce uneven-aged silvicultural systems with preservation of some old-growth elements (e.g., dead wood and biotope trees). Retention forestry is another widespread practice (Shorohova et al. 2019). However, the survival of retention trees and coarse woody debris in different decay stages is low and has limited effects on forest naturalness at the landscape level (e.g., Jonsson et al. 2016).

Sixth, effectiveness is related also to the governance of protected areas and networks. The problem of ‘paper parks’ refers to protected areas that are officially designated, but because of a weak protection regime do not provide effective biodiversity conservation. For example, in Romania the overlap between the protected area network already established prior to joining the EU and adopting the Natura 2000 system reaches 96%, meaning that the introduction of Natura 2000 has by and large been redundant. In Sweden the overlap is 90% and in Hungary ca. 50%. Moreover, the level of protection provided by the EU Natura 2000 system remains ambiguous, and the whole system can be deceiving in terms of its effectiveness to secure sufficient amounts of high quality forest habitats, particularly for specialist species (e.g., Nagel et al. 2017).

Seventh, the problem of “fortress conservation” relates to protected areas where ecosystem function is viewed without considering other human activities, and local communities are often viewed as poachers or squatters using nature in destructive ways that threaten biodiversity (Mikhailova and Efimov 2015), or they are not able to utilize the forest resources in a sufficient manner to legally secure their livelihoods because of the strict regulatory instruments and lack of alternative income sources (e.g., subsidies, compensations). In the EU, The Romanian case study stands out in this regard, as the poverty of human communities in remote mountain areas may represent an underlying factor that motivates inadequate forest use practices. Another example is New Zealand, where the society and governing bodies achieved a tremendous conservation goal between the 1970s and late 1990s by completely stopping exploitative logging activities in native forests and protecting more than 3/4 of the remnant area. One of the open questions is how to maintain the existing second-growth native forest and shrubland cover on private and Māori land that is not adequately protected, without impeding the opportunities for sustainable economic development of rural communities. For example, the proportion of Māori land covered with native forest and shrubland is much higher than any other land, apart from areas in public conservation land (see Appendix S1). Development of future conservation strategies for these forests will require a careful consideration of the social–ecological context, especially how decisions on protecting and managing biodiversity might impact the use and development of Māori land. Through New Zealand’s history a range of hurdles impeding the full and optimal use of Māori land for economic development have arisen. Moreover, native forests represent a central role in their culture and values, which determine their relationship with the natural environment and how they utilize it. Therefore, deploying a set of stringent protection measures, as the ones in public conservation forests, and without providing for activities could unfairly impact on Māori communities and worsen disadvantages created by historic confiscation and loss of land. Similarly, in Australia forests are part of the original estate of Aboriginal people, wrested from them during this country’s period of colonial history. The remnants left behind, now mostly in state-owned timber production forests and forested conservation reserves, have acquired a level of significance often attributed to a commodity that is rare (Purdie and Cavanagh 1993). In other cases, land owners question the value of nature conservation and claim that they can reach nature conservation goals by traditional management aimed at wood production. At the other extreme, cultural landscapes based on animal husbandry and multi-functional woodland management depend on anthropogenic disturbances, and occur on all continents with forest.

Eighth, transparent assessment of effectiveness can be hampered by limitations in the existence or availability of both spatial and attribute data concerning protected areas, and the matrix surrounding them. This applies to voluntarily set-aside areas in the context of forest certification both in Sweden and Ukraine. Additionally, there may be spatially overlapping denominations, which represent different conservation instruments and different levels of governance. This means that if summed, the total area of overlapping protected area nominations will exceed the existing physical area (Svensson et al. 2020). Moreover, making available the location of formal set-asides may be considered as intruding on private ownership, and cadasters for land ownership may not exist, or not be public.

**Fig. 4:**
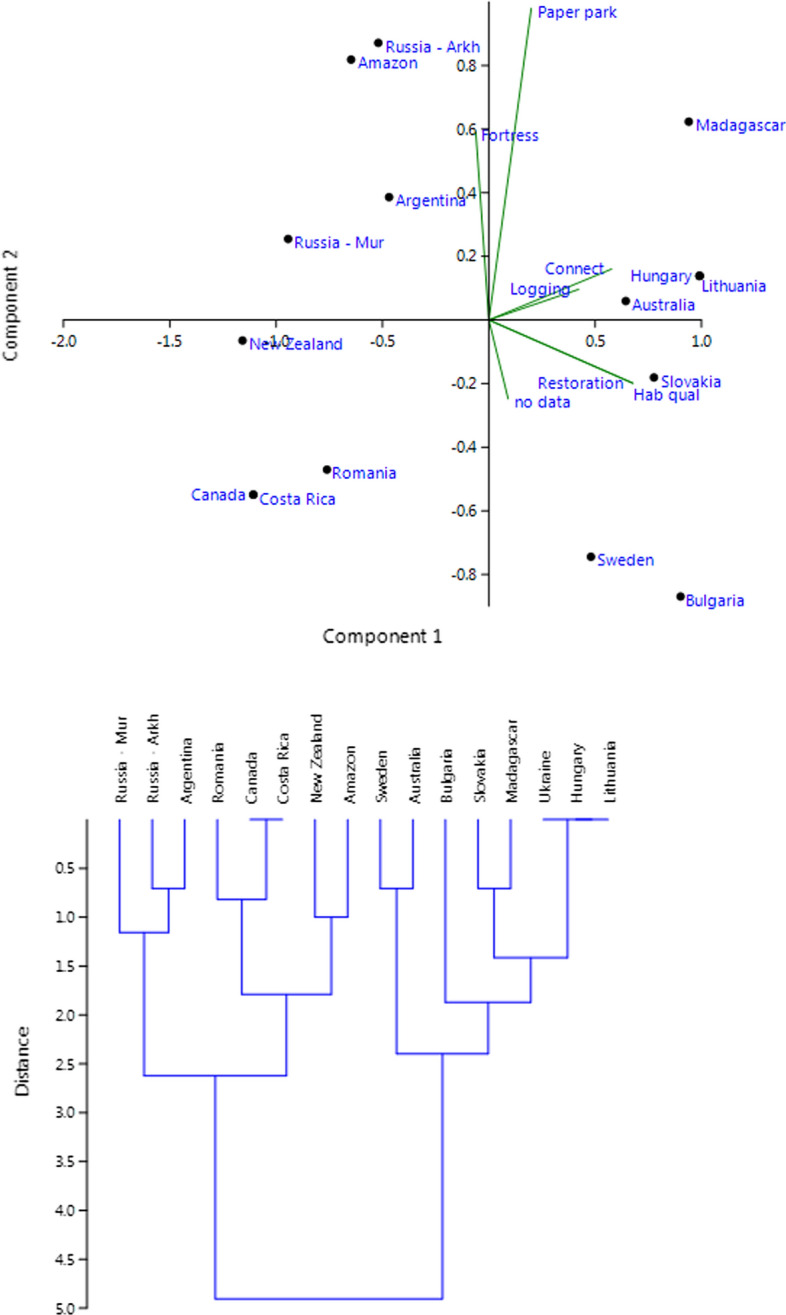
(Q2) PCA ordination and clustering based on variables in Table 5

In an exploratory PCA ordination using all these variables, except the number of ecoregions (Fig. 4), PC1 had an Eigenvalue of 0.80 and explained 50% of the variance. Positive loadings included the variables Habitat quality, Connectivity, Logging and Restoration. PC2 included the variables Paper Park and Fortress conservation, and had an Eigenvalue of 0.28 and explained an additional 18% of the variation. This resulted in two distinct clusters with countries (i) having a long history of alteration of potential natural forest vegetation and deforestation, and (ii) those with a shorter history of forest landscape transformation.

### Portfolios of conservation policy implementation instruments (Question 3)

**Fig. 5:**
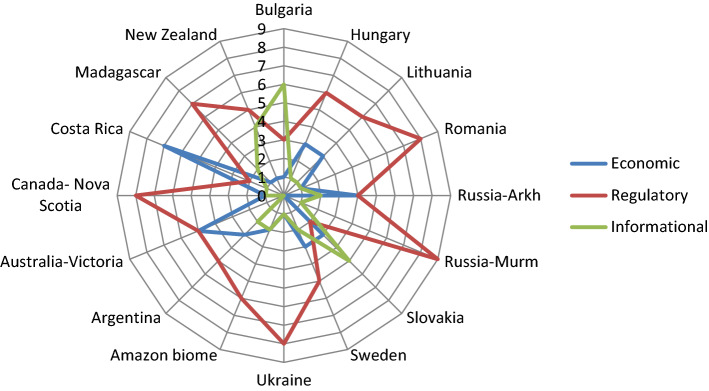
(Q3) Interpretation of narratives about case study regions and countries regarding how 10 points are distributed among economic, regulatory, and informational groups of policy instruments following Vedung (1998). Following Brukas and Sallnäs (2012), the contributors to each case study area assessed the relative importance of economic, regulative, and informational instrumentation by distributing a total of 10 points

Estimates of how the portfolios of different groups of policy instruments aiming at biodiversity conservation were distributed in the 16 case study areas are presented in Table 6. On average, the distribution of 10 attributed points estimated from the case study narratives among the three groups of policy instruments differ significantly (Table 6; Fig. 5; Kruskal–Wallis, df = 2, *χ*^2^ = 18.9, *p* < 0.0001), and regulatory instruments dominated (> 50%). This pattern was the same for the nine European versus the seven non-European case study areas. However, according to the case study narratives there were exceptions to the overall average pattern. While in Costa Rica economic policy instruments in terms of payment for ecosystem services dominated, in Bulgaria informational policy instruments dominated.

**Table 6:** (Q3) Estimates of how 10 points are distributed among the three groups of policy instruments based on interpretation of narratives about 16 case study regions and countries (see also Fig. 5). Data extracted from the Appendix S1

	Country or biome	Region or other entity	Economic”Carrots”	Regulatory”Sticks”	Informational”Sermons”	Sum
Europe	Bulgaria	All	1	3	6	10
	Hungary	All	3	6	1	10
	Lithuania	All	3	6	1	10
	Romania	All	1	8	1	10
	Russia	Arkhangelsk	4	4	2	10
	Russia	Murmansk	0	9	1	10
	Slovakia	All	2	3	5	10
	Sweden	All	3	5	2	10
	Ukraine	All	1	8	1	10
America N	Canada	Nova Scotia	1	8	1	10
	Costa Rica	All	7	2	1	10
America S	Amazon Biome	Brazil 59%, Peru 11%, Colombia 8%	2	6	2	10
	Argentina	All	3	5	2	10
Africa	Madagascar	all	1	7	2	10
Australia/ Oceania	Australia	Victoria	5	5	0	10
	New Zealand	All	1	5	4	10
Mean value			2.4	5.6	2.0

One of the Russian case study areas (Arkhangelsk oblast) illustrates how informal policy instruments in terms of internationally active environmental NGOs can foster integration of policy instruments representating all three groups of policy instruments. Forest management certification systems such as Forest Stewardship Council’s (FSC) are often considered as a ‘carrot’ for timber companies in some areas, which can gain access to environmentally sensitive markets. As opposed to eastern Russia this is true in the case of NW Russia, where the forest sector is focused on European eco-sensitive markets that require FSC certificates (Debkov 2019). In these cases ‘non-state market-driven forest governance systems’ (Cashore 2002) can play the role of a ‘stick’ simultaneously with state regulation. Thus, once a company has been certified, voluntary FSC standards are no longer voluntary. As a result, driven by environmental NGOs at regional to international levels, forest management and forest conservation practices in NW Russia are shaped by both state norms, as well as 'non-state market-driven' standards. For instance, FSC requires a forest owner to define and to exclude from forest exploitation core areas of the so-called “intact forest landscapes” (Yaroshenko et al. 2001), although this is not required by national law. Core areas are defined on maps combined with non-legally binding moratoria agreements have led to new areas protected by state agencies. The creation of a > 3000 km^2^ protected area in 2019 in SE Arkhangelsk region is a good example. This illustrates that state ownership can rapidly create protected areas.

### External effects on protected area frontiers (Question 4)

Based on our 16 narratives a total of seven negative and four positive factors in the matrix surrounding protected areas were identified (Table 7). The negative factors were increased harvest rates (1), improved road access (2), use and conservation clashes (3), untrustworthy forest data (4), no data about forest conditions (5), old forest decline (loss of naturalness, impact of exotic invasive organisms) (6), and mining, wind power, etc. (7). Positive factors were presence of protective forest zones (i) and buffer zones (ii), inaccessibility (iii) and habitat restoration (iv).

**Table 7:** (Q4) Distribution of external factors affecting effectiveness of protected areas and networks among the16 case study regions and countries. Negative contributions are placed to the left in darker shading and positive to the right with lighter shading

Continent	Country or biome	Region or other entity	Increased harvest	Improved road access	Use and conservation clash	Not trustworthy data	No data about forest states	Old forest declines	Mining etc	Protective forests	Buffer zones	Inaccessible	Habitat restoration
Europe	Bulgaria	All	1	1	1	1	1	1				1	1
	Hungary	All	1		1			1					1
	Lithuania	All	1		1	1	1	1		1	1		
	Romania	All	1	1	1	1	1	1					
	Russia	Arkhangelsk	1	1				1	1	1	1	1	
	Russia	Murmansk							1	1	1	1	
	Slovakia	All	1		1			1		1	1		1
	Sweden	All	1	1	1			1	1			1	1
	Ukraine	All	1	1	1		1	1		1	1	1	1
America N	Canada	Nova Scotia											
	Costa Rica	All	1	1	1					1	1		1
America S	Brazil 59%, Peru 11%, Colombia 8%	Amazon Biome	1	1	1			1	1			1	
	Argentina	All	1	1	1			1	1			1	
Africa	Madagascar	All	1					1					
Australia/ Oceania	Australia	Victoria	1		1								
	New Zealand	All			1			1				1	1
		Sums	13	8	12	3	4	12	5	6	6	8	7
		Group sum	57							27			

Regarding negative factors, harvest rates and volumes are increasing in countries in transition away from Soviet legacies, such as in Bulgaria, Hungary, Lithuania, Slovakia, Ukraine and Romania. There is also a spatial expansion of the transformation into natural and near-natural forests. In some case study areas frontiers of wood mining have already past (Russia’s Murmansk region described by Angelstam et al. 2020b), or continue to expand such as in Russia’s Arkhangelsk region (Karpov 2019) and in NW Sweden’s mountain forests (Svensson et al. 2019). Brazil’s Amazon Biome is the prime example.

Second, it is getting increasingly easier to negatively influence wilderness areas “beyond” frontiers of forest transformation. In the past, lack of technologies and resources guaranteed protection of forests in remotely located or otherwise inaccessible areas, which is still the case in parts of the NW Russian case study areas, and the Amazon Biome. Today, with much more advanced technologies and better road infrastructure, natural forest remnants in mountain regions have become more accessible, such as in Bulgaria, Romania and Ukraine.

Third, clashes between actors promoting intensified forest use and increased area protection are widespread. The ongoing debate in Sweden is an interesting example on how competing narratives over reality may develop (Mårald et al. 2017; Sténs and Mårald 2020). With terms like bio-economy, a new discourse is beginning to dominate the previous sustainable forest management discourse, which simultaneously considers economic benefits, biodiversity conservation and rural development (Pülzl et al. 2014). Thus, in Slovakia, harvest rates have increased since the 1990s and current levels of harvesting are expected to last until 2035 when the timber stock will decrease as a result of changing age structure of forests (Paluš et al. 2020). On the other hand, there is a demand to leave more forest without any human intervention, and to apply continuous forest cover forestry. National policies and discourses to legitimize different methods may thus alternate over time, depending on the government in power. Bolsonaro’s abandoning of Brazilian national policies that combined effective nature conservation, multiple use areas and recognition and protection of indigenous rights, is a return to past policies that prioritized economic objectives while largely ignoring biodiversity conservation needs.

Fourth and fifth, data may be ambiguous or absent. For example, there can be disagreement among forest stakeholders and actors how much forest is actually “protected”, and if conservation targets are met or not (Angelstam et al. 2020a). Examples of no data about the area exist in Bulgaria where there is no plot-based National Forest Inventory, and in Ukraine forest certification bodies cannot report where voluntary set-asides are located. The same lack of proper spatial data hinder transparent analyses related to protected forest area overlaps and dynamic in both Lithuania and Romania. In Lithuania, the absence of a dynamic national forest data management system means that spatial data are only updated once every decade. Thus, the monitoring and adjusting of forest plans is difficult to achieve.

Sixth, declines of old forest previously not subject to clear-felling and subsequent intensive management is common. In a steep forest history gradient in northern Sweden, Svensson et al. (2019) observed that the loss of forest area never subject to clear-felling and subsequent intensive forest management had occurred at a much higher rate than the establishment of additional protected areas.

Seventh, other land uses like mining occur locally, and wind power parks are frequently established in hilly areas, which so far usually have escaped transformation to intensive forest management due to their remoteness. This stresses the need for analyses of cumulative effects of multiple drivers.

The three positive factors, namely protective forests, buffer zones and inaccessibility due to poor transport infrastructures, were clearly associated to regions and countries of the former USSR (the two Russian case study areas Arkangelsk and Murmansk, and Ukraine and Lithuania) where such practices were mainstream during the Soviet period. However, buffer zones differ in terms of their aims (Naumov et al. 2017), and range from fulfilling protective functions such as hindering erosion, assisting in protecting the core area of strict protection, and carrying out management actions to suppress insect outbreaks. However, in Russia the 2007 Forest Code relaxed these regulations, which led to increased wood harvests in protective forests and riparian forests (Naumov et al. 2017). Habitat restoration attempts was a fourth positive factor.

**Fig. 6:**
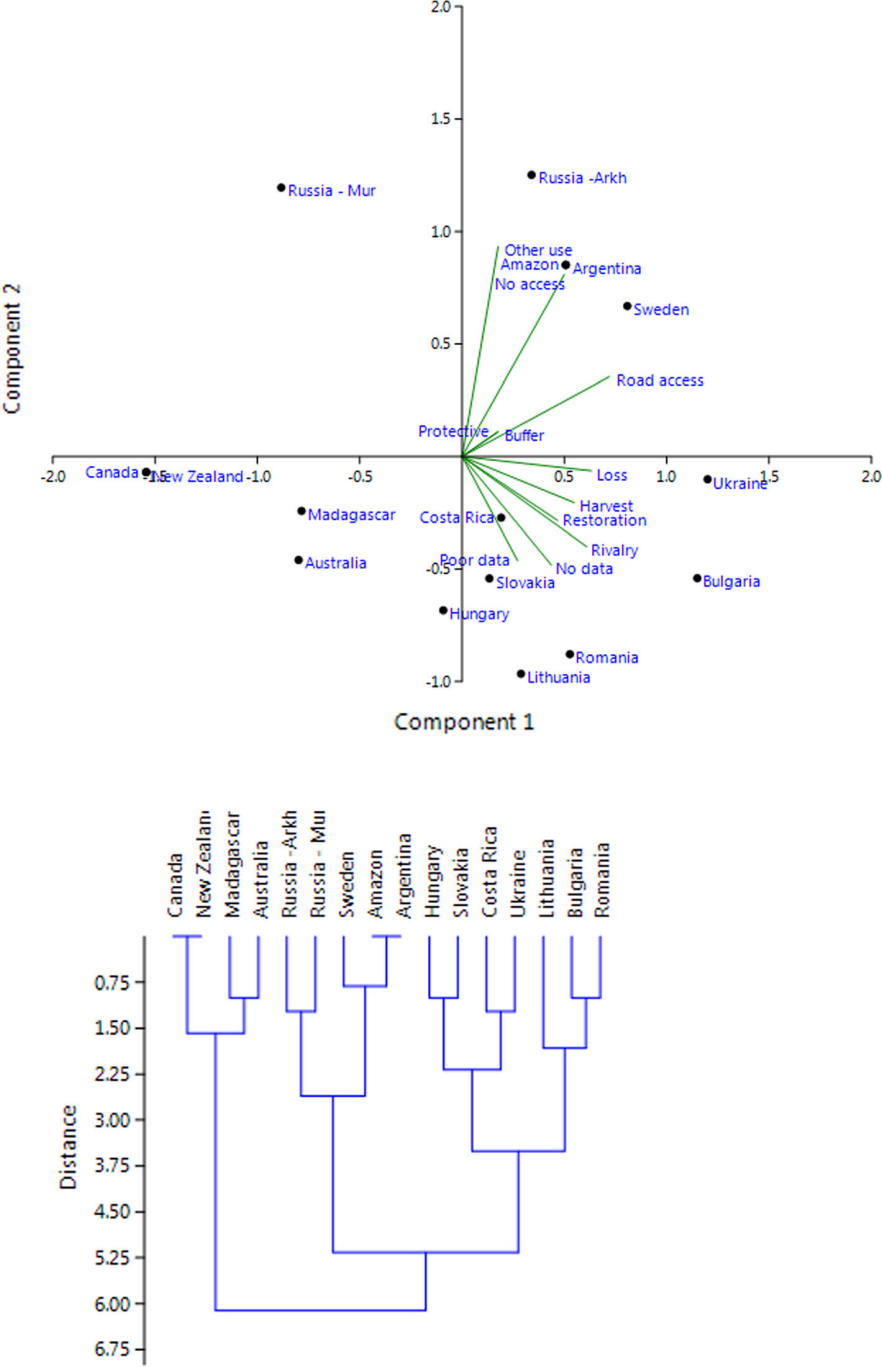
(Q4) PCA ordination and clustering based on variables in Table 7

An exploratory PCA ordination based on these 11 variables (Fig. 6) had an Eigenvalue of 0.75 for PC1, which explained 30% of the variance. Positive loadings included different kinds of negative effects from the matrix on protected areas and networks. PC2 included two variables representing accessibility, and other kinds of land use than forestry and agriculture. The Eigenvalue was 0.53 and explained an additional 21%. This resulted in three distinct clusters, viz.: (i) east European countries plus Costa Rica, (ii) areas with remaining large intact forest landscapes, and (iii) the case study areas in Canada, New Zealand, Madagascar and Australia.

## Discussion

### The “global forest environmental frontier” is in reverse

Transformation, fragmentation and loss of natural forest ecosystems have formed frontiers of expansion away from centers of economic development for millennia, and the process continues throughout the globe (e.g., Yaroshenko et al. 2001; Potapov et al. 2008; Margono et al. 2014; Angelstam et al. 2021a). For example, despite regional differences in losses of forest cover and efforts to halt them, commodity-driven deforestation rates have not declined since 2001 (Curtis et al. 2018), and the remaining wilderness areas are shrinking (e.g., Watson et al. 2018). To cope with the associated loss of species, habitats and natural processes that constitute biodiversity, protected areas of different kinds have been and are being created. This is a component of a global environmental policy frontier, with the ambition to design sufficient amounts and types of functional habitat networks. Such policies, such as CBD’s (2010) Aichi target #11 prescribe both quantitative targets such as 17% protected areas inspired by evidence-based knowledge (e.g., Svancara et al. 2005), but also qualitative targets addressing the functionality of protected areas and the networks they aim at forming, i.e., GIs. Currently higher target levels, including 30% protected areas (European Commission 2020; Secretariat of the Convention on Biological Diversity 2020), and Half Earth with a 50% target (Wilson 2016) are being proposed. At the global level, over the 2000–2020 period protected areas have increased numerically from 10 to 15% terrestrially, and from 3 to 7% in marine areas (Secretariat of the Convention on Biological Diversity 2020, p. 10 ff.). However, “progress has been more modest in ensuring that protected areas safeguard the most important areas for biodiversity, are ecologically representative, connected to one another as well as to the wider landscape and seascape and are equitably and effectively managed”.

This study is an attempt to conduct a transparent assessment of the net effect of the protected area versus forest exploitation frontiers in 16 case study areas on five continents. First, we mapped the portfolios and area proportions of protected area instruments aiming at forest biodiversity conservation. Second, inspired by CBD’s Aichi target # 11’s qualitative criteria, we explored ways to assess the effectiveness of different amounts of these set-aside categories. Third, we mapped the portfolios of policy implementation tools used for establishing protected areas and habitat networks. Fourth, we mapped negative and positive factors originating from the matrix surrounding protected areas. Therefore, focusing on the global environmental forest frontier theme of this Special Issue, is the net effect of protected area versus forest exploitation frontiers affecting habitat network functionally moving “forwards or backwards” on the ground?

The first question focused on the wide range of conservation instruments applied in different settings, and the proportions of formal forest protection (IUCN categories I to IV) and other measures. The variation was large, ranging from 3 to 77%. However, different countries had widely different portfolios of protected area and other set-aside categories aimed at conservation, sustainable use and protective functions. This means that adding different percentage points, without attempting to address the aims of different conservation instruments and their effectiveness is not meaningful.

The second question addressed the effectiveness of protected areas and resulting habitat networks. The case studies reported eight examples of factors that affect effectiveness of protected areas and resulting networks. These were ecological representativeness (1), habitat quality (2), functional connectivity (3), what kinds of resource extraction is allowed in protected areas (4), long time needed to deliver habitat by restoration (5), “paper parks” (6), “fortress conservation” (7), and lack of open access data about protected areas (8). That ecological representativeness is often poor is a general observation. This is linked to that protected areas are often created where competing land uses do not have any claims, which favors sites and regions with low biological productivity. While indicators of protected areas’ quality in terms of the level of naturalness are commonly more favorable than the surrounding matrix, nevertheless, evidence-based conservation targets for habitat quality and size may not be reached. Together, these factors affect structural and functional connectivity (e.g., Auffret et al. 2015), and thus the effectiveness of different conservation instruments in space and time as GI. Connectivity is commonly limited. This is partly due to that proportions of protected areas are low, and that spatial planning is not effective. For example, Ward et al. (2020) showed that, on average, globally only 11% of each country or territory’s protected areas can be considered as connected. Moreover, wood harvesting is allowed in a large part of the European forests protected for biological and landscape diversity. Verkerk et al. (2014) estimated that in Europe on average 52% of the volume can be felled in forests protected for biodiversity, and 60% in forests protected for landscape diversity. However, if the conservation vision is to maintain traditional cultural landscapes this can be warranted, as well as if forest landscape restoration aims at replacing conifers with deciduous trees (Angelstam et al. 2021a).

Criticisms of protected areas occur when they have little or no conservation impact (Paper Parks), or when protected areas conserve wild nature without respect to local communities’ values (Fortress Conservation). However, from the point of view of the traditional use of cultural landscapes these two concepts are not necessary counterpoles. Fortress Conservation can be viewed as a variant of Paper Parks when the role of maintaining biodiversity and cultural heritage for humans, as a component of social–ecological systems, is disregarded. Fortress Conservation is better known in relation to global south; however, signs of this concept appear in the EU and Russia. Finally, limited or lack of open access data about protected areas does not allow analyses of protected area categories’ spatial overlap, quality, size and spatial configuration, which is necessary to assess connectivity of protected area networks.

Credible evaluations of conservation instruments continue to be rare (Miteva et al. 2012). The third question therefore focused on the policy instruments, sensu Vedung (1998), that were applied to make policy work on the ground. Regulatory instruments dominated, and were followed by economic and informational tools. However, individual countries had different political cultures, and thus different portfolios of policy tools. A key next step would be to analyze the consequences on the ground of different policy instruments on habitat network functionality.

The fourth question addressed the portfolios of factors originating from the matrix surrounding protected areas, and which affect the functionality of individual protected areas and the efforts to maintain functional habitat networks (i.e., GIs). A total of seven negative and four positive factors situated in the matrix around protected areas were identified. Three positive factors (protective forests, buffer zones, inaccessibility) were exceptions, and were clearly associated to former USSR countries and regions with legacies of top-down regulation, and to Costa Rica. Finally, approaches to habitat and landscape restoration may be fragile given the increasing wood demand and low survival of retention trees (Rosenvald et al. 2019), but also promising for the future, if these regulations and other incentives aiming at restoration persist, because forest structures can change to more natural ones, albeit with long delivery time (Roberge et al. 2015; Crouzeilles et al. 2016).

The examples in this study indicate that the net effects of forestry intensification, matrix effects and expanding frontiers of transformation of natural and near-natural forest remnants on the one hand, and the environmental frontier’s encouragement of sufficient amounts of protected areas and functional habitat network on the ground on the other, were generally negative. This is in spite of gradually strengthened conservation policy (CBD 2020; EU 2021).with the aim to reduce threats to biodiversity through a net increase in area, connectivity and integrity and retaining existing intact areas and wilderness (IPBES 2019).

It should also be noted that traditional and indigenous land use can be of key importance in understanding and conserving a landscape’s biodiversity (e.g., Angelstam et al. 2021a). Throughout history, people have created and shaped today’s landscapes, for example fire stick farming in Australia (Jones 1969) and slash-and-burn farming combined with animal husbandry and multi-functional agriculture in boreal and temperate regions in the past, and still today in tropical regions like Madagascar and natural resources use by Māori in NZ (Lyver et al. 2019). Although forest harvesting was not part of the traditional hunter-gatherer economy, wood and non-wood forest products were important for daily life and human well-being (Feary 1988). Unfortunately, there is a focus on material values and forces that do not benefit or value the cultural traditions of indigenous people (Crush 1995). Nevertheless, indigenous peoples’ participation in forest landscape stewardship and management is slowly becoming recognized, being beneficial for resource management and for alleviating social and economic problems (Lewis and Sheppard 2005; Angelstam et al. 2021b). To conclude, this study re-iterates Watson’s et al. (2016) concern that there “is a real risk that Target 11 may be achieved in terms of area while failing the overall strategic goal for which it is established because the areas are poorly located, inadequately managed, or based on unjustifiable inclusion of OECMs” (i.e., other effective area-based conservation measures).

### Coping with “backwards” development of environmental frontier

#### Transitioning from “percent protected” to “green infrastructure”

Performance targets for biodiversity conservation are commonly expressed as proportions and ratios using percent as the quotient. Evidence-based conservation targets are typically 10–30% or more (e.g., Svancara et al. 2005; Betts et al. 2017), and which are then negotiated in policy processes, such as the 17% target of CBD’s (2010) Aichi target # 11. However, it is of paramount importance that both the area amount and category of “protected area” used to fulfill performance targets are defined (the dividend or numerator) as well as what it should be related to (the divisor or denominator). Because we focus on a particular type of land cover, forests, the denominator to estimate area proportions should in most cases not be the entire land area of an entire region or country, but of its forest area. The question is then if proportions should be expressed as:

1. the proportion of what once was forest (= all areas where forest was the potential natural vegetation), or
2. the proportion of what is the current forest cover?, and
3. what definition of forest or wooded land should be used; for example if only productive forest (e.g., wood growth rate > 1 m^3^ ha^−1^ year^−1^) should be considered or not.

Using Sweden as an example, different alternatives yield “protected” area proportions ranging from 8 to 31% (Angelstam et al. 2020a, b). However, protected areas can be successful or unsuccessful, effective or ineffective, and therefore there is no direct link between their area proportion and the state of biodiversity in an area. The ambiguity of numbers can be reduced if there is opportunity for assessing if protected areas and other conservation instruments, representing different forest types separately, form functional habitat networks or not. The Aichi target’s #11 qualitative indicators provide a comprehensive list of criteria that can be applied (e.g., Angelstam et al. 2020a, b), and different protected area categories can be attributed to for example IUCN’s (Dudley 2013) and Forest Europe’s (Duncker et al. 2012) classifications. This process requires insights about landscape history (Angelstam et al. 2021a), and whether conservation visions are based on natural or anthropogenic disturbance regimes (Kuuluvainen et al. 2021). Only then can different regions and countries be compared in a meaningful manner, rather than be driven by particular stakeholder interests in claiming high, or low, proportions of protected areas (Angelstam and Manton 2021).

For example, in the European Union area, only 3% of land and < 1% of marine areas are strictly protected. This does not necessarily mean the area is not accessible to humans, but that it should leave natural processes essentially undisturbed to respect the areas’ ecological requirements. To improve the situation the European Commission (2020) has put forward the target that at least 30% of the land and 30% of the sea should be protected in the EU, of which at least one third should be strictly protected. Thus it certainly matters if protected area proportions are expressed as the proportion of today’s forest, or of the amount of forest that was found naturally is the base for formulating conservation targets. Additionally, functionality needs to be addressed, which for example also depends on if the conservation vision is to maintain naturalness including a range of natural disturbance regimes (Kuuluvainen et al. 2021), or cultural landscapes maintained through tradition livelihood systems (Angelstam et al. 2021a, b).

#### Transparent knowledge about states and trends of green infrastructure functionality

A key aspect of assessing GI functionality is that appropriate and accessible data are available. Ambiguities of terms like “forest”, “forest cover”, “forest (canopy) loss” and “deforestation” illustrate this (Angelstam and Manton 2021). Remotely sensed the so-called “forest loss” data are widely used to assess aspects of forest conditions in time and space (e.g., Hansen et al. 2013). It is, however, critical to differ between deforestation and temporary canopy loss caused by wood harvesting and natural disturbances, and both can lead to counter-intuitive losses as well as gains of different aspects of biodiversity (Angelstam et al. 2021a). The spatial resolution used to identify loss of canopy has to be considered to assess if this is a result of final felling or of selective fellings in rotation forestry, or if it is management aimed at producing both wood and pasture. Indeed, in many regions throughout the world including Europe, Madagascar and northern Argentina, partial deforestation and canopy loss have led to multi-functional cultural landscapes based on integration of forest use, animal husbandry and agriculture, which resulted in bioculturally valuable silvopastural landscapes. On the other hand, even temporary canopy loss can reduce quality of forest habitat over repeated logging cycles. From a sustained yield wood production point of view, over entire forest rotations, forest canopy loss is temporary because forest canopy gain will maintain the same stand-age distribution. In spite of this, the chances of ever including old-growth forest processes, habitats and species are slim. Thus, while forest canopy loss does not mean the complete loss of forested area, the development of habitat characteristics typical for naturally dynamic forest including old-growth forest will not take place. The delivery time for such habitat characteristics is generally acknowledged to be 2–3 times longer than the length of common silvicultural cycles (e.g., Roberge et al. 2015). This means that with short rotations focusing on sustained yield of wood, the level of naturalness will remain low. However, given sufficient time, forest gain through afforestation and natural succession in abandoned agricultural areas and on non-forest land, as well as plantations, could lead to forest landscape restoration in the simplistic meaning of increased tree canopy cover without major effects on the composition, structure and function for effective biodiversity conservation. Detection of forest canopy loss is also scale dependent. For example, in Slovakia sanitary cutting of individual trees in a stand is responsible for 58% of the harvesting (Moravčík et al. 2019). This means that because of the absence of complete canopy loss, use of remote sensing data will not pick up this kind of habitat alteration. Combining spatial data for different types of habitat with evidence-based knowledge about species’ requirements regarding habitat quality and amounts as well as patch size and connectivity can support spatial planning (Manton and Angelstam 2018).

#### Reducing competition between material versus immaterial value chains

Forests provide multiple goods, services and values. Typically, expanding frontiers transforming naturally dynamic forest landscapes have focused on producing contributions to human well-being and welfare. One widespread focus is on deforestation to produce food and feed with different intensities. Another focus is on big trees and wood in general, which have led to expanding timber frontiers, and subsequent loss of intact forest landscapes, and in some regions to intensive forest management focusing on high sustained yield forestry. However, the importance for rural development of agriculture and industrial forestry has declined dramatically due to urbanization, as well as mechanization and merging of wood-based mills to large units. For example, forests beyond the timber frontier in Sweden and Russia are currently also seen as beneficial for developing local jobs based on new value chains supporting rural development which are based on nature, wilderness and culture, thus more than on wood and wood-based products (Jonsson et al. 2019; Angelstam et al. 2020a, 2020b). In Russia’s Arkhangelsk region protected areas can contribute to the well-being of the local communities and provide livelihoods by developing the use of non-timber forest products and ecotourism (Mikhailova and Efimov 2015). Similarly, indigenous people in the Amazon and New Zealand want their ecoregion or land they own to be protected for such multiple use reasons. Different countries and regions thus have different views on the role of different value chains. For example, in Slovakia some stakeholders argue that protected areas have negative socio-economic impacts in terms of job losses and lower revenues in wood-processing (Kovalčík et al. 2018). On the contrary, rural areas in NW Russia and Costa Rica enjoy considerable tourism benefits. A key issue is who benefits from this. In the past local coffee plantations provided jobs, and benefits remained in the country. Investments in tourism and conservation benefits are based on foreign capital, and thus lead to benefits outside the country. This inequality has increased.

To conclude, there is competition between different forest value chains. Two different avenues to cope with this competition are payments for what protected areas deliver, thus reducing competition between competing value chains, and spatial planning of landscapes and regions (e.g., Ward et al. 2020). The former is illustrated by the case studies Argentina and Costa Rica. The latter is exemplified by the Soviet zoning legacies with remnants in Lithuania, Romania, Russia, and Ukraine, through Argentina’s traffic light approach narratives, and Nova Scotia’s triad approach (for details, see Appendix S1). To encourage development of marginalized immaterial value chains, such as based on non-wood ecosystem services, efforts to strengthen social capitals supporting landscape stewardship is crucial (Angelstam et al. 2021b).

#### Landscape approaches to foster knowledge production and learning

The Amazon Biome and the EU versus post-Soviet border zone between Romania and Ukraine represent two examples of efforts to handle complex protected area contexts. While an Amazon Biome-wide cross-national integration of protected areas is progressing, major challenges remain. Regional and local co-management and co-governance of protected areas is indispensable but still a distant goal because governments and their agencies continue to dominate both. The management of transboundary protected areas suffers from incompatible legal frameworks among countries and federative states in Brazil that need a profound revision to achieve common goals. Additionally, it is unclear how to continuously guarantee the funds needed by governments to properly manage protected areas, and what can be done to satisfy and effectively control the myriads of economic actors interested in land and resources to be protected. Biodiversity conservation is combined with declared sustainable development goals that actually include generating benefits to local residents, but also income to meet the economic needs of protected areas to reduce the economic burden on the state. This results in management that endangers biodiversity conservation goals and local livelihoods.

In Europe both so-called Natura 2000 sites in the EU and Emerald sites outside the EU aim to protect biodiversity (Opermanis et al. 2012). However, the potential for coordinated conservation efforts would benefit from better defined obligations in protected areas located in neighboring states being parts of different policy contexts (e.g., Dallimer and Strange 2015; Sotirov et al. 2015; Winkel et al. 2015; Blicharska et al. 2020). While spatial functional connectivity of cross-border protected areas can be assessed, the influence of environmental and political factors should also be taken into consideration (Ilieş et al. 2012; Opermanis et al. 2012). The natural areas in the East Carpathian Mountain range along the Romanian–Ukrainian border are a good example. This includes the Maramureş Mountains National Park and the Rodna Mountains National Park on the Romanian side and by the Carpathian Biosphere Reserve in Ukraine. The first steps to unify these protected areas into one cross-border conservation-oriented territory were implemented in 2007, and in 2009 the Collaboration Agreement that created the Romanian–Ukrainian Cross Border Biosphere Reserve Maramureşului Mountains was signed (Ilieş et al. 2010). This area maintains high levels of naturalness, and has “a remarkable unused tourism potential” (Ilieş et al. 2012). However, in this transborder area, functional and structural EU versus Post-Soviet land use and governance legacies meet. Thus, lack of harmonized regional strategies, poor infrastructure and services, limited management capacity and participation in international partnerships (Ilieş et al. 2012) may hinder the development of coordinated cross-border conservation projects.

These examples from the case study areas illustrate the need for regionally adapted area and place-based landscape approaches (e.g., Arts et al. 2017). Critically important conditions for developing place-based knowledge production and learning representing different social–ecological contexts include: (1) sufficient time for developing collaborative capacity as an iterative process, and (2) production of knowledge about states and trends of ecological and social systems involving both quantitative and qualitative methods (e.g., Lyver et al. 2019). This implies transdisciplinarity built on coordination among academic disciplines and non-academic participants. The critical need of having committed persons as visionaries, project leaders, and holders of knowledge and key project competences to champion a process is well documented (e.g., Dawson et al. 2017).

Finally, this review highlights the similarities among case study areas in terms of the long-term development of expanding frontiers of forest alteration, fragmentation and loss to secure material benefits in terms of wood and fuel, human food and animal feed. For example, during the period 1980–2000, 55% the new agricultural land in the tropics came from deforestation of intact forests, and 28% from altered forests (Gibbs et al. 2010). This replicates what took place thousands of years ago in Old World temperate forest landscapes (Thomas 1956; Williams 2003). Similarly, without reducing the forest cover, the development of effective sustained yield wood production has reduced the amounts of natural forest structures far below critical tipping points. We also observed interesting broad-scale patterns among case study areas, such as the commonalities in policy instruments and portfolios of driving factors typical for post-Soviet legacies, and different phases of transformation of forest landscapes. Sustaining efforts to implement evidence-based conservation targets in terms of functional habitat networks through landscape planning remains an urgent task.

## Conclusions

In spite of half a century of policy development to maintain biodiversity through protected areas, conservation management, and landscape restoration, the negative net effects of frontiers of protected areas versus forest exploitation on species, their habitats, and ecosystem functions caused by forest exploitation do remain. Insights from the 16 case study countries and regions across 5 continents globally demonstrate a wide range of drivers of decline. To tackle these problems, we propose (1) transitions from discussing only percent protected areas to also estimating the contributions from different conservation instruments and the surrounding matrix to ecologically representative functional GIs, (2) producing and using transparent knowledge about states and trends of GI functionality, (3) reducing competition between forest value chains based on material forest benefits such as wood versus values as traditional multiple use landscapes, wilderness, and biodiversity, and (4) secure continuous collaborative learning by implementing landscape approaches adapted to social–ecological and cultural contexts. Multiple case studies like this are useful for comparisons of different policy instruments and their consequences and require inter and transdisciplinary approaches that can provide both evidence-based knowledge about states and trends and effective forest governance through regionally adapted solutions.

## Supplementary Information

Below is the link to the electronic supplementary material.

Supplementary file1 (PDF 6493 kb)
