# Supplementary material for: Frontiers of protected areas versus forest exploitation: Assessing habitat network functionality in 16 case study regions globally
Source: Ambio. 2021 Oct 17;50(12):2286–310. doi: 10.1007/s13280-021-01628-5 (PMC8563882; doi:10.1007/s13280-021-01628-5)
Supplement: Supplementary file 1 — Supplementary file1 (PDF 6493 kb) [file 13280_2021_1628_MOESM1_ESM.pdf]

2021-09-03
Ambio
Electronic Supplementary Material, not peer reviewed

Frontiers of protected areas versus forest exploitation: assessing habitat network functionality in 16 case study regions globally

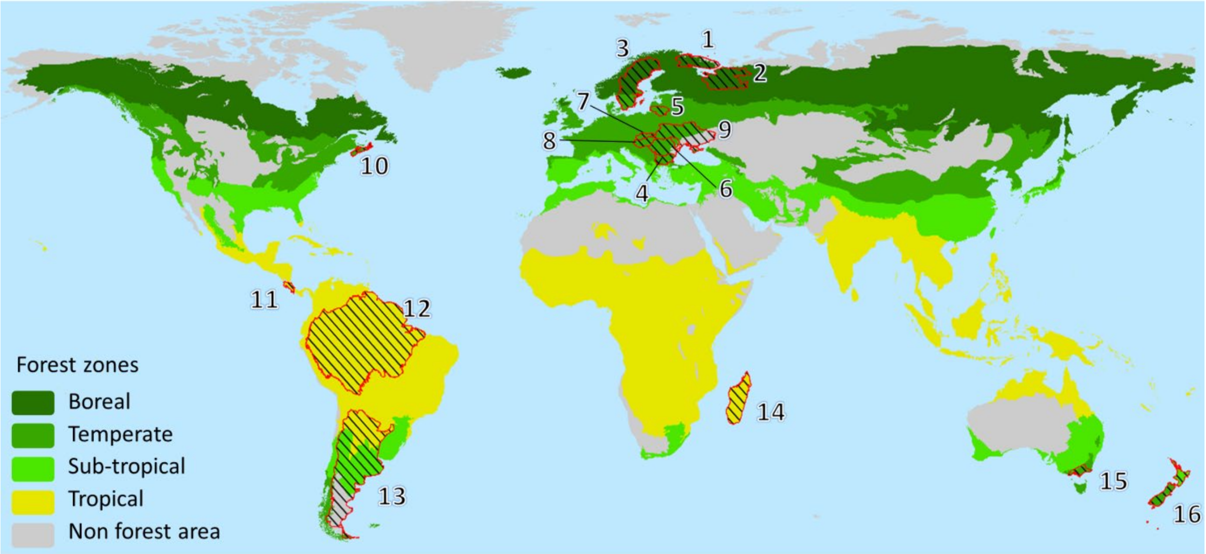

Map showing the location of the 16 case study areas, and where forests and woodlands form the potential natural vegetation.

List of contents

Bulgaria (4) — **5**
- Case study description — 5
- Portfolios of conservation instruments and area proportions (question 1) — 5
- Effectiveness (question 2) — 5
- Policy instrument tools (question 3) — 5
- Net effects of conservation and intensification (question 4) — 6
- References — 7

Hungary (8) — **8**
- Case study description — 8
- Portfolios of conservation instruments and area proportions (question 1) — 8
- Effectiveness (question 2) — 9
- Policy implementation tools (question 3) — 10
- Net effect of conservation and intensification (question 4) — 11
- References — 11

Lithuania (5) — **13**
- Case study description — 13
- Portfolios of conservation instruments and area proportions (question 1) — 13
- Effectiveness (question 2) — 13
- Policy implementation tools (question 3) — 13
- Net effect of conservation and intensification (question 4) — 14
- References — 17

Romania (6) — **18**
- Case study description — 18
- Portfolios of conservation instruments and area proportions (question 1) — 19
- Effectiveness (question 2) — 22
- Policy implementation tools (question 3) — 22
- Net effect of conservation and intensification (question 4) — 23
- References — 24

Russia – Arkhangelsk oblast (2) — **27**
- Case study description — 27
- Portfolios of conservation instruments and area proportions (question 1) — 27
- Effectiveness (question 2) — 27
- Policy implementation tools (question 3) — 29
- Net effect of conservation and intensification (question 4) — 31
- References — 32

Russia – Murmansk oblast (1) — **35**
- Case study description — 35
- Portfolios of conservation instruments and area proportions (question 1) — 37
- Effectiveness (question 2) — 38
- Policy implementation tools (question 3) — 41
- Net effect of conservation and intensification (question 4) — 41
- References — 41

Slovak Republic (7) — **43**
- Case study description — 43
- Portfolios of conservation instruments and area proportions (question 1) — 43
- Effectiveness (question 2) — 44
- Policy implementation tools (question 3) — 44
- Net effect of conservation and intensification (question 4) — 45
- References — 46

Sweden (3) — **48**
- Case study description — 48

Portfolios of conservation instruments and area proportions (question 1) 48
Effectiveness (question 2) 48
Policy implementation tools (question 3) 48
Net effect of conservation and intensification (question4) 49

Ukraine (9) Fel! Bokmärket är inte definierat.
Case study description **Fel! Bokmärket är inte definierat.**
Portfolios of conservation instruments and area proportions (question 1) **Fel! Bokmärket är inte definierat.**
Effectiveness (question 2) **Fel! Bokmärket är inte definierat.**
Policy implementation tools (question 3) **Fel! Bokmärket är inte definierat.**
Net effect of conservation and intensification (question 4) **Fel! Bokmärket är inte definierat.**
References **Fel! Bokmärket är inte definierat.**

Nova Scotia (10) 59
Case study description 59
Portfolios of conservation instruments and area proportions (question 1) 59
Effectiveness (question 2) 61
Policy implementation tools (question 3) 61
Net effect of conservation and intensification (question 4) 62
Works Cited: 62

Costa Rica (11) 63
Case study description 63
Portfolios of conservation instruments and area proportions (question 1) 63
Effectiveness (question 2) 63
Policy implementation tools (question 3) 63
Net effect of conservation and intensification (question 4) 64
References 66

The Amazon biome (12) 68
Case study description 68
Portfolios of conservation instruments and area proportions (question 1) 69
Effectiveness of protected areas (question 2) 69
Policy implementation tools (question 3) 70
Net effect of conservation and intensification (question 4) 71
References 72

Argentina (13) 74
Case study description 74
Portfolios of conservation instruments and area proportions (question 1) 74
Effectiveness (question 2) 76
Policy implementation tools (question 3) 76
Net effect of conservation and intensification (question 4) 77
References 79

Madagascar (14) 81
Case study description 81
Portfolios of conservation instruments and area proportions (question 1) 81
Effectiveness (question 2) 82
Policy implementation tools (question 3) 84
Net effect of conservation and intensification (question 4) 84
References 85

Australia's Mountain Ash and Alpine Ash trees in Victoria (15) 87
Case study description 87
Portfolios of conservation instruments and area proportions (question 1) 87
Effectiveness (question 2) 87
Policy implementation tools (question 3) 88
Net effect of conservation and intensification (question 4) 88

References 89

New Zealand (16) 91

Case study description 91

Portfolios of conservation instruments and area proportions (question 1) 91

Effectiveness (question 2) 93

Policy implementation tools (question 3) 93

Net effect of conservation and intensification (question 4) 94

References 95

Bulgaria (4)
By Tzvetan Zlatanov

Case study description
Bulgaria has territories in three out of the 10 Natura 2000 biogeographical regions of Europe (EEA 2019). These are the Alpine, the Continental and the Black Sea regions. Not only the character of the forest vegetation changes from one biogeographical region to another, due to variation of climate and soil conditions, but also main conservation and management issues are regionally specific. With climate change, forest growth conditions are getting better at higher elevations, e.g. in the Alpine region (Zlatanov 2017), while in some of the lowest elevated localities in the Black Sea and the Continental regions annual precipitation is projected to drop below the minimum threshold that supports growth of forest tree vegetation in the near future (Raev 2010).

Portfolios of conservation instruments and area proportions (question 1)
Until the 2010^{s} only formally protected forested areas had been declared in Bulgaria. Those are under the jurisdiction of the Ministry of Environment and Water and include forest reserves, characterized by the most strict protection status, national parks and protected sites (altogether 206 900 ha or 5.64% of Bulgaria's total forested area). Additionally, 92,000 hectares (2.51%) in the Natura 2000 network have been set aside as the so-called "Forests designated to old-growth transformation" in 2016 (EFA 2016). These forests remain under the jurisdiction of the Ministry of Agriculture and Foods and largely incorporate the forests of the high conservation value, which are set-aside under a voluntary forest certification program for temporary protection. Altogether, "Formally protected forests" and "Forests designated to old-growth transformation" sums to 298 000 ha, which is 8.15% of the total forested area in Bulgaria. Including also unproductive forests, a total of 11.24% of the total forest fund is under certain kind of protection (Table BG1). The forested area in strict forest reserves, however, is only 65 700 ha, which is 1.79% of Bulgaria's total forested area.

Effectiveness (question 2)
The proportions of the total forest fund of different kinds of conservation instruments are quite unbalanced across different biogeographical regions. The alpine region is best represented with 12.0% of the formally protected forests while this proportion is only 1.97% in the continental region (Table BG2). The forested area in strict reserves is not only insufficient at a country scale (only 1.8%) but it is also quite unevenly distributed across the biogeographical regions with the alpine region incorporating more than 75% of these forests. the connectivity in the Continental and the Black Sea regions is not high. Additionally, few of the main tree species are sufficiently well represented in the formally protected forested sites (e.g., *Fagus sylvatica, Picea abies and Pinus nigra*). All distinctive three species for the lower vegetation belt (up to 1000–1200 m. a.s.l.), such as *Quercus* sp., *Fraxinus* sp., *Populus* sp., *Salix* sp. are quite inadequately represented across the protected sites. The spatial distribution and species composition of "Forests designated to old-growth transformation" is more balanced but these are forests that have just recently been allocated as set-asides, and few stands have achieved a biological maturity (mostly *F. sylvatica* and *P. abies*).

Policy instrument tools (question 3)
"Carrot": Various forest certification programs, especially FSC, guarantee access to desirable markets and stimulate owners to set aside forested areas, according to the requirements of the certification mechanisms. Additionally, compensatory payments for lost profits in the Natura 2000 network have a great potential. In Bulgaria this mechanism does not work effectively in

the forest sector at present but the framework for next funding period is being currently developed and there is optimism.

"Stick": The number of policy instruments to deal with forest conservation and management has increased substantially during recent decades. This has happened mainly as a result of the introduction of Natura 2000 in Bulgaria. A number of forest management regulations for the territories in the Natura 2000 network have been introduced in the Forest law and in the consecutive orders of its implementation. As such, these regulations refer to more than 55% of the total forest fund in the country. Unfortunately, they have proven to be rather ineffective as in reality forest management has not changed and do not differ from the management outside Natura 2000 network. Additionally, came the order of the Minister of Agriculture and Foods to discontinue active management in 10% of the areas of each forest habitat in the Natura 2000 network.

"Sermon": Education campaigns to enlighten the society and forest managers of the principles of Natura 2000 network seems to have been the most commonly used instrument. Annual trainings are being implemented to improve forest managers' capacity in nature conservation and restoration management. However, efficiency of these courses need to be increased.

Net effects of conservation and intensification (question 4)

As a result of the social and policy changes in the country, the forest administration has tripled since the 1990s. Meanwhile, at the same time the annual harvest rate doubled – from approximately 4.5 mill m^3 in 1990 to nearly 9 mill m^3 at present (EFA 1990–2018). Under these circumstances, a there is a pronounced societal divide between the desire to harvest more and a wish for more conservation areas and less intense forestry. The pressure rise due to lack of trustful information of the amount of wood annual increment, especially under projected climate change conditions (Bulgaria is the only "forested" country in EU without a National Forest Inventory in permanent sample plots). Unfortunately, the various new regulations have not resulted in a more balanced forest planning and management approach. For example, coppice forests at lower altitudes that have been subject to mass conversion activities to seed originated ones for the last 30 years are still coppiced, in younger ages and with deteriorated quality for biodiversity. Here there are also pronounced deficiencies in protected forested areas. In the mountain region there are attempts to introduce uneven-aged silvicultural systems with preservation of some old-growth elements (e.g., deadwood and biotope trees) in "Forests designated to old-growth transformation". However, this has not given effect on stand structures yet, naturally, and the approach is quite fragile given the increasing wood demand. In fact, it is quite promising for the future, as well as it is the voluntary act to designate "Forests of high conservation value" under the certification process. If these regulations persist, then forest structures will gradually change to more natural ones, but it will take decades. Under abovementioned circumstances, the lack of newly announced formally protected forests during the last decade, especially in the Continental biogeographical region of the country (which is the greatest in area), can be considered as one of the main failures of the Ministry of Environment and Water policy. This is especially accentuated by the shear fact that forest management in the Natura 2000 network has not brought added environmental protection benefits yet and is not likely to bring such in the near future.

What can be learned about the "global environmental frontier"? It is getting increasingly easier to negatively influence the wilderness areas "beyond" global environmental frontier. In

the past, it was lack of technologies to guarantee protection of forests in remote or difficult to cultivate areas. Today with much more advanced machinery and better road infrastructure, the society needs to take legislative measures to effectively protect the fewer remaining wild areas – remote or around us. Effective protection is important as there is a risk for the society to be misled by the many conservation instruments, some existing only on paper.

Table BG1. Proportion of all forest conservation instruments in Bulgaria, and their effectiveness.

Spatial unit (Total forested area)	Conservation instruments (% of all forested land by spatial unit)				
	Formally protected forests	Forests designated to old-growth transformation (including voluntary set-asides)	Unproductive forests	Tree retention on clear-cuts	SUM
Bulgaria/ (3.67 mill ha)	5.64	2.51	3.09	NA	11.24
Continental ecoregion/ (2.186 mill ha)	1.97	2.08	3.05	NA	7.10
Alpine ecoregion/ (1.19 mill ha)	12.05	3.17	3.20	NA	18.42
Black See ecoregion/ (0.291 mill ha)	7.01	3.02	2.90	NA	12.93

References

European Environment Agency: https://ec.europa.eu/environment/nature/natura2000/biogeog_regions/index_en.htm. Accessed on 28.03.2019.

EFA. 1990–2018. Executive Forest Agency, Ministry of Agriculture and Foods, Forms Forest Fund. http://www.iag.bg/docs/lang/1/cat/13/index. Last accessed on 28.03.2019.

EFA. 2016. Executive forest agency. Ministry of Agriculture and Foods, Order 93-10549/02.11.2016. http://www.iag.bg/docs/lang/1/cat/14/index. Accessed on 28.03.2019.

Raev, I. (Ed.). 2010. Program for forest adaptation measures for mitigation of the negative impact of climate change in Republic of Bulgaria (incl. maps). Bulgarian Executive Forest Agency, Sofia, 32 pp.

Zlatanov, T., Elkin, C., Irauschek, F. and Lexer, M.J. 2017. Impact of climate change on vulnerability of forests and ecosystem service supply in Western Rhodopes Mountains. Regional Environmental Change 17: 79–91. https://doi.org/10.1007/s10113-015-0869-z.

Hungary (8)

By Réka Aszalós

Case study description

Currently, forests (20,560 km^2) cover 22.1% of Hungary's total area (93,030 km^2). Considerable afforestation efforts on agricultural land over the past century mean that forest cover has increased from about 11,000 km^2 until after ca. 1950 (Halász 1994; National Forestry Database 2012[1]; Tobisch and Kottek 2013). Most forests belong to the Pannonian mixed ecoregion. Forest stands with the highest level of naturalness and associated biodiversity (i.e. oak, oak-hornbeam and beech forests) cover the hilly and mountainous regions of the country, and represent one third of the forest habitats (7% of the country). Arable fields and intensive, short-rotation plantations of non-native tree species usually hosting low level of biological diversity constitute about half of the forest land (www.globalforestwatch.org), and dominate the lowland areas. The dominant silvicultural system in Hungary is even-aged rotation, mainly clearcutting systems (71%) and uniform shelterwood systems (20%). The area proportion of uneven-aged management (i.e. selection systems) is 1%, and forests representing the transition from even-aged rotation forestry to uneven-aged silvicultural system cover 4%, and in the remaining 4% of "forests with no commercial purposes" regular timber extraction is not allowed. A privatization process in the 1990s created about 400,000 forest estates, with an average area of 2.2 hectares. A total of 42% of Hungary's forests has private owners, or no owner. (http://www.cepf-eu.org/page/hungary).

Portfolios of conservation instruments and area proportions (question 1)

Nationally protected areas and partly over-lapping EU nominations (Natura 2000), as well as set-asides in managed forests and tree retention are the four conservation instruments in Hungary (Table HU1).

Table HU 1. Conservation instruments and estimated of the proportion of forest land. Note that due to overlaps between national and EU level conservation instruments numbers cannot be added.

	Nationally protected areas (%)	Natura 2000 (%)	Forests with no commercial purposes (%)	Tree retention (%)
Hungary (20.560 km^2)	22, of which 3.5 percentage points is specially protected	40 (of which half overlaps with national protection)	4	1

First, 22% of the forests are protected by national legislation (Law of Nature Conservation) (approx. 4,160 km^2), and out of that 3.5 percentage points is specially protected (730 km^2). Second, the Natura 2000 system derived from EU policy for biodiversity conservation is responsible for the protection of 40% (8,430 km^2) of forest habitats. Practically all natural and semi-natural forests under Natura 2000 protection, half of which overlaps with protection according to the national Nature Conservation Law. The third instrument of forest protection

[1] https://www.mecsekerdo.hu/_user/browser/File/pdf/Efol/EFOL_Forestry%20in%20Hungary.pdf
https://portal.nebih.gov.hu/documents/10182/862096/Forestry_related_databases.pdf/3ff92716-2301-4894-a724-72fafca9d4fc

is based on the Forestry Law of Hungary, and it's regulations for implementation, which define the function and management (including restrictions) of all forest stands. Regular timber extraction is prohibited in 4% (770 km^2) of the forests ("forests with no commercial purposes"), often due to special protective functions, such as soil and water protection, or on account of nature conservation value. The majority of these forests are state owned, in which timber extraction is prohibited or restricted due to their protection function. The network of 63 protected forest reserves have core areas which are left for free development – their total area is 40 km^2 (i.e. 5.5% of the total area of these specially protected areas, which is 730 km^2, see Table Hu2) (Mázsa et al. 2013, www.erdorezervatum.hu).

The Forest Law of Hungary incorporated several conservation measures of the Natura2000 National Regulation in order to meet the expectation of Natura 2000 National Regulations. The fourth conservation instrument that nature conservation authorities can initiate is green tree retention up to 5% and 5 m^3 of deadwood retention. Temporal or spatial restrictions due to the breeding/presence of a species considered to be of European Community interest (https://www.eea.europa.eu/data-and-maps/indicators/species-of-european-interest-2/assessment) are also important measures, for which private owners are compensated for their potential losses. However, there is no compensation in state-owned forests.

Effectiveness (question 2)

The formal forest protection measures are not effective for forest biodiversity conservation. The reason is that approximately 75% of the forest stands under national protection are managed by shelterwood systems, or other intensive silvicultural systems. Neither does strict protection guarantee the absence of commercial forest management; in fact >40% of these stands are managed with rotation forestry (Table HU2). Hungarian law bans regular timber extraction only in 4% of all forests, which corresponds to the 12% of the nationally protected forests (management type: "forests with no commercial purposes"). However, sanitary cutting is allowed. These "set-asides", even if not exclusively biodiversity-oriented, are one of the most effective tools of biodiversity protection. As a result of abandonment of management they often show old-growth characteristics, like high amount of deadwood, with diverse log size categories and decay stages (Aszalós et al. 2017; Bölöni et al. 2017). The core areas of forest reserves have old-growth characteristics, but they represent only the 0.2% of the forested area (ca. 40 km^2). The majority of the protected and specially protected forests, forest reserves and stands with this limitation on commercial timber extraction ("forests with no commercial purposes") can be found in the more natural hilly and mountainous part of the country.

Table HU 2. Silvicultural systems used in different protection categories of Hungarian forests.

	specially protected forest (730 km^2)	Protected forest (3,850 km^2)	not protected forest (16,003 km^2)
regular timber extraction prohibited	28.6%	9.5%	1.5%
uneven-aged management	8.6%	3.1%	0.3%
transition to uneven-aged management	14.8%	9.0%	2.0%
rotation forestry (shelterwood and clearcutting systems)	48%	78.4%	96.2%
Sum	100%	100%	100%

Old-growth forests of Hungary are generally threatened. Forests >120 years, which are crucial to nature conservation, covers only two percent (370 km^2) of the country's forest area (Hungarian National Forest Stand Database), and one-third of these forests is not protected at all. At least the half of forests located on protected and specially protected public lands are continuously exploited (Gálhidy 2016a, 2016b).

Selection management systems, like group selection maintain uneven-aged diverse forest stands, and host higher levels of forest-adapted species diversity than even-aged stands (Elek et al. 2018, Boros et al. 2019). However, their relative share is currently very low (1%). To deal with this challenge, new regulations of the Ministry of Agriculture encourage the adaptation of all continuous-cover-forestry methods. Nature conservation authorities can suggest and initiate green tree and deadwood retention as well as the conservation of large ancient tree individuals or habitat trees, but there is no strict regulation on these actions. As far as it remains only "suggestion", the adaptation of these techniques depends only on the nature conservation attitude of the given forester, and private land owner.

Policy implementation tools (question 3)

"Carrot": Compensation supporting for forest owners' and users' lost profits in the Natura 2000 network have a great potential. In Hungary this mechanism was began in 2012, but does not work effectively in the forest sector. However, there are some good examples, like compensations for temporal or spatial restriction due to the breeding/presence of a species of European Community interest (https://www.eea.europa.eu/data-and-maps/indicators/species-of-european-interest-2/assessment). The concept of the framework for next funding period is being currently developed, using all the positive and negative examples of the last period to develop a more effective compensatory system. Four forest companies out of the 22 has FSC certification.

"Stick": Introduction of the EU's Natura 2000 system, as a new instrument of nature protection, was supposed to strengthen and complement the national protection systems. However, regarding forest ecosystems this instrument does not prove to be very effective. Natura 2000 forests with national protection are managed almost in the same manner as protected forest without Natura 2000 designation, and Natura 2000 forest stands without national protection are managed with similar intensity, as any other not protected habitats. Accordingly, 73% of the Natura 2000 forests with national protection are managed with rotation forestry, which is 90% in case of nationally not-protected Natura 2000 forests (Table HU3).

"Sermon": EU-funded LIFE projects, like LIFEinForests, have held several campaigns and trainings for authority specialists, licensed forestry engineers, and national park experts. The focus has been to improve their knowledge on alternative forestry techniques, importance of forest microhabitats, deadwood, ancient large trees, for preserving biodiversity in Natura 2000 forests (https://en.lifeinforests.eu/), i.e. the practices and authority tools that can help maintaining and improving the conservation status, and to inform forest managers about EU funds for Natura 2000 sites. There are other frameworks of awareness rising to educate forest practitioners, however, the efficiency of these trainings is not proven.

Table HU3. Silvicultural systems used in different combination of national protection and Natura2000 categories of Hungarian forests.

	Protected, and Natura 2000 (4,160 km^2)	Protected, not Natura 2000, (420 km^2)	Not protected by national law, and Natura 2000, 4,180 km^2)	not protected by national law, and not Natura 2000, , (11,830 km^2)
regular timber extraction prohibited	13.2%	6.1%	4.0%	0.7%
uneven-aged management	4.3%	1.1%	0.4%	0.2%
transition to uneven-aged management	9.9%	10.5%	5.0%	1.0%
rotation forestry (shelterwood and clearcutting systems)	72.7%	82.3%	90.5%	98.1%

Net effect of conservation and intensification (question 4)

Nationally protected areas, as well as the Natura2000 sites, are in general managed for wood production by forestry companies. The proportion of set-asides with free development is extremely low. The absolute guarantee for no timber extraction is only the core area of the forest reserves (ca. 40 km^2), representing only the 0.2% of the forested area. The area of old-growth forests (age>120 years) are slowly, but continuously decreasing. Only about half of the area of Natura 2000 sites under national protection or strict protection has a high level naturalness. The population size of different game species is extremely high, (National Game Management Database, www.vvt.gau.hu), causing severe damage on forest regeneration and loss of forest biodiversity. On the other hand, because continuous cover forestry is promoted by the Ministry of Agriculture, significant changes are expected in the proportion of uneven-aged forests (now it is only 1%). The long-term data of the National Biodiversity Monitoring System (www.nbmr.hu) does not show any rapid decrease in the abundance and species richness of forest dwelling animals and plants. Thus, nevertheless, a slow decrease of biodiversity can be predicted if the management, protection policy and implementation of nature-conservation regulations of the Hungarian forests will not be changed.

References

Aszalós, R., Horváth, F., Mázsa, K., Ódor, P., Lengyel, A., Kovács, G. and Bölöni, J. 2017. First signs of old-growth structure and composition of an oak forest after four decades of abandonment. *Biologia* 72(11):1264-1274.

Bölöni, J., Ódor, P., Ádám, R., Keeton, W. S. and Aszalós, R. 2017) Quantity and dynamics of dead wood in managed and unmanaged dry-mesic oak forests in the Hungarian Carpathians. *Forest ecology and management* 399:120-131.

Boros, G., Kovács, B. and Ódor, P. 2019. Green tree retention enhances negative short-term effects of clear-cutting on enchytraeid assemblages in a temperate forest. *Applied soil ecology* 136:106-115.

Elek, Z., Kovács, B., Aszalós, R., Boros, G., Samu, F., Tinya, F. and Ódor, P. 2018. Taxon-specific responses to different forestry treatments in a temperate forest. *Scientific reports* 8(1):1-10.

Gálhidy, L. 2016a. Magyarország legidősebb erdei. WWF Magyarország (The oldest forests of Hungary. WWF Hungary)

Gálhidy, L. 2016b. Old-growth forests in Hungary are in danger; https://wwf.panda.org/?269991/Old-growth-forests-in-Hungary-in-danger
Mázsa, K., Balázs, B., Bölöni, J. and Horváth, F. 2013. Strict Forest Reserve Research in the Margin of the Carpathians, the Vár-hegy Case-Study. In *The Carpathians: Integrating Nature and Society Towards Sustainability* (pp. 257-269). Springer, Berlin, Heidelberg.
National Game Management Database, www.vvt.gau.hu
Tobisch, T. and Kottek, P. 2013. Forestry-related databases of the Hungarian forestry directorate. *National Food Chain Safety Office (NFCSO), Web site, Hungary*. https://portal.nebih.gov.hu/documents/10182/862096/Forestry_related_databases.pdf/3ff92716-2301-4894-a724-72fafca9d4fc

Lithuania (5)

By Michael Manton

Case study description

Lithuania is situated in the European hemi-boreal mixed broadleaved-coniferous forest ecoregion, which is the transitional zone between the boreal coniferous forests and the nemoral broadleaved forests (Bohn et al. 2003; Karazija 1988). Forests cover 2.19 M ha, this equates to 33.5 % of Lithuania. The tree species are coniferous (55.5 %), deciduous softwood (40.9 %) and deciduous hardwood (3.5 %) (Lithuanian State Forest Service 2017).

Portfolios of conservation instruments and area proportions (question 1)

After regaining independence in 1990, Lithuania developed policy that segregated forests into different forest management function groups. This built upon the approach of forest zoning developed through the Soviet era (Brukas 2015; Brukas et al. 2013; Naumov et al. 2017). According to the Forest law of the Republic of Lithuania (2010), forests are divided into four groups with pre-defined objectives of economic activities, their management regime and major functional purpose. The segregation includes the following groups i) strict nature reserves; ii) which is divided into (A) ecosystem protection, and (B) recreational; iii) protective; and iv) commercial. Each of these groups are associated to a set of defined harvesting regulations (Table LT1). This is the primary tool used for forest conservation purposes. However, other voluntary tools are utilised to conserve Lithuanian forest landscapes include internationally initiated initiatives representing concepts likes Ramsar, Biosphere Reserve, forest certification schemes, woodland key habitats and Natura 2000.

Effectiveness (question 2)

Restitution of forest land since independence in 1990 plays an important role in understanding Lithuania's forest harvesting and conservation effectiveness. During the past 30 years wood production has increased from approximately 3 million m^3 to 7.2million m^3. This increase coincides with the increase in private forest ownership (from 0 to 40 %) with harvesting going from 0 to 3.8 million m^3/yr in 2014 (Lithuanian State Forest Service 2017). In addition, state forest harvesting has also increased from 3 million m^3 in 1990 to 5.3 million m^{3}in 2014 and this trend continues (Lithuanian State Forest Service 2017).

The conservation and protection of Lithuania's forests is undertaken by the functional group outlined above. This means that protected area system differs greatly from the IUCN's protected area network and can thus be misleading (Belova et al 2005; Lazdinis 2011). For instance, national parks in Lithuania are not strictly protected areas and forest harvesting is permitted. For instance, within the 48638 ha of forest of the Dzukija National Park in southern Lithuania only 1950 ha is strictly protected (group I) (Lithuanian State Forest Service 2017). This indeed leads to many ongoing questions about the effectiveness of Lithuania's use and reporting of conservational instruments and their area proportions. In addition, it is common for an area to be allocated under the protection of multiple protection types (see previous section).

Policy implementation tools (question 3)

"Carrot": Various forest certification programs have been implemented. Currently, all Lithuania's state forests are certified using the FSC standard, thus covering approximately 50% of all Lithuanian forests. The woodland key habitat program is also voluntary. However, it is not as actively used as when it was initiated due to the lack of funding. Indeed, many of the FSC set-asides and woodland key habitat areas overlap (Elbakidze et al. 2016). The EU Habitats and Birds Directives form the basis for Lithuania's Natura 2000 areas. However, the

process to incorporate Natura 2000 into Lithuanian policy has only recently commenced, and is thus still not legalized but still has great potential.

“Stick”: The number of policy instruments to deal with forest conservation and management has increased with Lithuania becoming an EU member in 2004. Natura 2000 based on the EU Habitats and Birds Directives is becoming an important regulating mechanism. Forest certification has become a normal activity. However, forest management has not changed dramatically as Lithuania’s forest law are stricter than the general certification criteria applied. A new FSC certification standard was implemented in 2021 that increased voluntary forest protection from 5 to 10%. The woodland key habitat program is also voluntary; however, due to lack of funding and weak policy inclusion its future is unsecure. Indeed, many of the FSC set-a-sides and woodland key habitat areas overlap (Elbakidze et al. 2016). The overlap of multiple protection area initiatives on a particular forest area has given land managers many uncertainties in both applying and meeting required National and International management actions for the maintenance of forest in protected areas.

“Sermon”: Education campaigns to enlighten the society and forest managers on the principles of nature conservation and Natura 2000 network seems to a positive instrument, although there is much confusion in its implementation. Regional and National Parks provide education programs, but have very limited impact on forestry as their forests do not fall under their own custodianship, but belongs to the forest department. Other initiatives include short-term projects. Forest certification also provides educational knowledge for forest owners and managers.

Net effect of conservation and intensification (question 4)

According to the national forest inventory database (2017) 30.8% of Lithuania’s forest area is protected (Table LT2). On closer inspection only 1.2 % of forests are strictly protected (forest function group I (strict nature reserves)), this figure also includes non-productive wetland forest. Group II forest (A) ecosystem protection forests & (B) recreational forests) with reduced protection status and stricter harvesting restriction make up 11.8 % of forest. These two groups are considered as highly protected areas by most foresters. These two area protection figures have remained constant since 2000. The forest area under voluntary protection in Group iii and iv has increased from 0 % to a combined 17.6 % since 2000 with the implementation of the EU Habitats Directive and Birds directive, FSC certification, and woodland key habitats. Although forest harvesting was conservative in the 1990s (e.g., Brukas 2015) a gradual transition to increased forest management intensification can be seen from the early 2000s (Lithuanian State Forest Service 2020). However, strict forest protection has remained stable and the increases in voluntary protection will not maintain or secure Lithuania forest biodiversity for the future (Elbakidze et al. 2016).

Table LT1. Key Forest harvesting protection instruments

	Legislation and year	Definition/Purpose	Objective of economic activities and restrictions
**Group I** (State Forest Strict reserves)	Article 16 of the Law of the Republic of Lithuania on Forests 2010	These include state reserves, national and regional park reserves, and reserve roundabouts. Forests belonging to this group are left to grow naturally without human intervention.	No forestry or silviculture is permitted except for special cases defined under the Law on Protected Areas and the Regulations of strict nature reserves.
**Group II**			
A (ecosystem protection forests)		Include landscape, telmologic, pedologic, botanical, forest genetic, zoological, botanical-zoological reserves and reserves of these types in national parks and biosphere monitoring territories, forests with protected natural resource areas, anti-erosion and other forests.	To preserve or restore forest ecosystems or separate ecosystem components. Forests damaged by the natural events, forest stands of poor sanitary condition shall be cut by non-clear or clear sanitary cuttings. Forest stands can be harvested upon reaching natural maturity by final by applying selective harvesting Harvest ages varies from of 51 to 201 depending on tree species.
B (recreational forests)		Include forest parks, urban (city) forests, forests of recreation zones of state parks, recreational forest areas and other forests defined for recreation.	To form and preserve a recreational forest environment. Forest stands damaged by the natural events and forest stands of poor sanitary condition shall be cut by non-clear or clear sanitary cutting methods. Forest stands of natural maturity can be cut by using selective harvesting from the age of 31 to 141 depending on tree species. All types of forest thinning, sanitary and landscape formation cuttings are allowed. Harvesting stands affected by natural events should be harvested after the recreation season
**Group III** (protective forests)		These are the forests in the territories of geological, geomorfological, hidrographical, and cultural reserves, reserves of these types in the state parks and biosphere monitoring territories, forests of protection zones and other forests.	To form productive forest stands , capable of performing the functions of protection of soils, air, water and human living surroundings. Selective harvesting and clear cuts <5 ha as well as forest tending, and sanitary harvesting are permitted.<br>Final forest harvesting age varies from 31 to 141 depending on tree species
**Group IV** (economic or commercial		These are the forests not ascribed to I-III groups.	To form productive forest stands and supply wood continuously following environment protection requirement. All forest harvesting methods are permitted. Clear cutting areas shall not exceed 8 ha. Final forest harvesting age varies from 31 to 121 depending on tree species
**Further important forest harvesting restrictions**			
Bird Nest	Article 16 of the Law of the Republic of Lithuania on Forests 2010	Nesting sites of birds under protection shall be protected by designating breeding sites within a specified radius. Information on protected bird nesting sites is registered and published in the Protected Species Information System and published in the State Forest.	No forest harvesting within 50-200m of the nest tree, depending on bird species.<br>In addition, harvesting maybe prohibited from 1 April until September 1.
Retention trees		To preserve forest biodiversity in forest groups II-IV.	Stands >1 ha: leave at least 7 live trees per/ha (of which at least 3 are older or thicker than average trees in the stand) and at least 3 dead trees over 20 cm at DBH per/ha<br>Stands <1 ha: leave at least 3 live trees and at least 2 dead trees with a diameter of >20 cm. Retention tree should be important for biodiversity in and left for decay.
**Voluntary Protection Instruments**			
**Forest Certification**	FSC 2020	FSC forest certification requirements for forest operations in Lithuania, including small and low-intensity managed forests.	Forest certified by the Forest Stewardship Council (FSC) uses a national standard. Under this standard, the minimum voluntary set-aside area is 10% of the forested area.
**Woodland Key Habitats**	Not applicable	WKH is an intact forest area with a high probability of a present non-accidental occurrence of an endangered, vulnerable, rare or care-demanding habitat specialist species (Andersson 2005).	Management restrictions are suggested but not enforced due to their voluntary nature.

**BAST**	**EU Habitats Directive**	to protect biological diversity in the EU by creating a network of protected areas. The aim of the Habitats Directive is to maintain, and if needed, restore natural habitats, wild flora and fauna of community importance for favourable conservation status (European Commission 2015).	
**PAST**	**EU Bird Directive**	Birds Directive also requires the restoration of past and the creation of new biotypes necessary for birds (European Commission 2015).	
**Natura 2000**	**EU Habitats Directive & EU Bird Directive**	Europe-wide network of protected areas consisting of territories important for the conservation of birds and habitats, whose purpose is to protect, maintain and if needed, recreate natural habitat types, animal and plant species (European Commission 2015).	

Table LT2. Distribution of forest land area by forest groups (Lithuanian State Forest Service 2017; 2020).

	2003	2016	2019
	Area ha (%)	Area ha (%)	Area ha (%)
Group I	23 929 (1.2)	26 541 (1.2)	25 337 (1.2)
Group II	243 592 (11.9)	266 481 (12.2)	260 335 (11.8)
Group IIa*	178 609	201 116	199 480
Group IIb*	64 982	65 365	60 856
Group III	325 889 (15.9)	333 419 (15.2)	288 156 (13.1)
Group IV	1 451 877 (71.0)	1 560 305 (71.4)	1 623 289 (73.9)
Sum	2 045 287	2186 746	2 197 117

* Indicates the division between type a and b Group II forests

References

Andersson L., Kriukelis R. and Skuja S. 2005. Woodland Key Habitat inventory in Lithuania. Vilnius, Lithuania.

Belova, O., Karazija, S., and Šaudytė, S. 2005. Country report - Lithuania (pp. 211-231). In COST Action E27. Protected Forest Areas in Europe-analysis and harmonisation (PROFOR): Reports of Signatory States. Editors: Frank, G., Parviainen, J., Vandekerhove, K., Latham, J., Schuck, A., & Little, D. Federal Research and Training Centre for Forests, Natural Hazards and Landscape (BFW).Vienna.

Bohn, U., Neuhäusl, R., Gollub, G., Hettwer, C., Neuhäuslová, Z., Raus, Th., Schlüter, H. and Weber, H. 2003. Map of the Natural Vegetation of Europe. Scale 1:2 500 000. Münster (Landwirtschaftsverlag)

Brukas, V. 2015. New World, Old Ideas—A Narrative of the Lithuanian Forestry Transition. Journal of Environmental Policy & Planning 17(4):495-515. doi: 10.1080/1523908X.2014.993023

Brukas, V., Felton, A., Lindbladh, M. and Sallnäs, O. 2013. Linking forest management, policy and biodiversity indicators–A comparison of Lithuania and Southern Sweden. Forest ecology and management 291:181-189.

European Commission. 2015. Report from the commission to the council and the European parliament. The State of Nature in the European Union Report on the status of and trends for habitat types and species covered by the Birds and Habitats Directives for the 2007-2012 period as required under Article 17 of the Habitats Directive and Article 12 of the Birds Directive. Brussels.

Elbakidze, M., Ražauskaite, R., Manton, M. Angelstam, P., Mozgeris, G., Brumelis, G., Brazaitis, G. and Vogt, P. 2016. The role of forest certification for biodiversity conservation: Lithuania as a case study. European Journal of Forest Research 135 (2):361-376.

FSC (Forest Stewardship Council). 2020. The FSC national forest stewardship standard of Lithuania. Page 74. Forest Stewardship Council, Bonn, Germany.

Karazija, S. 1988. Lietuvo miškų tipai [Forest types of Lithuania]. Mokslas, Vilnius (In Lithuanian).

Lazdinis, I. 2011. Implementation of International Requirements for Protected Areas in Lithuanian Forestry. Legal Aspects of European Forest Sustainable Development, Proceedings of the 13th International Symposium. IUFRO. Kaunas.

Lithuanian State Forest Service. 2017. Lithuanian statistical yearbook of forestry 2017. Lutute, Vilnius, Lithuania.

Lithuanian State Forest Service. 2020. Lithuanian Statistical Yearbook of Forestry 2019. Ministry of Environment, State Forest Service, Vilnius, Lithuania.

Seimas of the Republic of Lithuania. 2010. On the Approval of Regulations for Forest Fellings: The Order of the Minister of the Environment of the Republic of Lithuania No. D1-79 of 27 January 2010. In: Environmental Protection Ministry, Valstybės žinios, Vilnius, Lithuania.

Naumov, V., Angelstam, P., Elbakidze, M. 2017. Satisfying rival objectives in forestry in the Komi Republic: Effects of Russian zoning policy change on forestry intensification and riparian forest conservation. Canadian Journal of Forest Research 47:1339–1349. https://doi.org/10.1139/cjfr-2016-0516

Romania (6)

By Andra-Cosmina Albulescu

Case study description

The Romanian forestry law defines the forest fund as comprising forest areas, tree plantations, wooded pastures, river beds, ponds and other land covers in forest landscapes. (Forest Act 2015). Out of the 65,650 km^2 total forest fund of Romania, 64,060 km^2 are forest areas, of which approximately 30,108 km^2 are represented by forests used for wood production (MWF 2017; types V and VI, Table RO1). With a total area of 238,397 km^2 Romania's forest cover is thus 27%.

The Ministry of Waters and Forests in Romania defines six biogeographical regions (Alpine, Continental, Pannonian, Pontic, Steppe and the Black Sea), all of which include forest vegetation of different types, mostly broad-leaved, except the Black Sea Biogeographical Region with non-forest and non-terrestrial biotopes (MWF 2015). According to the EEA (2009), the forest ecological regions that extend over Romania's territory are the Carpathian montane (Alpine) coniferous forests, the Pannonian mixed forests and the Central European mixed forests. The largest biogeographical region that contains forest area is the Alpine one in the Carpathian Mountains encompassing 49.8% of the forest cover of Romania, while the least extended biogeographical regions are the Pontic and Pannonian, with less than 1% of the forest cover each (Fig. RO1).

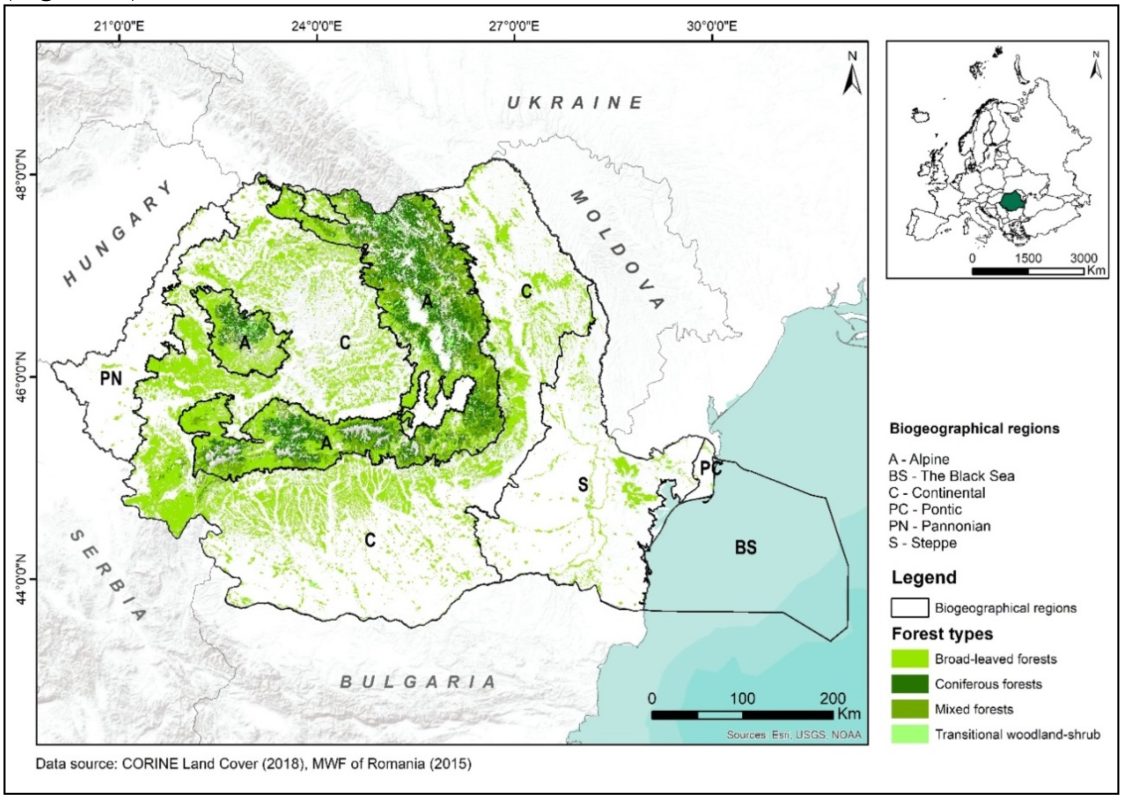

Figure RO1. The biogeographical regions of Romania and the forest types

Portfolios of conservation instruments and area proportions (question 1)

The Romanian forestry related laws serve as conservation instruments, the most prominent being the Forest Code ("Codul Silvic") from 1996, and was changed several times over the years. It was elaborated in order to regulate forest exploitation and conservation practices, as a legislative instrument representative for the new democratic regime.

Productive forests are exploited according to a forest management plan drafted for ten years, and following a tract timber inventory that must be completed one year before harvesting (Forest Code 2015). Complementarily, protected forests are usually part of national parks and reserves, which are monitored by rangers that have to ensure that they are not illegally exploited. Also, the law requires that owners of private forests have to hire rangers to manage their properties. State Forest Inspectorates have been fulfilling functions related to forest inspections and law enforcement in public and private forests since 1999, while the National Forest Administration Romsilva administrates protected forestlands, manages state owned forests and ensures the supply of wood for the state's economy (Ioraș and Abrudan 2006, Lawrence 2009).

The Forest Code (2015) defines two functional groups of forests, Group I being considered formally protected, and Group II partially protected and mainly destined to production purposes. There are not any formally defined voluntary protected forest areas, such as under forest certification schemes, nor unproductive forests and tree retention areas. Group I forests (57.3% of the forest fund) include forests that should perform ecological services related to hydrological networks, climate, soil and hunting aspects and also protect national objectives of interest, forests in national parks and forests destined to recreational activities. There are two types. Type I consists of formally protected forests that are strictly protected from exploitations, and where interventions require the approval of the Romanian Academy. Type II can be classified as voluntarily protected forests, because only conservation works are permitted there. Group II forests (42.7% of the forest fund) are considered productive forests, and to a lesser extent forests that should perform protection functions like the ones in the first group (Forest Code 2015, MWF 2017). There are four types. Type III and IV have quite an uncertain status given by the definition that "intensive regeneration treatments are permitted, with the exception of spruce, pine, poplar, acacia and willow for which clear-cuts may be applied" (MWF 2017). Type V and VI are subjected to all kinds of treatments and may fit into the category of productive forests covering 47% of the forested part of the forest fund. The proportions of the forest areas in Table 1 is equivocal, and the Romanian authorities need to develop better instruments for forest monitoring and data gathering in order to provide more accurate results.

Table RO1. Forest types groups and types in Romania and their proportions.

Type of forest		% of forest area	Forest area (km^2)	Designated categories
Group I	Type I	3	1921	Formal protection
	Type II	21	13452	Voluntary protection
Group II	Type III	8	5124	Uncertain
	Type IV	21	13452	Uncertain
	Type V	5	3203	Productive forest
	Type VI	42	26905	Productive forest

Romania's entrance into the EU in 2007 marked significant changes of the Romanian forestry sector regarding conservation (Manolache et al. 2017). A new conservation instrument consists of the so called Natura 2000 sites, which compose a network of protected areas that are also breeding and resting sites for different species and constitute valuable habitats (EC 2020). The network builds on two EU directives: (i) the Habitat Directive which designates Special Areas of Conservation (SACs) based on a list of Sites of Community Importance (SCIs) and (ii) the Birds Directive that defines Special Protection Areas (SPAs) (Council Directive 92/43/EEC 1992, Directive 2009/147/EC 2009, Evans 2012).

The implementation of the new network of Natura 2000 sites took place in stages. Initially, designation of Natura 2000 sites was considered to hinder economic development by some local communities that overlooked the benefits of conservation (Manolache et al. 2017). Previous studies emphasise that considering this network a social one, and not recognizing its ecological character, diminishes its potential and counteracts its prerogatives (Maiorano et al. 2007, Young et al. 2007).

In 2019, across all ecoregions, there were 606 Natura 2000 nominations in Romania (435 SCIs and 171 SPAs), totalling 606 areas covering 60,577 km^2 (EC 2019), i.e. 24.4% of Romania's 238,397 km^2. Of these, 587 Natura 200 sites contain forestland, including 420 SCIs that contain forest areas (over 24,504 km^2) and 167 SPAs that encompass this type of vegetation (over 17,289 km^2) (Table RO2). The overlap between the protected areas network established prior to Natura 2000 sites and the new SCIs and SPAs reaches 96%. However, some of the SCI sites actually overlap to some extent with the SPAs, which means that total area and proportions are difficult to compute accurately (see Figure RO2).

Additionally, because spatial data regarding the forest groups or types are not available, computations concerning the distribution of protected area areas among forest groups in different forest biogeographical units are not possible. However, Natura 2000 sites GIS data and CORINE Land Cover 2018 data may be used to compute the extent of the forestland in the protected areas in the biogeographical units in Romania (Figure RO2, Table RO2).

Almost 86% of the SCI areas with forest in Romania are located in the Continental and Alpine biogeographical regions, due to their domination. On the other hand, the Pontic biogeographical region is represented by a single SCI that overlaps with a SPA (Biosphere Reserve of the Danube Delta). The Steppe biogeographical region also encompasses a significant forest area, with 40 SCIs (over 17,777km^2), whereas the forestland in the Pannonian biogeographical region sums up to almost 194 km^2.

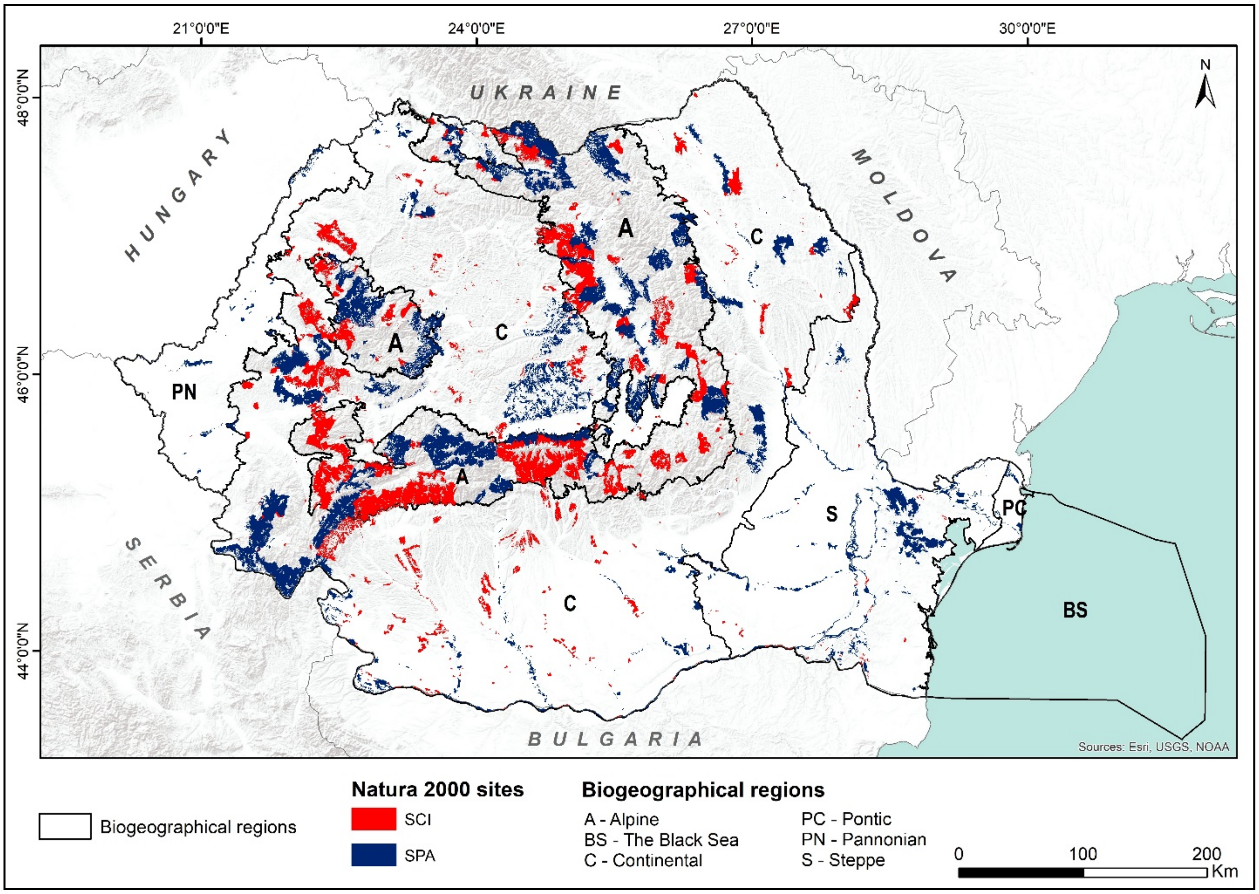

Figure RO 2. The forest vegetation in Natura 2000 sites by biogeographical region

Table RO2. Proportion of the protected forest areas included in the types of Natura 2000 sites (SCI and SPA), by biogeographical region (Romania). Note that because the protected area categories SCI and SPA do overlap somewhat, they cannot be summed.

Unit (area in km^2)	SCIs	SCIs area (%)	SPAs	SPAs area (%)
Alpine (50,072.6 km^2)	124	14093.17 (28.1)	36	8553.45
Continental (134,699.8km^2)	237	8372.28 (6.2)	80	6533.99
Pannonian* (14,001.1km^2)	18	193.97 (1.4)	10	229.47
Pontic** (2,389.8km^2)	1	67.82 (2.8)	1	67.82
Steppe*** (37,212.6km^2)	40	1777.64 (4.8)	40	1904.99
Total (238,376.2km^2)	420	24504.90 (10.3)	167	17289.75

Sum of * ** *** is 53,605 km^2 (3.8%)

Effectiveness (question 2)

Lacking proper quantitative data concerning the evolution of forest exploitation practices in the biogeographical regions of Romania, the task of assessing the contribution of the forest conservation instruments to the goal of achieving the CBD Aichi target #11 is difficult. Previous studies focused on evaluating the forest area preservation (Albulescu and Larion 2019) or forest disturbances (Munteanu et al. 2016) at county level or in protected areas (Knorn et al. 2012). Nonetheless, the forestry sector in Romania is known for a problem that helps to emphasise not necessarily the particularities of the effectiveness of the aforementioned instruments, but rather an alarming trend of forestland mismanagement (Greenpeace Reports 2009-2017, Lawrence 2009; Bouriaud and Marzano 2012,;Albulescu and Larion 2019). For example, Greenpeace Reports (2009-2017) and numerous other studies show that the forest areas in Romania are affected by consistent illegal logging (Bouriaud and Marzano 2012; Niță 2015; Albulescu and Larion 2019). This means that forests are exploited without permission from the authorities, the exact extent of this practice being still poorly quantified. The latest Greenpeace report shows that illegal forest cutting is on the rise, the number of reported cases being 32% higher in 2017 compared to 2016. The counties that are most affected by this issue in 2009-2017 are Argeș, Bacău and Gorj located in the Continental biogeographical region and Mureș and Prahova in the Alpine one (Greenpeace Romania 2017)

Recent enquiries conducted by Agent Green have shown that even forests in protected areas are subject to outlawed fellings. The Călimani, Domogled-Valea Cernei and Semenic-Cheile Carașului national parks are located in the Alpine biogeographical region, and stand out among the protected forest areas where illegal forest cuttings happen (Agent Green 2020). The same NGO informs that since 2014, a total of 1.2 million m^3 of wood have been harvested in the national parks of the country, although the forestry law strictly forbids this. Other sources that back up this information are ClientEarth and EuroNatur organisations.

Policy implementation tools (question 3)

Carrot: In 2014, Romanian authorities complied to the EU requirements of implementing a system that would help monitor wood harvesting, transports and commerce (SUMAL), but did not maintain the system functional by failing to pay for the remote sensing imagery that served as a validation base for forest exploitation. Since 2018, organisations concerned with forest conservation, together with Romanian citizens have been asking the government to properly implement the SUMAL system, and also the Forest Inspector, a smartphone application that allowed citizens to check the legality of wood transport and that needed updates. Without these technological instruments, illegal logging cannot be properly monitored and sanctioned (Agent Green 2020).

Stick: At the beginning of 2020, The European Commission announced that it may take legal action against Romanian authorities for failing to protect its valuable natural forests and conducting harvesting operations in Natura 2000 sites. By undertaking such exploitations without properly assessing their impact on the environment, the Romanian forestry system failed to meet the expectation of EU legislation. Also, an infringement launched by the European Commission sanctions regarding the inadequate implementation of the EU Timber Regulation was made in the endeavour to stop the entrance of illegally obtained timber on the EU market (ClientEarth 2020).

Sermon: There are multiple voices that ask the authorities to increase the transparency of the management process (Manolache et al. 2017), and to improve the overly prescriptive forest legislation (Drăgoi et al. 2011; Nichiforel et al. 2018; Popa et al. 2019). The infringement imposed by the European Commission indicates that the establishment of Natura 2000 sites in 2007, and the progressive inclusion of valuable habitats in 2011 and 2016, have not been effective. In fact, the expansion of the Natura 2000 sites' list led to tensions and misunderstanding between factors that influence the evolution of the forest management system, namely the Romanian Government, public authorities, landowners and other stakeholders, NGOs and research institutes (Manolache et al. 2017). Another issue that raise concerns is that during the COVID-19 crisis the number of illegal forest exploitation cases surged, a trend favoured by the fact that authorities are busy managing the health-related emergencies (Greenpeace Romania 2020). This situation also calls for immediate action by updating the SUMAL forest monitoring system.

Net effect of conservation and intensification (question 4)

In a context marked by forest inventory data scarcity, the net effect of conservation is hard to assess and only some tangential inferences may be drawn out. The most important issues that preclude proper conservation in Romania are: weak conservation-law enforcement, natural disturbances, unsustainable levels of wood harvesting, poaching, pollution and protected areas design errors (Primack et al. 2008).

Iojă et al. (2010) stated that a decade ago, the network of protected areas accounted for 19.2% of Romania's territory, while in 2005 it was of 7.1% and in 1989, at the beginning of the new political regime, this percentage was 4.1%. Today, Natura 2000 sites of SCI type represent 10.2% of Romania's total area, while the SPA sites cover 7.2% of it.

The overlap between the protected areas network already established prior to joining and adopting the Natura 2000 system in terms of new SCIs and SPAs reaches 96.19%, meaning that the introduction of Natura 2000 has by and large been redundant (Iojă et al. 2010). In some cases, the management and conservation goals are contradictory, which in turn generates tensions and the need to prioritize (Primack et al. 2008; Wilson et al. 2009; Ioraș et al. 2010). Still, the new protected area network does not solve the problem of the former one concerning the uneven representation of the ecoregions (Ioraș et al. 2010).

On the other hand, National Institute of Statistics data (NIS 2018) show that the volume of legally harvested wood has been increasing since 2009, with a few years of decline (Fig. RO3). This means that production forests have been intensely exploited, often via the clearcutting technique; the high harvesting pace removing the positive effect of an increase in the protected areas percentage.

Also, the inclusion of more and more territories on the protected area list leads to a series of negative consequences of social and economic nature (Sutherland et al. 2009). This becomes even more relevant in places where the Natura 2000 sites engulfed private properties and the former owners were not offered alternatives to compensate for the economic loss produced by the transformation of their lands into conservation-oriented areas (Pierce et al. 2005; Paavola et al. 2009; Ioraș et al. 2010).

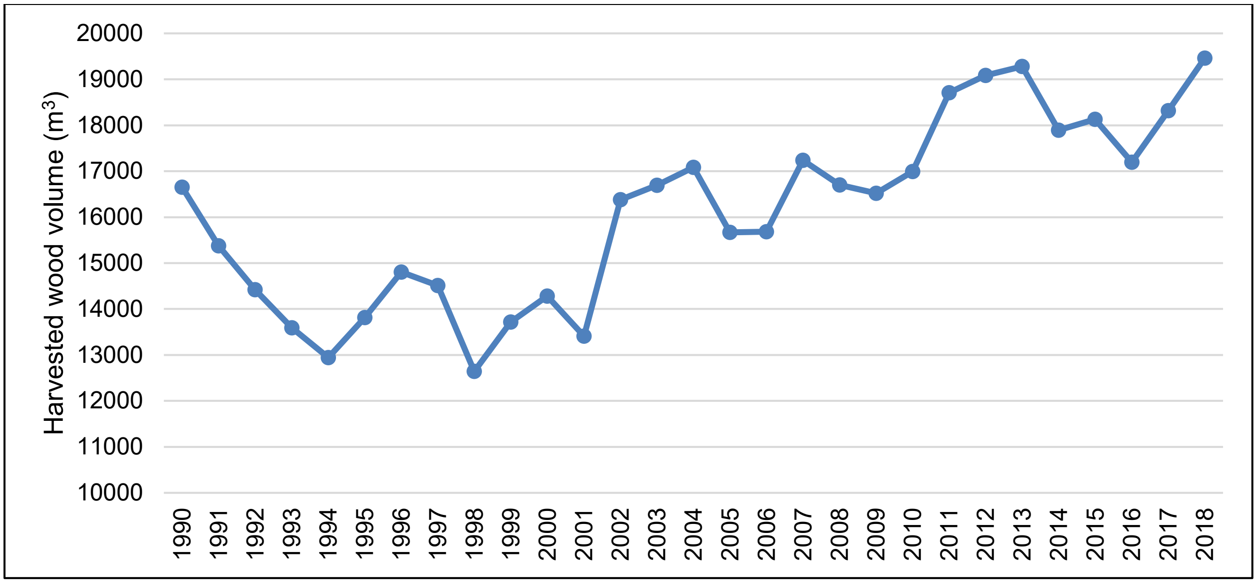

Figure RO 3. The evolution of harvested wood volume in Romania, 1990-2018

Previous studies highlight that the overall effectiveness of the protected area network in Romania has decreased since the creation of Natura 2000 sites, although the authorities aimed for the opposite outcome. The main issues identified relate to the lack of spatial prioritization, the weak involvement of local communities in conservation planning, the underrepresentation of plants and invertebrates, the status of under-staffed administrative organisms (Iojă et al. 2010) and the marginal involvement of NGOs, local enterprises, research institutes in the context of public administration dominance (Manolache et al. 2018). Moreover, it is hard to diminish the gap between the management and conservation effectiveness of Natura 2000 sites in Romania and the ones in Western European states, considering the underfunding and inadequate conduct of the institutions that should ensure proper conservation practices (Cogălniceanu and Cogălniceanu 2010).

References

Agent Green (2020). Dimensions of outlaw forest exploitation in national parks, retrieved from https://www.agentgreen.ro/exclusiv-agent-green-publica-dimensiunea-exploatarilor-criminale-din-parcurile-nationale/ on the 23rd of April 2020

Albulescu, A. C. and Larion, D. 2019. Application of fuzzy and classical Multi-Criteria Decision-Making methods in assessing the forest area preservation level of Romania's counties. *Baltic Forestry 25*(2):263-272.

Bouriaud, L. and Marzano, M. 2012. Conservation, extraction and corruption: will sustainable forest management be possible in Romania. In Gilberthorpe, E. (ed.), Natural resource extraction and indigenous livelihoods: development challenges in an era of globalisation, Ashgate International, 300pp.

ClientEarth. 2020. Media release, retrieved from https://www.clientearth.org/press/eu-announces-legal-action-against-romania-for-illegal-logging-of-europes-last-natural-forests/ on the 23rd of April 2020

Cogălnicanu, D. and Coglălniceanu, G.C. 2010. An enlarged European Union challenges priority settings in conservation. Biodiversity and Conservation 19:1471-1483.

Corine Land Cover (CLC). 2018. Version 20, retrieved from https://land.copernicus.eu/pan-european/corine-land-cover/clc2018?tab=download at the 25th of March 2020

Council Directive 92/43/EEC. 1992. Council Directive 92/43/EEC of 21 May 1992 on the conservation of natural habitats and of wild fauna and flora. Official Journal L 206:7–50.

Directive 2009/147/EC. 2009. Directive 2009/147/EC of the European Parliament and of the Council of 30 November 2009 on the conservation of wild birds. Official Journal L 20:7–25.

Drăgoi, M., Popa, B. and Blujdea, V. 2011. Improving communication among stakeholders through ex-post transactional analysis – case study of Romanian forestry. *Forest Policy Economics* 13: 16–23.

EC (European Commission). 2019. Natura 2000 Barometer statistics, retrieved from https://www.eea.europa.eu/themes/biodiversity/document-library/natura-2000/natura-2000-network-statistics/natura-2000-barometer-statistics/statistics/barometer-statistics on the 22nd of April 2020

EC (European Commision). 2020. Natura 2000 sites, retrieved on the 22nd of April 2020 from https://ec.europa.eu/environment/nature/natura2000/index_en.htm

EEA (European Environment Agency). 2009. Digital Map of European Ecological Regions, retrieved from https://www.eea.europa.eu/data-and-maps/figures/dmeer-digital-map-of-european-ecological-regions on 25th of March 2020

Evans, D. 2012. Building the European Union's Natura 2000 network. Nature Conservation: 1:11–26.

Forest Act. 2015. Republished Law no. 46/2008 on the Forest Code (Legea nr. 46/2008 Codul silvic). *Monitorul Oficial*, nr. 611/12.08.2015, Available at http://legislatie.just.ro/Public/DetaliiDocument/170527, accessed on the 10th of February 2020

Greenpeace Romania Reports, Illegal cuts of Romania's forests, 2009-2011, 2012, 2013-2014, 2015, 2016 2017, Available at: https://www.greenpeace.org/romania/ro/campanii/paduri/publicatii/

Greenpeace Romania. 2020. Forbidding forest exploitation. The Greenpeace perspective, retreieved from https://www.greenpeace.org/romania/articol/2706/interzicerea-exploatarilor-forestiere-pozitia-greenpeace-romania/ on the 23rf of April 2020

Iojă, C. I., Pătroescu, M., Rozylowicz, L., Popescu, V. D., Vergheleţ, M., Zotta, M. I. and Felciuc, M. 2010. The efficacy of Romania's protected areas network in conserving biodiversity. *Biological Conservation*, *143*(11), 2468-2476.

Ioras, F. and Abrudan, I. V. 2006. The Romanian forestry sector: privatisation facts. International Forestry Review, 8(3), 361-367.

Knorn, J., Kuemmerle, T., Radeloff, V. C., Szabo, A., Mindrescu, M., Keeton, W. S., ... and Hostert, P. 2012. Forest restitution and protected area effectiveness in post-socialist Romania. *Biological Conservation* 146(1): 204-212.

Lawrence, A. 2009. Forestry in transition: Imperial legacy and negotiated expertise in Romania and Poland. Forest Policy and Economics, 11(5-6), 429-436.

Maiorano, L., Falcucci, A., Garton, E. O. and Boitani, L. 2007. Contribution of the Natura 2000 network to biodiversity conservation in Italy. *Conservation biology 21*(6):1433-1444.

Manolache, S., Ciocanea, C. M., Rozylowicz, L. and Nita, A. 2017. Natura 2000 in Romania–a decade of governance challenges. *European Journal of Geography 8*(2):24-34.

Manolache, S., Nita, A., Ciocanea, C. M., Popescu, V. D., and Rozylowicz, L. 2018. Power, influence and structure in Natura 2000 governance networks. A comparative analysis of two protected areas in Romania. *Journal of environmental management*, *212*:54-64..Munteanu, C.,

Nita, M.D., Abrudan, I.V. and Radeloff, V.C. 2016. Historical forest management in Romania is imposing strong legacies on contemporary forests and their management. *Forest Ecology and Management* 361: 179-193.

MWF (Ministry of Waters and Forests). 2015. GIS data regarding protected areas, Natura 2000 sites, retrieved from http://www.mmediu.ro/articol/date-gis/434 on the 25th of March 2020

MWF. 2017. Raport privind starea pădurilor României (Report regarding the state of Romanian forests). Ministry of Waters and Forests, Bucharest [in Romanian], 82 pp., Available on-line at. http://apepaduri.gov.ro/paduri/

Nichiforel, L., Keary, K., Deuffic, P., Weiss, G., Thorsen, B.J., Winkel, G., ... and Mifsud, E.G. 2018. How private are Europe's private forests? A comparative property rights analysis. *Land use policy* 76: 535-552.

NIS (National Institute of Statistics). 2018. Available at http://statistici.insse.ro:8077/tempo-online/#/pages/tables/insse-table, retrieved on the 30th of April 2020

Niţă, M.A. 2015. Good governance and forest exploitation in Romania. A comparative analysis. *Procedia Economics and Finance* 32:795-800.

Paavola, J., Gouldson, A., & Kluvánková-Oravská, T. (2009). Interplay of actors, scales, frameworks and regimes in the governance of biodiversity. *Environmental Policy and Governance 19*(3):148-158.

Pierce, S. M., Cowling, R. M., Knight, A. T., Lombard, A. T., Rouget, M. and Wolf, T. 2005. Systematic conservation planning products for land-use planning: interpretation for implementation. *Biological Conservation 125*(4):441-458.

Popa, B., Niță, M.D. and Hălălișan, A.F. 2019. Intentions to engage in forest law enforcement in Romania: An application of the theory of planned behavior. *Forest Policy and Economics* 100: 33-43.

Primack, R. B., Pătroescu, M., Rozylowicz, L., and Ioja, C. 2008. Fundamentele conservării diversităţii biologice, Edit. *Agir, Bucureşti*, *668*.

Sutherland, W. J., Adams, W. M., Aronson, R. B., Aveling, R., Blackburn, T. M., Broad, S., ... and Dinerstein, E. 2009. One hundred questions of importance to the conservation of global biological diversity. *Conservation Biology 23*(3):557-567.

Young, J., Richards, C., Fischer, A., Halada, L., Kull, T., Kuzniar, A., ... and Watt, A. 2007. Conflicts between biodiversity conservation and human activities in the central and eastern European countries. *AMBIO: A Journal of the Human Environment 36*(7):545-550.

Wilson, K. A., Cabeza, M. and Klein, C. J. 2009. Fundamental concepts of spatial conservation prioritization. *Spatial conservation prioritization: quantitative methods and computational tools. Oxford University Press, New York*, 16-27.

Russia – Arkhangelsk oblast (2)
By Denis Dobrynin and Galina Mikhaylova

Case study description
The Arkhangelsk Region is located in Northwest Russia. From south to north the Arkhangelsk region (587,400 km^2) includes three ecoregions: the middle boreal (102,750 km^2), the northern boreal (154,430 km^2), and forest-tundra (36,140 km^2). In accordance with the regional Forest Plan, area of officially designated 'forest fund' in the Arkhangelsk region is 285,012 km^2, of which 222,208 km^2 are covered with forest stands (the rest of 62,804 km^2 are represented by non-forest ecosystems, such as mires).As a part of the national legislation, forests are divided into exploitative (commercial) and protective forests. In the Arkhangelsk region, the area of the exploitative forests is 160,829 km^2 (72 %), while protective forests cover 61,379 km^2 (28%).(Forest Plan ..., 2018; Federal ... 2019). Coniferous forest stands with Norway spruce (*Picea abies*) and Scotspine (*Pinus sylvestris*) dominate, while larch (Larix decidua) and fir (Abies sibirica) are sporadic. Deciduous forest stands are mainly young and middle-age secondary-growth stands occurred because of clear-cutting and represented by birch (*Betula spp*.) and aspen (*Populus tremula*). Large primary unfragmented forests of the Arkhangelsk region meet the criteria of Intact Forest Landscapes (Yaroshenko et al. 2001). As of 2000 and 2013, the Arkhangelsk Region harboured 9,482 km^2 vs. 8,788 km^2, respectively (Aksenov et al. 2002, WWF 2016). These hosts the last wild forest reindeer in Europe (Zagidullina et al. 2018, Mamontov 2018).

Portfolios of conservation instruments and area proportions (question 1)
According to national legislation, the forests of the Arkhangelsk region are divided into two types, including protective (95,220 km^2 or 32.5%) and exploited (commercial) forests (198,100 km^2 or 67.5%) (RUS-Arkh 1). The key functions of protective forests are environmental maintenance, water conservation, as well as sanitary and health-improving functions. Almost all the forest-tundra forests are classified as protective forests. The key function of exploited forests is forestry for the production of raw material for the forest industry. In addition to protective forests, a network of specially protected forest sites is established within both the protective and exploited forests. These forests are mainly coastal protection areas located along water bodies, ravines, the most valuable areas of protected areas, etc. (Forest Plan ..., 2018). In addition to legally designated forest protection, there are non-legally binding moratoria zones under non-state voluntary certification schemes. In the Arkhangelsk region, the area of certified forests according to FSC and PEFC standards is about 70,000 km^2 or about 35% of the forests with productive functions (Forest regions ..., 2018). The FSC standards require conservation of intact forest landscapes as one of the categories of the high conservation values. The total proportion of such voluntarily set-asides forests is 0.8% (Table RUS-Arkh 1).

Forests in the Russian Federation are owned by the state, and forest user leases forest land for shorter or longer periods of time. For an overview of the institutional arrangements that regulate the forest lease and related markets of industrial timber in Russia under the new 2007 Forest Code, see Torniainen and Saastamoinen (2007).

Effectiveness (question 2)
Forests of protected areas comprise about 25% of all the protective forests (95,220 km^2) of the Arkhangelsk Region (Table RUS-Arkh 1). In Russia, protected areas are established on the

federal and regional levels. Federal level protected areas are governed by the Ministry of Natural Resources of the Russian Federation. This category covers 7,130 km^2 (2.4% of forest land) and they are represented by three national parks and one strict nature reserve (Pinega 'zapovednik'). To decrease the edge effect on ecosystems of federal protected areas buffer zones with some nature use restrictions are created around national parks and strict nature reserves. Two national parks in the Arkhangelsk region have the status of biosphere reserves. On the one hand, national parks can contribute to the well-being of the local communities and provide livelihoods by developing the use of non-timber forest products and ecotourism (Михайлова/Mikhailova and Efimov, 2015). However, conflicts between local communities and parks' administration occur in the Arkhangelsk Region. The conflicts are caused by restrictions of nature use types that locals consider as traditional (customary). Regional level protected areas cover 19,811 km^2 and they are represented by 35 nature reserves ('*zakazniks'*) and 66 nature monuments.

Protected area networks in the Arkhangelsk Region aim at conservation of natural ecologically valuable ecosystems, rare and endangered species, as well as providing multiple ecosystem services. The protected area network of the Arkhangelsk region is fragmented and poor in related to landscape representativeness (Efimov/Ефимов, 2017; Razumovsky/Разумовский, 2000; Dobrynin/Добрынин, 2008; Bogolitsyn/Боголицын 2011). However, the landscape representation was significantly increased in the 2010s by the creation of a range of protected areas in various parts of the region. There are difficulties regarding environmental monitoring and control over protected areas management regarding regulation of hunting, fishing, and tourism within protected areas. Moreover, the boundaries of the regional level nature reserves can be changed, and their protection mode can be weakened by lobbying mining companies (WWF insists ..., 2018; Flora and fauna ..., 2017).

In addition to protected areas, a vast network of protective forests contributes to biodiversity conservation in the region (see Naumov et al. 2017: Table A1 on p. 1349). Therefore, protective forests, especially water protection zones, are considered as ecological corridors for species migration and connecting protected areas. For instance, in the south part of the Arkhangelsk region, the primary forests remain mainly within protective forests (within water protection forests). However, decreasing of sizes of water protection zones in accordance with changes in the state forestry norms (2007 Forest Code) are able to decline the efficiency of biodiversity conservation within the protective forests (e.g., Naumov et al. 2017).
FSC as market-driven voluntary forest certification scheme contributes to primary forest and biodiversity conservation in addition to state forest and environmental governance tools…

Table RUS-Arkh 1. Legally designated and voluntary forest conservation in the Arkhangelsk region, Russia.

	Legally designated conservation			Voluntary conservation under FSC	Commercial forests
	Protective forests, including protected areas			Non-legally binding logging ban zones (outside of the existed protected areas)	
	Protected areas		Other categories of protective forests		
	Federal level	Regional level			
Area, km^2	7,130	19,820	68,270	2,400	198,100 – 2,400
Share (%)	2.4	6.8	23.3	0.8	67.5 – 0.8

Policy implementation tools (question 3)

'Carrot' and 'Stick': Forest management certification systems, and in particular Forest Stewardship Council, are often considered as a 'carrot' for timber companies in many regions of the world. For some companies, environmentally sensitive markets are the main or only possible markets for certain types of products. 'Carrot' is seen as getting access to so-called environmentally sensitive markets. As opposed to eastern Russia this is true in the case of North-West Russia, where the forest sector is focused on European eco-sensitive markets that require FSC certificates (Debkov 2019). In these cases, 'non-state market-driven forest governance systems' (Cashore, 2002) can play the role of a 'stick' simultaneously with state regulation: This means that, once a company has been certified, voluntary FSC standards are no longer voluntary. As a result, forest management and forest conservation practices in North-West Russia are shaped by both state norms, as well as 'non-state market-driven' standards. For instance, FSC requires to define and to exclude from forest exploitation core areas of so called intact forest landscapes (Yaroshenko et al. 2001) that it is not required by national law. Core areas are defined by companies in collaboration with environmental NGOs. Conservation of intact forest landscapes is implemented within the framework of non-legally binding moratoria agreements between FSC-certified companies and NGOs. Later, some of moratoria zones become a basis for the establishment of protected areas by the state agencies. The creation of a >3,000 km^2 regional level protected area in 2019 in the Dvina-Pinega intact forest landscape, which shrunk from 11,460 km^2 in 2000 to 7,705 km^2 in 2017 (Karpov 2019), is a good example.

New protected areas are created by the state inter alia to contribute to the implementation of intergovernmental commitments which can be considered as a 'stick' for the states. For instance, the Russian action plan for the implementation of the concept for the development of federal protected areas for the period until 2020[2] is considered as a contribution to the implementation of the CBD Aichi Biodiversity Target #11. Two national parks were created in North-West Russia within the borders of intact forest landscapes under the action plan in the 2010s. These were the Onezhskoe Pomorie in the Arkhangelsk region in 2013, and the only south taiga intact forest

[2] http://government.ru/docs/4281/

landscape Koigorodsky in the Republic of Komi in 2019. However, initially, at the planning stage of both protected areas, their landscapes were temporary conserved as moratoria zones under FSC certification. Hence, the roles of state and non-state (forest) policy instruments cannot always be clearly divided between ‘carrot’ and ‘stick’. As the North-West Russian case demonstrates, they can be intertwined and complement each other.

‘Sermon’: NGO-driven fora can also be considered as ‘sermon’ type of policy instruments in North-West Russia. The first example is WWF-driven Boreal Forest Platform[3] - a professional multi-stakeholder forum on the development of sustainable forest management and protection of high conservation value forests. The forum is focused on the transition to intensive sustainable forest management and conservation of intact forest landscapes in Russia. Activities of the Boreal forest platform comprise seminars and study tours. Another forum is Forest Dialog[4] leading by a domestic environmental NGO (Silver Taiga Foundation) (Angelstam et al. 2019). Forest Dialog is a regional multi-actor platform to discuss forest-related conflicts and find trade-offs between use and conservation of forest in the Republic of Komi.

Another example of ‘sermon’ is a set of regional guidelines on biodiversity maintenance while wood harvesting elaborated by NGOs for forest users in North-West Russia. Biodiversity conservation (including key biotopes and retention trees) are discussed within the framework of seminars and study tours conducted by NGOs and concession holders in North-West Russia since the end of the 1990s. Moreover, NGO-led recommendations were adopted and adapted by state forest governance agencies in North-West Russia, and recommendations on biodiversity conservation were included in regional forest plans. Hence, biodiversity conservation efforts initially driven by NGOs went a long way through 'path dependency' (when forest biodiversity was not recognized as a value) and finally were institutionalised by state governance agencies. In other words, NGO-led ‘sermon’ became state-led ‘sermon’. However, the efficiency of practical implementation of biodiversity conservation in commercial forests is still questionable (Blumroeder et al. 2018, 2019, 2020).

The intergovernmental region-to-region collaboration between Nordic countries and Russia within the framework of the Barents Euro-Arctic Council (BEAC) can be also considered as a ‘sermon’ tool. This collaboration includes the activity of the working group on environment and subgroup on nature and water. The key event of the subgroup is biennially held Habitat Contact Forum (HCF). The HCF is a platform where researchers, state governance agencies, and civil society organizations exchange data on the forest, wetland, and coastal ecosystems conservation in the Barents region. One of the key initiatives of the subgroup in the 2010s was the Barents protected area network (BPAN). Within the framework of the BPAN data on protected areas of the Barents region, including borders, categories, and protection modes, was produced to estimate the representativeness of the protected area network across national and regional borders (Aksenov et al., 2014). In addition, the analysis of protected areas and high conservation value forests, and its connectivity was done (Kuhmonen et. al, 2017). The outcomes of the BEAC subgroup on nature and water are represented and discussed on the level of BEAC meetings of the ministers of the environment. Thus, the BEAC does not imply a mandate to be a

---

[3] http://borealforestplatform.org/en/
[4] https://environment.yale.edu/news/article/effective-dialogue-in-forests-landscapes-10-years-of-experience-in-komi-russia/

‘stick’ or a source to provide a ‘carrot’. Rather it plays a role of ‘sermon’ spreading the nature conservation ideas, categorizations, and concepts horizontally, i.e. between actors in regions and countries of the Barents region.

One of such ideas was a scientist-led concept of so-called ‘green belts’ that need to be established in the Barents region to conserve biodiversity. The idea was considered as a way to improve the connectivity among national parks and nature reserves. The concept of ‘green belts’ rooted in conservation biology where the protected areas are seen as a complex of functionally and territorially interconnected conservation areas, with the representation of various types of ecosystems and landscapes (Shtilmark, 1981). The idea of green belts was actively used in the 2000-2010s by academia experts in Russia as a ‘sermon’ to convince decision-makers to establish planned protected areas aimed to the conservation of intact forest landscapes and other types of primary forests (Kurhinen/Курхинен, 2009; Razumovsky/Разумовский 2000; Efimov/Ефимов 2017; Bogolitsyn/ Боголицын 2011, Kobyakov and Jakolev 2013).

Net effect of conservation and intensification (question 4)

As table Table RUS-Arkh 1demonstrates the protected areas (PAs) cover 9.2% of the forest zone of the Arkhangelsk region (i.e. without Arctic islands). This figure is quite far from the 17% prescribed by the CBD Aichi target 11. Moreover, the protection regime of protected areas should be taking into account to estimate the net effect of conservation. According to Aksenov et al. (2014) the division of PAs of the Arkhangelsk region by strong, medium, and weak protection is 46%, 52.89%, and 2%, respectively. Thus, in accordance with this classification, logging, mining, construction and other activities that negatively affect habitats are prohibited within less than half of the area covered by PAs in the region. In this context, the problem of “Paper Parks” and “Fortress Conservation” is relevant. The problem of the former refers to PAs that are officially designated, but because of a weak protection regime does not provide biodiversity conservation. The problem of the latter relates to the protected areas PAs where ecosystems function in isolation from human activities, but local communities are considered as poachers or squatters using nature in destructive ways threatening to biodiversity.

However, the CBD Aichi target 11 is not only about protected areas. It implies “*other effective area-based conservation measures … integrated into the wider landscape…*”. In the context, the rest of the categories of legally designated protective forests, such as water protective forests, can be considered as the contribution to the CBD Aichi target 11. In the Arkhangelsk region, the share of such forests counts 23.3% (RUS-Arkh 1). Therefore, the total area of all types of legally designated PAs is 32.5% of the forest zone of the Arkhangelsk region. However, the protection regime of such forests allows sanitary felling that is not always conducted in a sustainable way. Besides, the size of some categories of protective forests tends to decrease as a result of changes in state forest management norms (Naumov et al. 2017).

In addition, non-legally binding moratoria agreements between companies and environmental NGOs under FSC add about 2,400 km^2 of conserved forest or about 0.8% of the forest zone of the region. Some of the previous moratoria zones played a significant role in the creation of protected areas in the region.

References

Aksenov, D., Dobrynin, D., Dubinin, M., Egorov, A., Isaev, A., Karpachevskiy, M., Laestadius, L., Potapov, P., Purekhovskiy, A., Turubanova, S. and Yaroshenko, A. 2002. Atlas of Russia's intact forest landscapes. Biodiversity Conservation Center, Moscow.

Aksenov, D., Kuhmonen, A., Mikkola, J. and Sobolev, N. 2014. The characteristics and representativeness of the protected area network in the Barents region. *Reports of the Finnish Environment Institute*, Helsinki, 29, 1-189. https://helda.helsinki.fi/handle/10138/156287 Accessed 29.04.2020

Angelstam, P., Manton, M., Cruz, F., Fedoriak, M. and Pautov, Yu. 2019. Learning landscape approach through evaluation: Opportunities for Pan-European Long-Term Socio-Ecological Research. Chapter 12 in: Mueller, L., Eulenstein, F. (ed.) Current trends in landscape research. Springer. (https://www.springer.com/gp/book/9783030300685)

Blumröder, J. S., Hoffmann, M. T., Ilina, O., Winter, S., Hobson, P. R. and Ibisch, P. L. 2020. Clearcuts and related secondary dieback undermine the ecological effectiveness of FSC certification in a boreal forest. *Ecological Processes*, 9(1), 1-9.

Blumroeder, J. S., Burova, N., Winter, S., Goroncy, A., Hobson, P. R., Shegolev, A., ... and Volkov, A. (2019). Ecological effects of clearcutting practices in a boreal forest (Arkhangelsk Region, Russian Federation) both with and without FSC certification. *Ecological Indicators*, 106, 105461.

Blumroeder, J. S., Hobson, P. R., Graebener, U. F., Krueger, J. A., Dobrynin, D., Burova, N., ... and Ibisch, P. L. 2018. Towards the evaluation of the ecological effectiveness of the principles, criteria and indicators (PCI) of the forest stewardship council (FSC): Case study in the Arkhangelsk region in the Russian federation. *Challenges in Sustainability* 6(1):20-51.¨

Cashore, B. 2002. Legitimacy and the privatization of environmental governance: How non–state market–driven (NSMD) governance systems gain rule–making authority. *Governance* 15(4):503-529.

Debkov, N. 2019. State of forest management certification in Russia by the end of 2016. Forestry Ideas 25(1):20–36.

Intact forest landscapes in Russia: current condition and losses over the last 13 years (2016) Poster, A1. Moscow, World Wildlife Fund (WWF Russia), https://wwf.ru/en/resources/publications/booklets/intact-forest-landscapes-in-russia-current-condition-and-losses-over-the-last-13-years/ Accessed 28.04.2020

Karpov, A.A. 2019. Area reduction of intact forest landscapes on the example of the Dvina-Pinega interfluve. Lesnoy Zhurnal [Forestry Journal] 3: 152–158. DOI: 10.17238/issn0536-1036.2019.3.152 (in Russian)

Kobyakov, K. and Jakolev, J. 2013. Atlas of high conservation value areas, and analysis of gaps and representativeness of the protected area network in northwest Russia. Finnish Environment Institute.

Kuhmonen, A., Mikkola, J., Storrank, B. and Lindholm, T. 2017. Protected areas and high conservation value forests in the Barents Euro-Arctic Region—Sweden, Finland and Russia. Reports of the Finnish Environment Institute, 33. https://helda.helsinki.fi/handle/10138/229432 Accessed 01.05.2020

Mikhailova G.V. and Efimov V.A. 2015.Social Assessment of Specially Protected Natural Areas. *Economic and Social Changes: Facts, Trends, Forecast* 4 (40):151-164. DOI: 10.15838/esc/2015.4.40.10

Naumov, V., Angelstam, P. and Elbakidze, M. 2017. Satisfying rival objectives in forestry in the Komi Republic: Effects of Russian zoning policy change on forestry intensification and riparian forest conservation. *Canadian Journal of Forest Research* 47: 1339–1349. https://doi.org/10.1139/cjfr-2016-0516

Torniainen, T. J. and Saastamoinen, O. J. 2007. Formal and informal institutions and their hierarchy in the regulation of the forest lease in Russia. *Forestry: An International Journal of Forest Research* 80(5):489-501.

Trishkin, M., Karjalainen, T. and Kangas, J. 2019. An Analysis of the Non-conformities of Certified Companies Operating in Northwestern Russia. Forests, 10(12), 1061.

WWF настаивает на проведении повторных общественных слушаний по Соянскому и Приморскому заказникам / Официальный сайт WWF. 01 февраля 2018. – URL: https://wwf.ru/resources/news/barents/wwf-nastaivaet-na-provedenii-povtornykh-obshchestvennykh-slushaniy-po-soyanskomu-i-primorskomu-zakaz/

Yaroshenko, A. Y., Potapov, P. V. and Turubanova, S. A. 2001. Last intact forest landscapes of Northern European Russia. Global Forest Watch.

Глибко О.Я., Барсова А.В. Органзация и ведение экологического мониторинга на территории национальных парков // Междисциплинарный научный и прикладной журнал «Биосфера». 2015. т. 7. № 3 С.321-327 – URL: https://cyberleninka.ru/article/n/organizatsiya-i-vedenie-ekologicheskogo-monitoringa-na-territorii-natsionalnyh-parkov

Государственная программа Архангельской области «Развитие лесного комплекса Архангельской области (2014 - 2024 годы)». Утверждена постановлением Правительства Архангельской области от 11 октября 2018 года №466-пп. – URL: http://docs.cntd.ru/document/462604782

Громцев А.Н. и др. О единой межрегиональной системе особо охраняемых природных территорий на Европейском Севере // Труды Карельского НЦ РАН. Серия биогеография. 2011. Вып. 12. С. 4-11. – URL: http://transactions.krc.karelia.ru/publ.php?plang=r&id=8634

Добрынин Д.А., Столповский А.П. Ландшафтное разнообразие и система ООПТ Архангельской области. Архангельск, 2008. 36 с. – URL: https://wwf.ru/upload/iblock/293/oopt_arkh.pdf

Ефимов В.А. Особенности и проблемы создания системы особо охраняемых природных территорий в Архангельской области // Вклад особо охраняемых природных территорий Архангельской области в сохранение природного и культурного наследия: материалы докладов межрегиональной научной конференции, посвящённой 100-летию заповедной системы России. 2017. С. 11-15. – URL: https://www.elibrary.ru/item.asp?id=32604940

Курхинен Ю., Громцев А., Данилов П. и др. Особенности и значение таежных коридоров в Восточной Фенноскандии // Труды КарНЦ РАН. 2009. № 2. С. 16–23. – URL: http://resources.krc.karelia.ru/transactions/doc/trudy2009/trudy_200902_016-23.pdf

Лесной план Архангельской области Российской Федерации на 2019 – 2028 годы / Правительство Архангельской области. Министерство природных ресурсов и лесопромышленного комплекса Архангельской области. 2018. – URL: https://dvinaland.ru/gov/iogv/minlpk/docList/

Лесные регионы: Архангельская область / ООО «Национальное лесное агентство развития и инвестиций» // Лесной комплекс РФ. 2018. 30 марта. – URL: https://forestcomplex.ru/2018/03/lesnyie-regionyi-arhangelskaya-oblast/

Лесоустроительная инструкция. Утверждена приказом Минприроды России от 29.03.2018 N 122 (ред. от 06.02.2020) (Зарегистрировано в Минюсте России 20.04.2018 N 50859). – URL: https://sudact.ru/law/prikaz-minprirody-rossii-ot-29032018-n-122/lesoustroitelnaia-instruktsiia/iii/

Разумовский В.М., Ефимов В.А. Особо охраняемые природные территории и сохранение природного разнообразия в Баренц-Арктическом регионе России: состояние и перспективы // Поморье в Баренц-регионе. Экономика, экология, культура / Матер. межд. конф. Архангельск, 2000 С. 192.

Федеральная экологическая информация Архангельской области / Федеральная служба по надзору в сфере природопользования. – URL: http://23.rpn.gov.ru/node/5927

Фрола и растительность Беломорско-Кулойского плато /Т.Ю. Браславская, С.В. Горячкин, С.А. Кутенков и др.; Северный (Арктический) университет. Архангельск: САФУ, 2017. 302 с. – URL: https://www.elibrary.ru/item.asp?id=29167820

Штильмарк Ф.Р. Принципы заповедности (теоретические, правовые и практические). М.: Главохота РСФСР, 1981. 234 с.

Russia – Murmansk oblast (1)

By Eugene Borovichev, Victor Petrov and Olga Petrova

Case study description

The Murmansk Region (145,000 km^2) is located in the far north-west of Russia, and in the north-eastern corner of Fennoscandia bedrock shield. The relief is hilly, with the Khibiny and Lovozero Mountains in the central part of the region rising to 1,200 m above the sea level (a.s.l.), and more flat in the east. The region has three vegetation zones (see Ahti et al. 1968): (1) the northern boreal zone (taiga), (2) forest-tundra, i.e. the northern forest border which is formed by birch (*Betula spp.*) stands, as is typical for areas with an oceanic and sub-oceanic climate, and (3) tundra (Figure RU-Mu 1). The south-western and central parts of Murmansk region are mainly covered by pine (*Pinus silvestris*) and spruce (*Picea abies*) forests. The forest tundra in the north extends from north-west to south-east in a 20-100 km wide belt. In all mountain areas, vertical vegetation zones are distinguished. The foothills are dominated by forests. Higher up, at an altitude of 300–450 m a.s.l., stunted birch forest covers steeper slopes in wedges. The upper part of the slopes (over 400–450 m a.s.l.) and peaks are covered by mountain tundra with vast rock fields, detritus and bedrock outcrops.

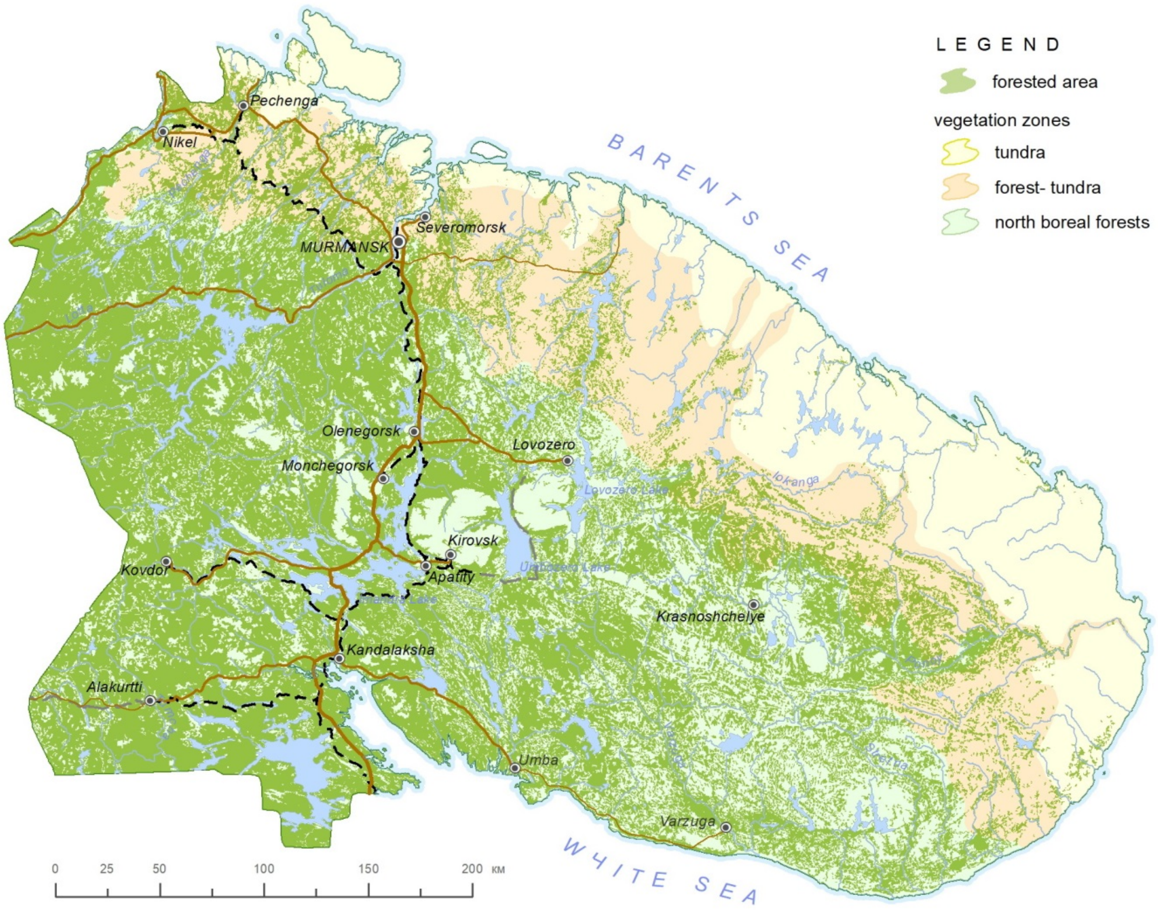

Figure RU-Mu 1. Vegetation zones in the Murmansk Region

The Murmansk Region is an area with the largest enterprises based on mineral resources of the Arctic zone of the Russian Federation, and of the country as a whole. The region supports a

major share of the national economy demand for phosphate ore, zirconium (baddeleyite), niobium, tantalum, and rare-earth metals. Copper-nickel and iron ores, nepheline and ceramic raw materials, facing stone and building materials are also extracted. The region's mining industry sites and operations are the main employer in many cities and towns, where a third of the region's population resides, and its output represents more than 60 % of the region's industrial production.

Forest industry is currently an undeveloped sector. The total forest area is 51,856 km^2 (35.8% of Murmansk Region), and the productive forest area (Net Primary Productivity average for 2000-2014, 300g C/m^2 according dataset MOD17A3 (Numerical Terradynamic Simulation Group / University of Montana 2014)) is less than 30% (Figure RU-Mu 2). The majority of the forests therefore have limited importance for commercial timber extraction. Because in the Murmansk Region the volumes of commercial timber harvesting have declined and are now small (Angelstam et al. 2020), there are no large logging enterprises. Forest Certification is absent in Murmansk Region.

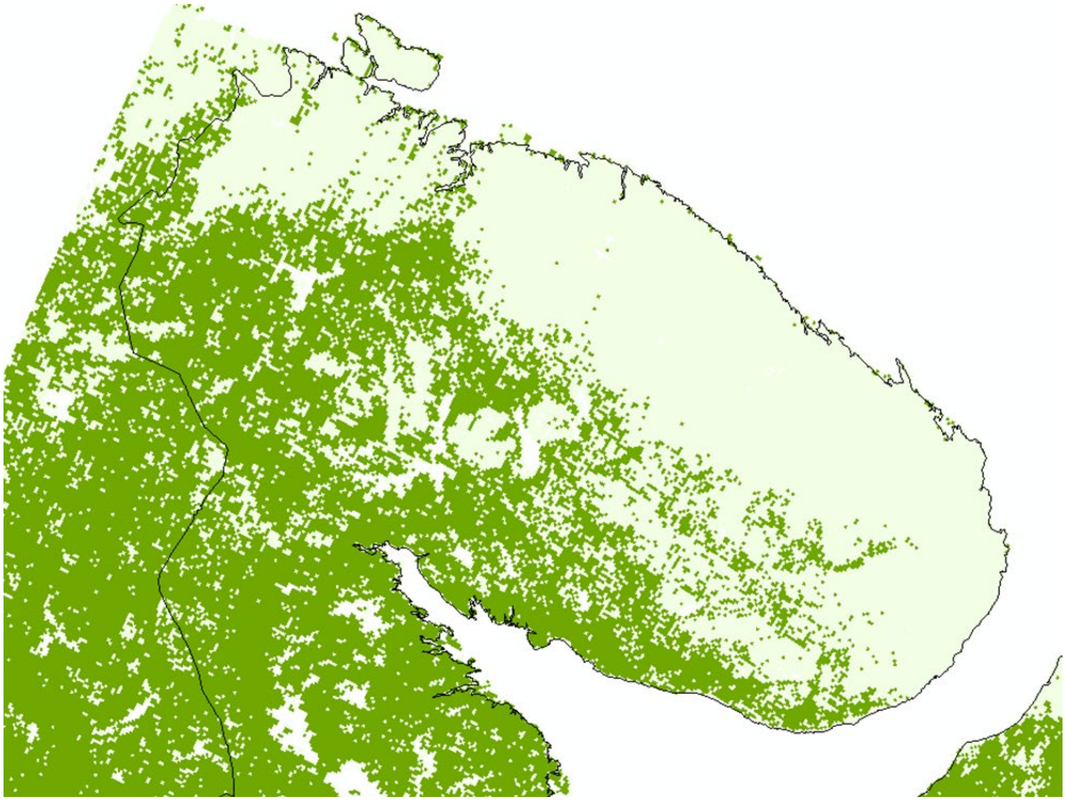

Figure RU-Mu 2. The productive forest area (300 g according to MODIS data).

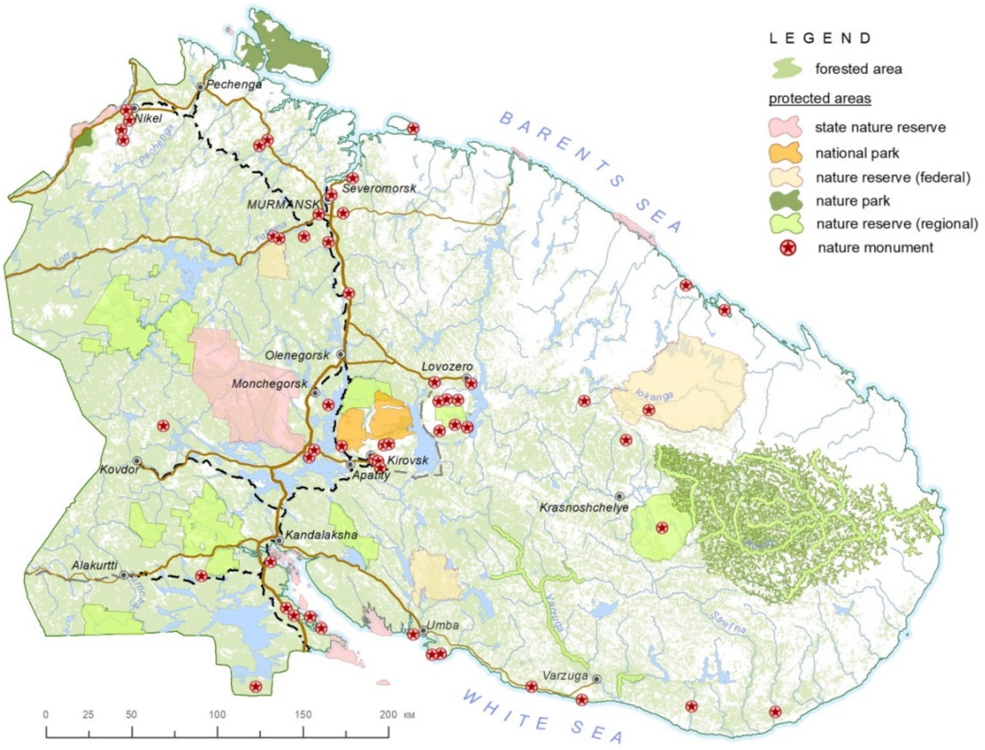

Figure RU-Mu 3. Current network of protected areas in the Murmansk Region.

Portfolios of conservation instruments and area proportions (question 1)

The modern history of protected areas in the Murmansk Region dates back to the early 20th century. The progress and properties of the protected area network is related to the history of neighbouring states (Norway and Finland) and evolution of nature conservation ideas (Borovichev et al., 2018, 2019). Official tools for forest conservation are (1) formally protected areas, (2) protective forests according to the Federal Forest Code, which defines functional groups of forests, and (3) prescription for the protection of the rare species (Figure RU-Mu 3). It should be noted that according to Russian laws, if a red-book species is found, it is possible to create protected areas (which is a long and expensive process), or to apply for removal of particular localities from the prescription for the protection of the rare species according to the red data book species in economically important areas.

The current network of protected areas in the Murmansk Region includes six categories of conservation instruments: (i) State Nature Reserve (zapovednik (e.g., Shtil′mark 2003, Ostergren and Hollenhorst 2017)) (Lapland, Kandalaksha and Pasvik), (ii) National Park (Khibiny), (iii) natural park (Korablekk, Rybachy and Sredny Peninsulas), (iv) State Sanctuary (Zakaznik) (n=12), (v) (Nature Monument (n=55), and (vi) the Polar-Alpine Botanical Garden of Kola Science Centre of RAS. As of May 2020, the total area of protected areas in the Murmansk Region is 20,999 km^2, or 13.4% of the region’s total land area (145,000 km^2) (Table RU-Mu 1). According to Russian law, federal protected areas, i.e. State Nature Reserves, National Parks as well as Botanical Garden, are excluded from the forest fund. But they also contribute to the preservation of forest communities.

Protective forests being considered formally protected include water-protection forests, forests located in forest-tundra zones and mountains, and scientifically and historically important forests (Table RU-Mu 2.). Recent additions to forest legislation of Russian Federation as to water-protected forests (spawning forest zones) can significantly reduce their ecological and environmental value because wood harvesting is possible (Naumov et al. 2017). According to an analysis of the correspondence between the categories of protective forests and IUCN categories, it was concluded that the categories of valuable forests correspond to IUCN categories (Stishov and Dadly 2018). Therefore water-protection forests and riparian corridors and buffer zones located along water objects are not included in the analysis.

One more method for forest protection is issuing prescriptions limiting economic activity in certain areas to prevent disturbance to identified protected species of plants, lichens, fungi, and animals (Red Data Book..., 2014). In 2018, the Ministry of Natural Resources of the Murmansk region issued its first after 2003 prescription for the protection of the rare species of vascular plants inhabiting the Luvenga Swamp in the Kandalaksha District (Makarov et al., 2020).

Effectiveness (question 2)

We assessed the effectiveness of protected area based on the correspondence of the protection regimes to the threats to these territories. Despite almost 14% of Murmansk Region being protected, the protected area network is suboptimal and not very effective. At the present level of efficiency, even achieving a close to scientifically based area figure of PAs (13.4% of the Murmansk Region's area (Table RU-Mu 1), and these protected areas' forests of the forest area being 40%).), it is hardly possible to guarantee the proper level of biodiversity conservation and the ecosystems stability in the region (Borovichev et al., 2018; Makarov et al., 2020). The most part of the Murmansk Region's forests are related to forest-tundra zones and mountains type. We divided all protection regimes into 4 groups (Figure RU-Mu 4): (1) any human activity is prohibited; (2) cutting, mining, building are prohibited; (3) protected area regime allows one or two of the following activities: i) cutting, ii) mining, or iii) building; (4) protected area regime allows all of the following activities: i) cutting, ii) mining, ii) building. Only the State Nature Reserves and the National park, one of the two Natural parks in the region, two Zakazniks, and seven nature monuments can effectively perform their environmental protection functions (their combined area is only 4.2 % of the Murmansk region's area, and these areas' forests of the forest area is 13%). The protection regimes in the other protected areas fail to match the existing threats.

Table RU-Mu 1. Protected areas located within the forest fund and outside it in Murmansk region.

	Within the forest fund			Outside the forest fund		
Protected area category	Number	Area (km^2)	% of Murmansk Region area 145,000 km^2	Number	Area (km^2)	% of Murmansk Region area 145,000 km^2
(i-a) State Nature Reserve (zapovednik)	–	–	–	two wholly, one partly	3,597	2.4
(i-b) conservation area of State Nature Biosphere Reserve	–	–	–	1	280	0.2
(ii) National Park	–	–	–	1	848	0.6
(iii) Natural Park	2	914	0.6			
(iv-a) Federal State Sanctuary (zakaznik)	3	3,997	2.7			
(iv-b) Regional State Sanctuary (zakaznik)	9	10,271	6.8			
(v) Nature monuments	55	179	0.12	0	0	0
(vi) Botanical garden	0	0	0	1	13	0
		**15,361**	10.2		4,738	3.2

intersection of areas different protected areas – 13983,97, this should be in hectares
Total forest area is 51,856 km^2; all oblast is 145,000 km^2,i.e. 35.8%.

Table RU-Mu 2. Types protective forests in Murmansk region

**Types of** protective **forests**	(Area in km^2)	Proportion of Murmansk region (145,000 km^2) (%)	Proportion of forest cover (51,856 km^2) (%)
in forest-tundra zones and mountains	4,335	3.0	8.4
scientific / historical important forests	9	~0	~0
spawning forest zones	1,234	0.9	2.4
especially protective forests	2	~0	~0
Sum	5,580	3.8	10.8

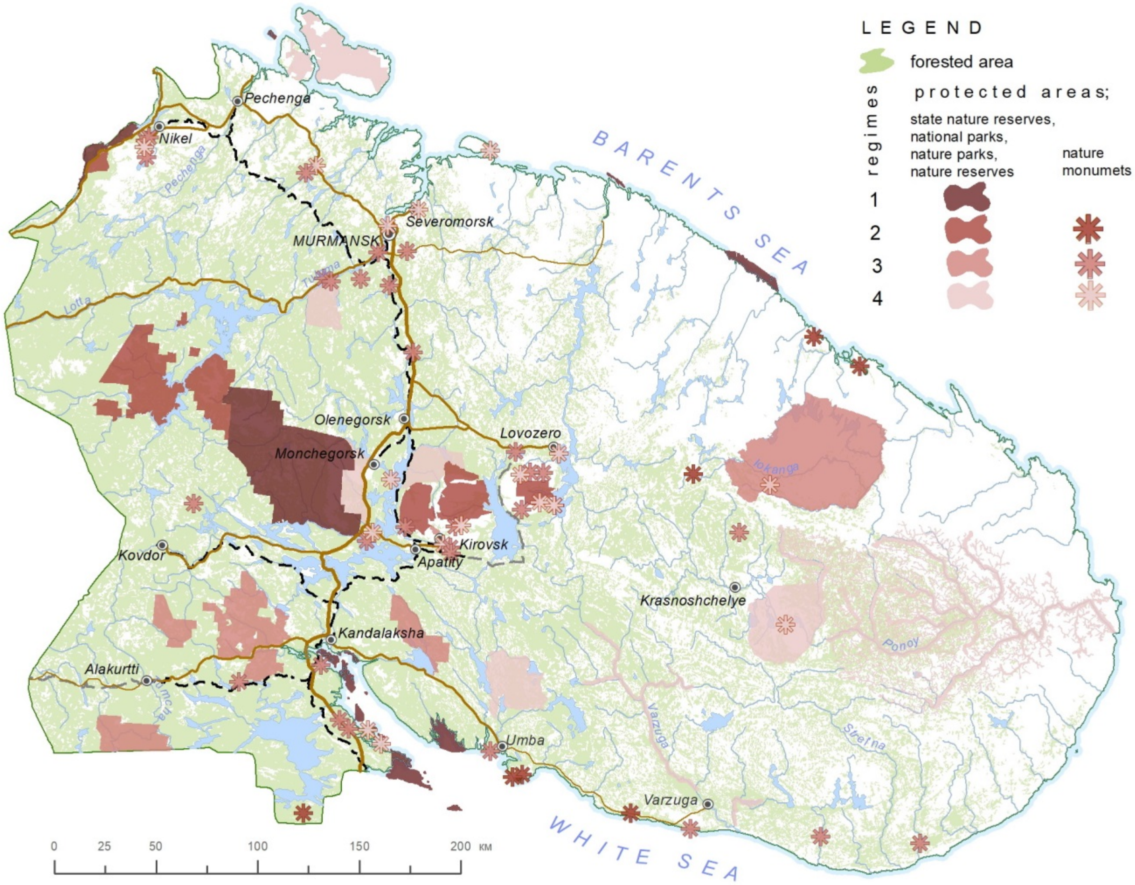

Figure RU-Mu 4. Assessment the effectiveness of Murmansk Region’s PAs

There are neither any formally defined voluntary protected forest areas, such as under a forest certification scheme (forest industry is almost undeveloped), nor tree retention areas. Unproductive forests are very common in Murmansk Region (forests located in forest-tundra zones and mountains), and make up 20%.

Policy implementation tools (question 3)

"Carrot": In the Murmansk Region, forestry does not have any significant share in the economy of the region. In our case, the "carrot" and "stick" are both related to the reputation of an entrepreneur performing logging. Entrepreneurs who do not carry out logging in old-growth forest have a reputation of being nature-oriented. The main problem is that logging companies are quite small, and the market is mainly Russia, where the "green face" of the entrepreneur does not play such an important role.

"Stick": The state and the oblast establish protected areas.

"Sermon": Active information activities to forest companies about the importance of preserving forest communities have been fruitful. "Sermon" was conducted by environmental organizations, and increased the level of environmental knowledge among loggers. However, these forest companies have not been very active recently.

Net effect of conservation and intensification (question 4)

Parts of protected areas are heavily managed, while state nature reserve (zapovednik) and sanctuaries (Zakaznik) only partially. The guarantee for no or very limited timber extraction is the geographic location of the region in the northern boreal zone with low annual growth increment class. As a result a slow decrease in forest biodiversity is estimated.

The Murmansk Region is a region where the environmental effect of regulating and supporting ecosystem services in protected areas is greatly reduced in the areas accommodating major mining and metals projects. The environmental impacts of the industry are not compensated by adequate environmental protection efforts. In the future forest resources such as biofuels may become a threat to forests of Murmansk Region.

References

Ahti, T., Hämet-Ahti, L. and Jalas, J. 1968. Vegetation zones and their sections in northwestern Europe. Annales Botanici Fennici 5:169-211.

Angelstam, P., Manton, M., Yamelynets, T. and Sørensen, O. J. 2020. Landscape Approach towards Integrated Conservation and Use of Primeval Forests: The Transboundary Kovda River Catchment in Russia and Finland. Land 9(5):144.

Borovichev, E. A., Petrov, V. N., Petrova, O. V. and Koroleva, N. E. 2018. Protected areas network in the Murmansk Region: yesterday, today, and tomorrow. *Arctic and North* 32:88-98.

Borovichev, E. A., Koroleva, N. E., Polikarpova, N. V., Petrov, V. N., Petrova, O. V. and Trusova, M. G. 2019. The network of protected areas in the Murmansk part of the Green Belt of the Fennoscandia: history, current situation and prospects. // Trans. KarRC RAS. № 4. P. 1–12. DOI: 10.17076/them1015

Naumov, V., Angelstam, P. and Elbakidze, M. 2017. Satisfying rival objectives in forestry in the Komi Republic: Effects of Russian zoning policy change on forestry intensification and riparian forest conservation. *Canadian Journal of Forest Research* 47: 1339–1349. https://doi.org/10.1139/cjfr-2016-0516

Shtil′mark, F. R. 2003. History of the Russian zapovedniks, 1895-1995. Russian Nature Press.

Stishov M.S., Dudley N. 2018. Protected areas of the Russian Federation and their categories. Moscow: WWF. 248 p.

Ostergren, D.M. and Hollenhorst, S. J. 1999. Convergence in Protected Area Policy: A Comparison of the Russian Zapovednik and American Wilderness Systems. *Society & Natural Resources* 12:4:293-313, DOI: 10.1080/089419299279614

Makarov, D. V., Borovichev, E. A., Klyuchnikova, E. M. and Masloboev, V. A. 2020. Environmental protection and sustainable development of the mining industry in Murmansk Region, Russia. // Vestnik of MSTU 23(1): 63–71. DOI: 10.21443/1560-9278-2020-23-1-63-71

Red Data Book of the Murmansk region. 2014. Eds. N. A. Konstantinov et al. Kemerovo: Asia-Print. 578 p. (In Russ.)

Slovak Republic (7)

By Pavel Mezei

Case study description

Forest cover of Slovakia is 41% (i.e. 20,128 km^2), and can be divided into two biogeographic regions, the Pannonian lowland region (14,366 km^2 of which 12.8% is forest) sheltered by the Alpine biogeographic region in the Carpathian Mountains (34,666 km^2 of which 52.7% is forest).

Portfolios of conservation instruments and area proportions (question 1)

The establishment of a network of nature reserves in former Czechoslovakia, of which Slovakia covers the eastern half, started mainly after the Second World War. Sites with the closest-to-nature conditions scientific importance were selected with research as the main aim of their conservation. This is explained by the fact that protected areas were under the Ministry of the Education (Mihálik 1968). There are 1,097 small-scale (< 1,000 ha; mean area 65 ha) protected areas covering 116,144 ha which is 2.37% of the country and 23 large-scale protected areas (> 1,000 ha; mean area 47,706 ha) covering 840,122 ha which is 22.49 % of the country. Additional protected areas were added after Slovakia joined the European Union and adopted the EU Birds directive and Habitat directive. These areas are referred as the "European Network of PAs", and in many cases they overlap the national protected areas. The 642 Special Areas of Conservation (Habitat Directive sites) in Slovakia cover 12.56 % of the country (615,262 ha; of which 83.9% are forest land and 6.3% are agricultural land) and the 41 Special Protection Areas (Birds Directive sites) cover 26.16% of the country (1,284,806 ha; of which 69.7% is forest land and 22.8% is agricultural land) (Lieskovská and Léniová 2018; Moravčík et al. 2019). For details, see Tables SK1 and SK2, and Figure SK1.

Table SK1. Basic information about four groups of conservation instruments in Slovakia, only forest areas. Data on the area of protected areas were derived from freely available GIS layers (ArcGIS online), and for forests derived from the GIS layers provided by the National Forest Centre

Protection level	1.	2.	3.	4.	5.	Sum (2. to 5.)
Aim	"Open country" – non protected areas	Landscape protected area - Biodiversity conservation in human-influenced landscape	National park - Conserve nature of high value	Natural monument - Conserve local areas of high values	Nature reserve – strictly protected areas with non-intervention management	
Area and proportion of all forest land in 2020	1,237,427 ha (61 %)	363,143 ha (18 %)	306,795 ha (15 %)	32, 467 ha (1.6 %)	84,861 ha (4.2%)	787 266 ha (39%)
Establishment	-	After 1973	After 1948	After 1951	After 1958	
Target size	-	> 1,000 ha	> 10,000 ha	Usually < 50 ha	< 1,000 ha	
Duration	-	Permanent	Permanent	Permanent	Permanent	
Decision by	-	Government	Government	Government	Government	
Control	-	State	State	State	State	
Monitoring	-	State Nature Conservancy	State Nature Conservancy	State Nature Conservancy	State Nature Conservancy	

Table SK2. Proportion of all forest conservation instruments in Slovakia.

	Conservation instruments (% of all land the respective ecoregion)				
	1. Strict protection	2. Other formal protection	3. Unproductive forest	4. Tree retention	SUM
Slovakia	4.2	52	not applicable, see question 4	not applicable, see question 4	56
Slovakia-Alpine (34,666 km^2; forests: 18,818 km^2)	4.2	55			59
Slovakia-Pannonian (14,366 km^2; forests: 2,060 km^2)	0.14	37			37

Effectiveness (question 2)

At present 23% of Slovakia's total area is under some protection status. The proportion of forests under some protection level is more than 50%, although strictly protected areas without any wood harvesting cover 4% of forests. A total of 72% of protected areas are located in mountainous areas (Lieskovská and Považan 2018). The national and the European networks of protected areas overlap by 42.3% (Fig. SK1). National parks cover 12% of forest land and landscape protected areas 26% of forest land. Nature reserves with the highest protection level are small in size (mean 126 ha). Only 0.47% of forest land (0.21% of the country) is covered by primeval forests; 70 % of these mapped primeval forests are strictly protected. Broadleaved primeval forests represent only 5% of those 0.47 %, and only one location is in the Pannonian biogeographical region (Mikoláš et al. 2019).

In general, protected areas in the Carpathians Mountain region, for example in Poland, Slovakia or Ukraine generally exhibit less deforestation than non-protected forests (Kuemmerle et al. 2007; Butsic et al. 2017) and disturbances in protected areas were smaller compared to their surroundings (Sommerfeld et al. 2018). Natural disturbances affected mainly conifer forests (Griffiths et al. 2014).

Policy implementation tools (question 3)

"Carrot": Various forest certification programs affect ca 70% of forests, especially the FSC system. It guarantees access to desirable markets and stimulates forest owners to set aside forested areas, according to the requirements of the certification mechanisms. Additionally, compensatory payments for Natura 2000 network have some potential.

"Stick": The number of policy instruments to deal with forest conservation and management has increased as a result of the introduction of Natura 2000. There are direct payments for private land owners whose forests are in the strictly protected areas with no-management (nature

reserves). In 2017, the direct payments reached 3 million € (Moravčík et al. 2019) A number of supporting tools for the territories in the Natura 2000 network have been introduced in the Rural Development Tool (Anonymous 2018). However, forest management has not changed dramatically and does not differ from the management outside the Natura 2000 network. Another tool is the national Environmental Fund, which funds management the suppressing of bark beetles in the surroundings of nature reserves, but only in the buffer zone (usually 100 meters around the nature reserve). This management was applied in the buffer zones of 44 nature reserves (Moravčík et al. 2019).

"Sermon": Education campaigns to enlighten the society and forest managers on the principles of nature conservation and Natura 2000 network seems to have been the most commonly used instrument. The Pro Silva organization tries to implement nature-based conservation, but this seems to be a long-lasting process.

Net effect of conservation and intensification (question 4)

Most of the Slovakian forests are managed forests (72 %). The other two categories are "protective forests" (17%), mainly set aside for soil protection at exposed sites, for example at the tree line in mountains or watered locations, and "forests with special purposes" (11 %), mainly forests for water retention, recreation or state defence. The protective forests are often not harvested at all, but in case of large-scale natural disturbances such as windstorm or bark beetles the wood is usually salvage logged. Shelter-wood cutting dominates in Slovak forests by approx. 70%, clear-cutting management is applied in 25% of forests. Naturally managed forests cover ca. 1%. The annual harvest rate increased from 6.22 mill m^3 in 2000 to 9.86 mill m^3 at present, and sanitary cutting is responsible for 58% of the harvesting (Moravčík et al. 2019). Forest management in large-scale protected areas was until recently dominated by regular forest management of even-aged forest with pre-set rotation times of ca. 70-110 years in case of Norway spruce and 120-160 years for oak. Forests in protected areas belong to the department of agriculture (State Forest Enterprise) or to private or other public owners (municipalities etc.), but the monitoring and control role fall under the Ministry of the Environment. There is a growing divide between the desire to harvest all the wind-thrown and bark beetle infested trees, and a wish to retain the naturally-disturbed forests without managed. The newest nature conservation law demands close-nature forestry in national parks. Under these new rules, at least 50% of a national park should be un-managed, but his could be hard to achieve given the land ownership – several national parks have mixed ownerships of state and private forests. It is possible that to reach the 50% of unmanaged forests, some national parks will have to be reduced by area. The expectation is that close-nature managed forest will become a conservation instrument adding to the 1% of naturally-managed forests. The first case where the new zonation (50% unmanaged by law) could be applied is the National Park Muranska planina. The Ministry of the Environment calculated that the yearly payment for the exclusion of management would be ca. 1 milion € and 93% of the unmanaged zone would be on state property but there is a backlash against the new zonation among local stakeholders and municipalities (Anonymous 2020).

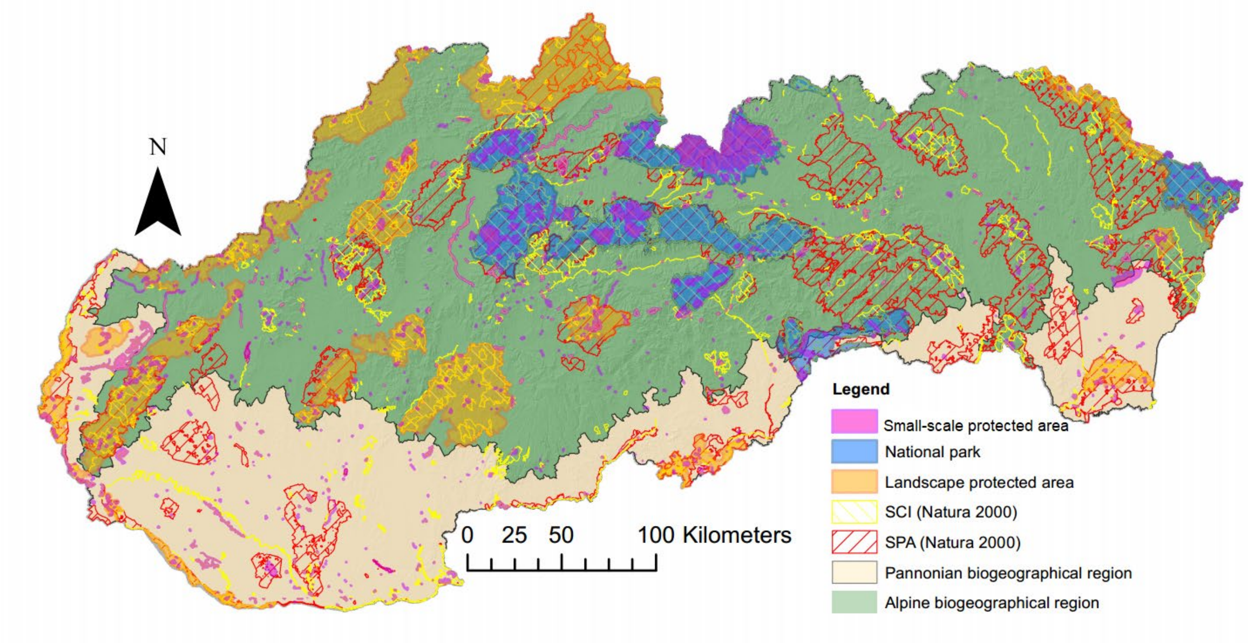

Figure SK1. Biogeographical regions in Slovakia and protected areas (SPA – Birds Directive sites; SCI – Habitat Directive sites).

References

Anonymous. 2018. Nariadenie Vlády Slovenskej republiky č. 75/2015 Z. z. [Government Act No. 75/2015]. Available at: https://www.mpsr.sk/nariadenie-vlady-slovenskej-republiky-c-75-2015-z-z/1098-43-1098-12694/.

Anonymous. 2020. Návrh zvýšenia ochrany Muránskej planiny sa u lesníkov nestretol s pochopením Available at: http://www.sopsr.sk/web/index.php?cl=20625

Butsic, V., Munteanu, C., Griffiths, P., Knorn, J., Radeloff, V.C., Lieskovský, J., Mueller, D. and Kuemmerle, T. 2017. The effect of protected areas on forest disturbance in the Carpathian Mountains 1985–2010. *Conservation Biology* 31:570–580.

Griffiths, P., Kuemmerle, T., Baumann, M., Radeloff, V.C., Abrudan, I. V., Lieskovsky, J., Munteanu, C., Ostapowicz, K. and Hostert, P. 2014). Forest disturbances, forest recovery, and changes in forest types across the Carpathian ecoregion from 1985 to 2010 based on landsat image composites. *Remote Sensing of Environment* 151:72–88.

Kovalčík, M., Tutka, J., Sarvašová, Z., Oravec, M., Moravčík, M., Lásková, J., Schwarz, M., Kovalcík, M., Sarvašová, Z., Schwarz, M., Moravcík, M., Oravec, M., Lásková, J. and Tutka, J. 2012. Financial and socio-economic impacts of nature conservation on forestry in Slovakia. Journal of Forest Science, 58(10), 425–435. https://doi.org/10.17221/14/2012-jfs

Kuemmerle, T., Hostert, P., Radeloff, V.C., Perzanowski, K. and Kruhlov, I. 2007. Post-socialist forest disturbance in the Carpathian border region of Poland, Slovakia, and Ukraine. *Ecological Applications* 17:1279–95.

Lieskovská,Z. and Lényiová, P. 2018. Správa o stave životného prostredia Slovenskej republiky v roku 2018. [Report on the Status of the Environment of the Slovak Republic in 2018.] Zvolen, National Forest Centre, 224 p. Available at: https://www.enviroportal.sk/spravy/detail/9341

Lieskovská, Z. and Považan, R. 2018. Životné prostredie pre všetkých. Slovak Ministry of the Environment, p.438.

Moravčík M., Bednárová, D., M., Kovalčík M., Kunca, A., Longauerová, V., Oravec,M., Rizmanová,E., Sarvašová,Z., Schwarz, M. and Šebeň, V. 2019.. Správa o lesnom hospodárstve v Slovenskej republike 2019. [Report on the Status of Forestry in Slovak Republic 2019.] Zvolen, National Forest Centre, 68 p., available at: https://www.mpsr.sk/zelena-sprava-2019/123---14927/

Mihálik, Š. 1968. Vývoj súčasný stav a perspektíva vytvárania siete chránených území na lesnom fonde. *Československá ochrana prírody* 6:9–15.

Mikoláš, M., Tejkal, M., Kuemmerle, T., Griffiths, P., Svoboda, M., Hlásny, T., Leitão, P.J. and Morrissey, R.C. 2017. Forest management impacts on capercaillie (Tetrao urogallus) habitat distribution and connectivity in the Carpathians. *Landscape Ecology* 32:163–179.

Mikoláš, M.; Ujházy, K.; Jasík, M.; Wiezik, M.; Gallay, I.; Polák, P.; Vysoký, J.; Čiliak, M.; Meigs, G.W.; Svoboda, M.; et al. 2019. Primary forest distribution and representation in a Central European landscape: Results of a large-scale field-based census. *For. Ecol. Manage.* 449:117466.

Sommerfeld, A., Senf, C., Buma, B., D'Amato, A.W., Després, T., Díaz-Hormazábal, I., Fraver, S., Frelich, L.E., Gutiérrez, Á.G., Hart, S.J., Harvey, B.J., He, H.S., Hlásny, T., Holz, A., Kitzberger, T., Kulakowski, D., Lindenmayer, D., Mori, A.S., Müller, J., Paritsis, J., Perry, G.L.W., Stephens, S.L., Svoboda, M., Turner, M.G., Veblen, T.T. and Seidl, R. 2018. Patterns and drivers of recent disturbances across the temperate forest biome. *Nature Communications*, 9.

Sweden (3)

By Per Angelstam

Case study description

Sweden's forest landscapes can be divided into three groups. The first two groups are represented by the two boreal ecoregions, and hemiboreal and nemoral forest ecoregions. All of those focus on high sustained yield wood production based on the two conifers Norway spruce and Scots pine. The third group is the sub-alpine mountain forest region with low productivity. The productive forest area (annual growth rate <$1m^3ha^{-1}$) is 230,000 km^2 and the total forest area is 280,000 km^2.

When it comes to land ownership, Swedish forests are mostly owned by non-industrial private forest owners (49%), private forest industry (23%), and the rest by the National Property Board, the state forest company Sveaskog Co., public bodies such as municipalities and regions, the church and forest commons (28%).

Portfolios of conservation instruments and area proportions (question 1)

The officially acknowledged contributions to the pool of "protected" areas for biodiversity conservation have changed from including only formally protected areas to also voluntary set-asides since the emergence of voluntary forest certification programmes in the 1990s. Since 2018 also retention tree groups on harvested areas and unproductive forests are officially included (Table SE1). The total area proportion of these four instruments is 26% of all forest land. However, in ecoregions used for high yield wood production, the corresponding numbers are 16-30%, and in the sub-alpine ecoregion 72%. There are also considerable differences between the proportions of the total forest area of different kinds of set-asides (Table SE2).

Effectiveness (question 2)

The size, duration, decision-making, control and method for monitoring of the four conservation instruments suggest that they differ in conservation effectiveness (Table SE1). There is a clear decline in the patch size and duration from formally protected areas (>20 ha and permanent) via voluntarily set-asides to nature consideration areas (<ca. 0.5 ha and unknown). For all but the voluntary set-asides decision-making is made by public bodies. The methods for monitoring range from georeferenced GIS data for formally protected areas to questionnaires and random field sampling for the other instruments. Additionally, estimates of the functionality of conservation instruments as green infrastructure (i.e. GI) need to consider also functional connectivity (Figure SE1) and habitat quality. Taking into account these two factors, in ecoregions used for high yield wood production the proportion dropped to 3-8%, and to 54% in the sub-alpine ecoregion (Figure SE1).

Policy implementation tools (question 3)

Carrot: With environmentally friendly markets as customers for wood-based products forest certification schemes have encouraged both voluntary set-asides and green tree retention.

Stick: Formal forest production is permanent and owners receive economic compensation, which is 25% higher that if sold as industrial raw material.

Sermon: Education campaigns, short courses and extension have improved the awareness about the importance of nature conservation and restoration management.

Net effect of conservation and intensification (question4)

Depending on site class productivity, forest management in the surrounding matrix is dominated by even-aged management with rotation times of 50-100 yrs, depending on site class productivity. The stand age distribution has shifted during recent decades to even-aged stands <70 yrs of age. The increase in protected areas is slower than the decline of HVCF areas (e.g., Svensson et al. 2019). The net result of conservation instruments and forestry intensification is thus either limited (in southern Sweden), or negative (in northern Sweden) (see Jonsson et al. (2016) regarding dead wood as an indicator of forest naturalness).

Landscape planning aimed at improving connectivity and representation of different forest types remains a challenge. The large regional variation in the opportunity for landscape planning linked to large variation in forest ownership types stresses the need for a portfolio of different approaches. Where land ownership structure is contiguous (e.g., public and industrial forest owners) landscape planning and restoration can be used. For smallholders, other approaches are needed. Site conditions in terms of soil moisture and nutrient availability, and conservation of cultural forest landscapes are two options to guide forest management bottom-up. There is a need to secure funding mechanisms for compensating land owners' investments in GI, and to adapt both the approaches and spatial extents of landscape planning units to land ownership structure.

The ongoing debate in Sweden is an interesting example on how competing narratives over reality may develop (Mårald et al. 2017; Sténs and Mårald 2020). With terms like bio-economy, a new discourse is beginning to dominate the previous sustainable forest management discourse, which simultaneously considers economic benefits, biodiversity conservation and rural development (Pülzl et al. 2014). Thus, the Swedish Forest Agency (2019) published a report about how wood yield could be further increased by 20%. Simultaneously, there importance for rural development of forestry has declined dramatically due to mechanisation and merging of small inland mills to large units by the Swedish coast. Remaining intact forest landscapes in the mountain region are thus increasingly seen as assets for new value chains supporting rural development based on nature, wilderness and culture (Jonsson et al. 2019, Svensson et al. 2020).

Angelstam and Manton (2021) made an attempt to evaluate the net effects of forestry intensification and conservation on habitat network functionality during the past two decades for biodiversity conservation in the gradient between boreal and mountain forest in Sweden. The net result of the continued transformation of near-natural forest remnants and conservation efforts was negative at the regional and landscape levels, but partly positive at the stand scale. However, at all three scales, habitat amounts were far below critical thresholds for the maintenance of viable populations of species, let alone ecological integrity. With the carrot-stick-sermon analogy for policy implementation, this means that while sermon is too weak, land owners could get paid for biodiversity conservation and carbon storage, or through regulation by the market, the Swedish government, or the EU.

Table SE1. Basic information about four groups of conservation instruments in Sweden (Anon., 2018), including two types of formal protection, voluntary set-aside areas, nature consideration areas and unproductive forests (from Statskontoret (2007: 33) and Skogsstyrelsen (2019a)).

	(i.i) Formal according to the Environmental Code		(i.ii) Formal according to the Land Code	(ii) Voluntary set-asides	(iii) Nature considerations (§ 30, Forestry law)	(iv) Unproductive (<1m3ha-1yr-1) (§ 13a, Forestry law)
Area and proportion of all forest land in 2019	(i.i and i.ii) 2,335 10^3 (8.3%)			(ii) 1,210 10^3 (4.3%)	(iii) 426 10^3 (1.5%)	(iv) 3,239 10^3 (11.5%)
Aim	National park, nature reserve: Conserve and develop nature of high value for plants, animals and people	Biotope protection: Conserve terrestrial or aquatic habitat for threatened species	Conservation agreement: Conserve and develop qualities for biodiversity	A complement to formal protection	Consideration to biodiversity conservation in managed forest	Wood harvest not recommended
Establishment	1909 and 1964, respectively	1998	1993	1995	1979	1979
Target size	Usually >20 ha	Usually <20 ha	Variable	>0.5 ha	< ca 0.5 ha	>0.1 ha
Duration	Permanent	Permanent	Variable	Unknown	Unknown	Permanent
Decision by	Parliament, Government, County, Municipality	Forest Agency, Municipality	Agreement between the State or Municipality and owner	Land owner	Parliament, Government, Forest Agency	Parliament, Government
Control	County	Forest Agency, Municipality	State	Forest certification	Forest Agency	Forest Agency
Monitoring	Georeferenced GIS polygons	Georeferenced GIS polygons	Georeferenced GIS polygons	GIS data and questionnaires	Random field sampling	National Forest Inventory

Table SE2. Proportion of all forest conservation instruments in Sweden, and their effectiveness (Data from Forest Agency 2019).

Unit	Conservation instruments (% of all forest land in each spatial unit)				
	Formal protection	Voluntary protection	Unproductive forest	Tree retention	SUM
Sweden (282,800 km^2)	8.3	4.3	11.5	1.5	25.6
SE-Mountain (31,870 km^2)	45.3	2.6	24.2	0.3	72.4
SE-Boreal (169,700 km^2)	3.6	3.1	21.7	1.6	30
SE-Hemi-boreal (81,200 km^2)	4.0	5.2	5.0	1.5	15.7

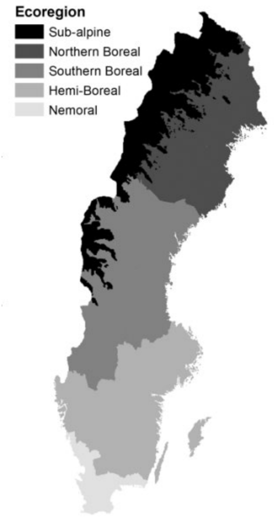

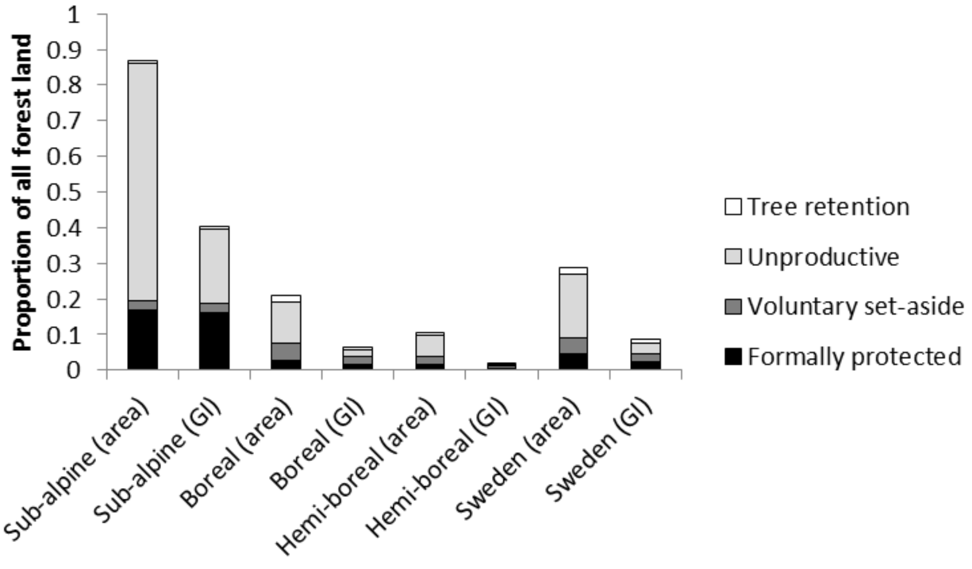

Figure SE1. Sweden has two coarse forest ecoregions with a focus on maximum sustained yield wood production, plus sub-alpine mountain forests with low productivity. There are four official categories of set-asides (formally protected, voluntary set-aside, unproductive plus retention trees within harvested stands). The figure illustrates two aspects of these four types of set-asides as components of functional green infrastructure. The first is the large difference between the north boreal mountains forests of little economic interest for intensive wood production versus the other regions focusing on maximum sustained yield forestry. The second is the considerable difference between the proportion of the total forest area of different kinds of set-asides (i.e. area), and estimates of the functionality as green infrastructure (i.e. GI) for biodiversity conservation by considering the role of the connectivity (Angelstam et al. 2020), and an estimate

of the lower biodiversity value in unproductive forest (e.g., Hämäläinen et al. 2018). Data about the different kinds of set-asides are from Skogsstyrelsen (2019).

To conclude, even if 31% of forest land in Sweden is officially protected, voluntarily set-aside, or not used for wood production now and in the future, Angelstam et al. (2020) show that applying the representation and connectivity criteria of Aichi target #11 reduces this figure to an effective GI of 12%. When disaggregating the five ecoregions the effective GI was 54% for the sub-alpine forest ecoregion, which hosts EU's last intact forest landscapes, but only 3-8% in the other ecoregions where wood production is predominant. This results in an increasing need for forest habitat and landscape restoration from north to south.

References

Angelstam, P. and Manton, M. 2021. Effects of forestry intensification and conservation on green infrastructures: A spatio-temporal evaluation in Sweden. *Land* 10:531. https://doi.org/10.3390/land10050531

Angelstam. P., Manton, M., Green, M., Jonsson, B.-G., Mikusinski, G., Svensson, J. and Sabatini, F. M. 2020. High Conservation Value Forest remnants in Sweden do not satisfy agreed biodiversity targets: a call for adaptive landscape planning. *Landscape and Urban Planning* 202: 103838. https://doi.org/10.1016/j.landurbplan.2020.103838

Felton, A., Löfroth, T., Angelstam, P., Gustafsson, L., Hjältén, J., Felton, A. M., Simonsson, P., Dahlberg A., Lindbladh, M., Svensson, J., Nilsson, U., Lodin, I., Hedwall, P.O., Sténs, A., Lämås, T., Brunet, J., Kalén, C., Kriström, B., Gemmel, P. and Ranius, T. 2019. Keeping pace with forestry: Multi-scale conservation in a changing forest matrix. *Ambio* 49:1050–1064. https://doi.org/10.1007/s13280-019-01248-0

Jonsson, B. G., Ekström, M., Esseen, P.-A., Grafström, A., Ståhl, G. and Westerlund, B. 2016. Dead wood availability in managed Swedish forests – Policy outcomes and implications for biodiversity. *Forest Ecology and Management* 376:174-182. doi: https://doi.org/10.1016/j.foreco.2016.06.017

Jonsson, B.G., Svensson, J., Mikusiński, G., Manton, M. and Angelstam, P. 2019. European Union's last intact forest landscape is at a value chain crossroad between multiple use and intensified wood production. *Forests* 10(7):564; https://doi.org/10.3390/f10070564 (https://www.mdpi.com/1999-4907/10/7/564)

Hämäläinen, A., Strengbom, J. and Ranius, T. 2018. Conservation value of low-productivity forests measured as the amount and diversity of dead wood and saproxylic beetles. *Ecological Applications* 28(4):1011-1019. doi: 10.1002/eap.1705

Mårald, E., Sandström, C. and Nordin, A. 2017. Forest governance and management across time: developing a new forest social contract. London: Routledge.

Sténs, A. and Mårald, E. 2020. "Forest property rights under attack": Actors, networks and claims about forest ownership in the Swedish press 2014–2017. *Forest Policy and Economics* 111:102038.

Svensson, J., Andersson, J., Sandström, P., Mikusiński, G. and Jonsson, B. G. 2019. Landscape trajectory of natural boreal forest loss as an impediment to green infrastructure. Conservation Biology, 33(1), 152-163. doi: 10.1111/cobi.13148

Svensson, J., Neumann, W., Bjärstig, T., Zachrisson, A. and Thellbro, C. 2020. Landscape Approaches to Sustainability—Aspects of Conflict, Integration, and Synergy in National Public Land-Use Interests. *Sustainability* 12(12):5113.

Ukraine (9)

By Mariia Fedoriak and Taras Yamelynets

Case study description

Considering the large territory (603,628 km^2) and great variations in physiographic conditions, Ukraine has territories in zonal ecoregions linked to latitude (Continental, Steppic, and Pannonian) and azonal ones linked to altitude (Alpine) (EEA 2019). According to the National Atlas of Ukraine (2007) there are six ecoregions: the mountain region in the Ukrainian Carpathians, the mixed forest zone; the deciduous forest zone; the forest-steppe zone, the steppe zone, and the Crimean mountain region. Except the steppe all these ecoregions have forest as the natural potential vegetation. The total forest area within these five ecoregions is about 6.51 million ha (10.8%). The forests of Ukraine are formed by more than 30 tree species dominated by Scots pine (*Pinus silvestris*), oak (*Quercus robur*), beech (*Fagus sylvatica*), Norway spruce (*Picea abies*), birch (*Betula pendula*), alder (*Alnus glutinosa*), ash (*Fraxinus excelsior*), hornbeam (*Carpinus betulus*) and fir (*Abies alba*). Coniferous forests make up 43% of the total forest area, of which pine constitues 35%-units, and deciduous forest make up 57%, of which oak and beech form 37% (official data of State Forestry Agency, http://dklg.kmu.gov.ua). All forests are owned by state organisations.

Portfolios of conservation instruments and area proportions (question 1)

Formal protection in Ukraine is linked to the Emerald network[5] which is an ecological network made up of Areas of Special Conservation Interest under the Bern Convention. Currently, 377 officially adopted Emerald sites are listed for Ukraine (Updated list, 2019). In 2019 the Nature Conservation Fund of Ukraine was 8295 territories and sites with total area of 3.98 million ha (6.61%) within the territory of Ukraine (Figure UA 1, Table UA 1).

Institutions of natural reserve fund of Ukraine are under authority of the Ministry of Energy and Environment Protection. Additionally, the State Forest Agency is managing 13 protected areas (total area about 0.2 million ha), including: 6 nature reserves, 6 national nature parks and one farm-park. Finally, through the process of introducing forest certification via FSC the officially acknowledged contributions to the pool of protected areas in Ukraine have changed from including only formally protected areas to also voluntary protected forests. The total area of all FSC-certified forests in Ukraine is about 4.51 million ha, which is 43% of all forest lands in the country. In ecoregions used for high yield wood production, the corresponding numbers are 55-70%, while in the forest steppe ecoregion it is only 25%. However, the Ukrainian FSC standard does not prescribe quantitative targets neither for voluntary set-asides nor retention trees (see below[6],[7]).

---

[5]Updated list of officially adopted Emerald sites (December 2019) T-PVS/PA(2019)17 CONVENTION ON THE CONSERVATION OF EUROPEAN WILDLIFE AND NATURAL HABITATS. Strasbourg, 6 December 2019 available online. https://rm.coe.int/updated-list-of-officially-adopted-emerald-sites-december-2019-/168098ef51?fbclid=IwAR3Sfh-F_w0fpHBkCggkU1Xc1bUbo57vMgDhu1Fcgq-gFvM5QaceWsnOlt4

[6] THE FSC NATIONAL FOREST STEWARDSHIP STANDARD OF UKRAINE, 2019:
For the purposes of Principle 9, HCVs 1, 2 and 6 there are three main forms of recognizing significance.
- A designation, classification or recognized conservation status, assigned by an international agency such as IUCN or Birdlife International;
- A designation by national or regional authorities, or by a responsible national conservation organization, on the basis of its concentration of biodiversity;

Since independence (1991) Ukrainian laws “On Nature and Reserve Fund” (1992) and “On Ecological Network” (2004) form the base for further development protected areas. Actions related to increase of ecological network areas in accordance with the National Programme for Development of National Ecological Network for 2000-2015 are being implemented. As of 2013 the area of lands of ecological network is 23.0 million ha corresponding to 38.16% of the total country area. The decree of the President of Ukraine “On Additional Measures for the Development of Forestry, Rational Use of Nature and Preservation of Objects of the Nature Reserve Fund” was signed in 2017. However, its professionalism and feasibility have been questioned.

Effectiveness (question 2)

Four factors affect the effectiveness of protected areas in Ukraine as a functional green infrastructure. First, due to a long history of forest clearance for agriculture, ecoregional representativeness is biased towards less productive land (Figure UA 2). Second, the relatively low forest cover means that, except for the Carpathian Mountains, the level of forest fragmentation is high (Angelstam et al. 2017). Third, the managed matrix is dominated by young forests. The average age of trees is about 60 years, and the age distribution is dominated by middle age stands (45%). Mature and old growth forests cover of 17%. Fourth, forest management intensity is increasing. Over the past 50 years, the forest cover of Ukraine has increased from 12 to 17 % (Tkach 2012), which implies a current domination of plantations and younger stands. Timber stock is estimated at about 2.1 10^9 m^3. The total wood stock over the last 50 years has increased 3-fold (Tkach 2012). The average annual yield wood production is to 3.9 m^3 ha^{-1} yr^{-1} and in the mountain ecoregion has increased to 5.0 m^3 ha^{-1} yr^{-1} (https://www.foresteurope.org/docs/implementation/IRUkraine02.pdf). Finally, organizational and legal mechanisms of eco-network formation are imperfect, and there is no orderly management structure in the field of eco-network formation, conservation and use (Даниленко 2013). The lack of clearly defined boundaries of national parks, nature reserves and other territories in practice leads to taking the territories by officials to private ownership (Stativka, 2018).

Policy implementation tools (question 3)

Carrot: Confirmation of responsible forestry through obtaining an FSC certificate is not just a market requirement, but also part of the national legislation. According to the Association Agreement between Ukraine and the European Union Ukraine is obliged to provide legal trade and sustainable forest management. In general, it means compliance with the requirements of the FSC standards. In other words, voluntary certification is expected not only provide sustainable forestry, but also to obtain economic benefits.

- A voluntary recognition by the manager, owner or The Organization, on the basis of available information, or of the known or suspected presence of a significant biodiversity concentration, even when not officially designated by other agencies.

[7] Reply from FSC Ukraine, Pavlo Kravets: “We do not collect such information centrally at this time. It is therefore necessary to download public holders' reports and retrieve HCVs information from the info.fsc.org database. It is there. The difficulty is that the area of HCVs consists of both existing Nature Conservation Fund (NCF) sites and those that are voluntarily allocated and protected. Accordingly, you can find out about it only by applying directly to the forestry or find the difference within each category. But the last path may contain a considerable amount of mistakes, assumptions and inaccuracies”.

Stick: The number of policy instruments to deal with forest conservation and management has increased substantially during recent years. This has happened mainly as a result of the signed Association Agreement between Ukraine and European Union what means adaptation of national legislation to the European Directive No 92/43 / EC on the conservation of natural habitats, wildlife fauna and flora, as amended by Directives No 97/62 / EC, 2006/105 / EC and Regulation (EU) № 1882/2003. Penalties for illegal felling of trees or taking from the wild of the Red Data Book species have been substantially elevated. Protected areas, which cover about one third of OGF in the Ukraine Carpathians, were found to be effective in reducing disturbance with annual disturbance rates 7–8 times lower than in OGF outside protected areas (Spracklen and Spracklen 2020).
Sermon: Education campaigns, short courses and extension have improved the awareness about the importance of nature conservation and restoration management. Church may play important role in promotion of the conservation ideas, especially in rural areas.

Net effect of conservation and intensification (question 4)

The national conservation targets are designated in the law of Ukraine "On the Fundamental Principles (Strategy) of the State Environmental Policy of Ukraine for the Period up to 2030" adopted in February 2019. According to the law, the land area of PAs in Ukraine for 2020 should be 10.4 % of the total territory of the country, for 2025 – 12.5% and for 2030 – 15%. Therefore, despite the progress made in April 2019, there is a need at the state level to step up efforts to achieve this target.

The State Forestry Program of Ukraine for 2016-2020 stated that at least 2 million hectares of new forests should be created in order to achieve an optimum forest cover of ca 20%. The main unresolved environmental problem is illegal logging, e.g. in 2015 the volume of officially registered illegal logging in Ukraine amounted to 24.1 10^3 m^3, but in reality this number is much bigger (Nekos et al. 2015).

According to the Law of Ukraine "On the Fundamental Principles (Strategy) of the State Environmental Policy of Ukraine until 2030" the main problems in the forest sector are the imperfection of the forest management system, the lack of legal and economic mechanisms to achieve policies, the complicated introduction of environmental technologies, the ineffective tax system, as well as the unclear definition of the legal status of land management under the forest protection massifs and strips among the agriculture fields. About 0.8 million hectares of state-owned forest land (including forest protection massifs and strips among the agriculture fields) are not used for wood production, and is classified as reserve land. The unclear definition of the legal status of forest protection massifs and strips among the agriculture fields leads to a significant deterioration of the forest status and their loss.

Table UA1. Proportion of all forest conservation instruments in Ukraine (Data from State Forest Agency, 2018; and own GIS analyses).

	**Area of nature conservation fund PAs, (km^2)**	**Area of PAs, % of Ukraine's territory**	**Formally protected forests,% of forest areas**	**Voluntary protection (FSC), %**
Ukraine (603,628 km^2) (forest=104,000 km^2)	39,842	6.61	10.34	Not available
1. UA-Mountain Carpathians (38,363 km^2) (forest=17,236 km^2)	4,468	11.64	19.22	Not available
2. UA-Temperate zone of mixed forests (Polissia) (100,773 km^2) (forest=33,931 km^2)	9,095	9.03	11.86	Not available
3. UA-Temperate zone of deciduous forest (53,050 km^2) (forest=2,982 km^2)	2,745	5.17	19.98	Not available
4.UA-Forest steppe (168,814 km^2) (forest=8,647 km^2)	3,447	2.04	10.30	Not available
5 Crimea mountain (8,061 km^2) (forest=2,278 km^2)	998	12.38	28.62	Not available

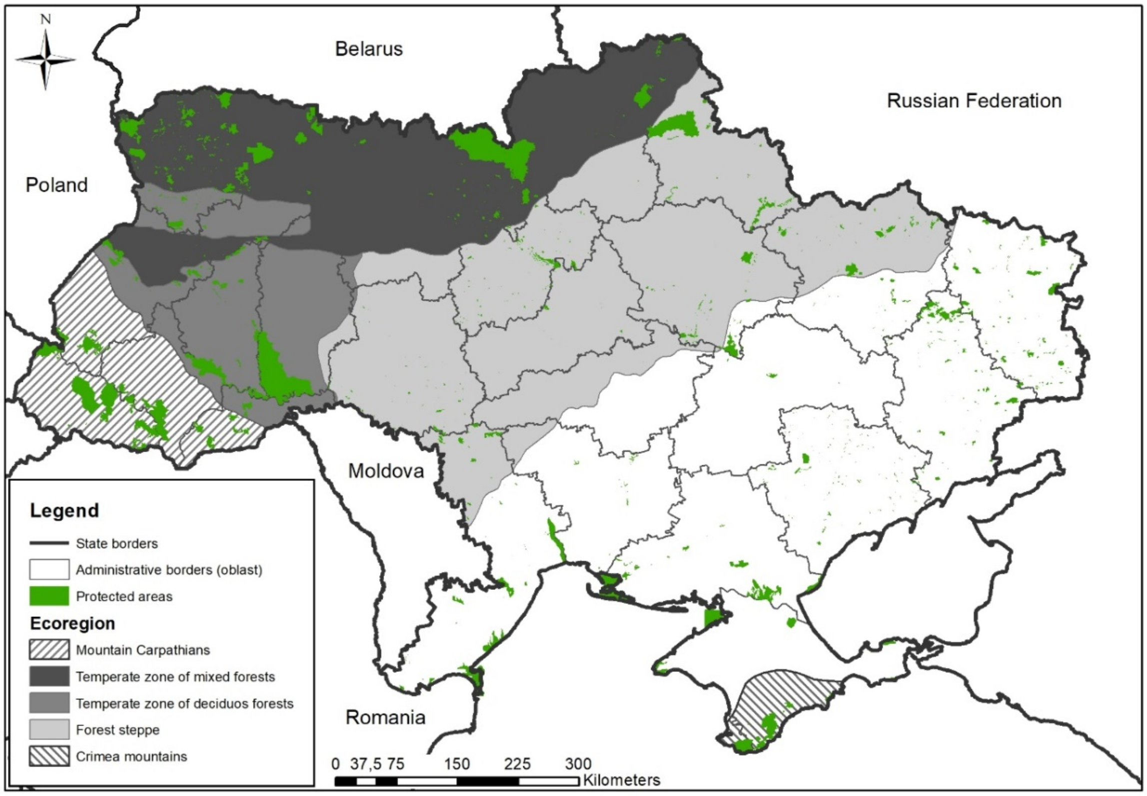

Figure UA1. Ecoregions of Ukraine (Marynych at al. 2003) and the distribution of protected areas

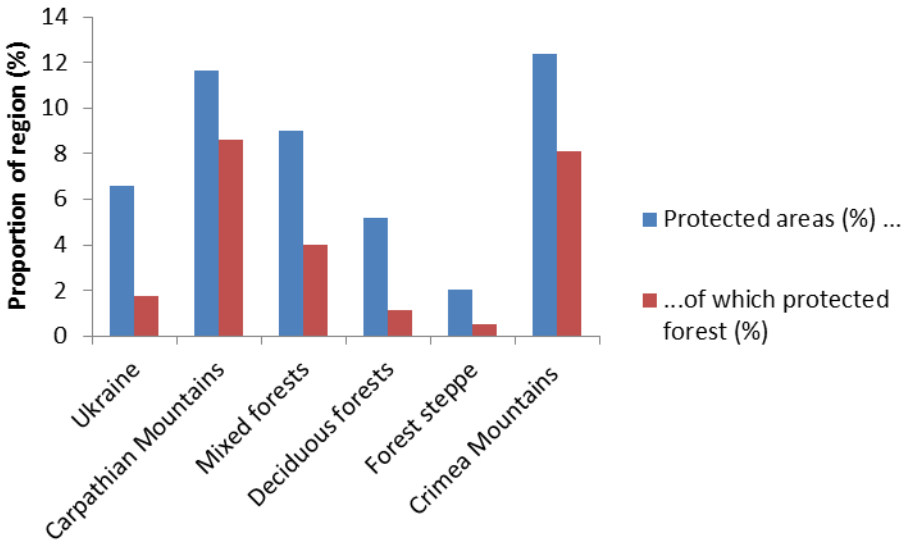

Figure UA2. Ecoregional variation in protected areas and the relative role of forests in them in Ukraine.

References

Angelstam, P., Yamelynets, T, Elbakidze, M., Prots, B. and Manton, M. 2017. Gap analysis as a basis for strategic spatial planning of green infrastructure: a case study in the Ukrainian Carpathians. Écoscience 24 (1-2): 41-58.

Anon. Undated. Fifth national report of Ukraine to the Convention on Biological Diversity (https://www.cbd.int/doc/world/ua/ua-nr-05-en.pdf)

Antonenko I. Y. 2001. Economic support for the protection, reproduction and use of forest resources of Ukraine / Economics of nature management and environmental protection: Coll. of materials. Kyiv. 210 p. (in Ukrainian)

Khvesyk, M.A., Shubalyi, O.M., Khvesyk, J.M. and Vasilik, N.M. 2019. Conceptual basis of transformation of ecological and economic relations in the forest sector of Ukraine in the context of European integration. Folia Forestalia Polonica, 61(2), pp.97-111.

Marynych, O., Parkhomenko, G., Petrenko, O. and Shyshchenko, P. 2003. Revised scheme of physical-geography zonation of Ukraine. Ukrainian Geography Journal 2:6-20. (in Ukrainian)

Nekos, A.N. and Rego, M.Z. 2015. Ecological value of forests and principles of effective conservation and reproduction of forest resources / Man and the environment. Problems of neoecology3-4: 55-60. (in Ukrainian)

Svyntuh, M. 2016. Legal regulation of Forest land fund. Ukrainian journal of applied economy 1(1):163–170. URL: http://ujae.tneu.edu.ua/index.php/ujae/article/view/17

Shershun, S. 2005. Ecological legal regulations of forestry in Ukraine: Ph.D. thesis of Law Science : 12.00.06. Kyiv. 212 p. (in Ukrainian)

Spracklen, B. D. and Spracklen, D. V. 2020. Old-Growth Forest Disturbance in the Ukrainian Carpathians. *Forests* 11(2):151.

State Forest Agency of Ukraine. Official web-page (2020): http://dklg.kmu.gov.ua/

Stativka, O. O. 2018. Legal regime of the Nature Reserve Fund as an integral part of the ecological network of Ukraine. Dissertation for obtaining a scientific degree of the Candidate of Legal Sciences. Yaroslav Mudryi National Law University, Ministry of Education and Science of Ukraine, Kharkiv. (in Ukrainian)

Tkach, V. 2012. Forest and forest cover in Ukraine: actual state and perspectives of development. Ukrainian Geography Journal 2:49-55. (in Ukrainian)

THE FSC NATIONAL FOREST STEWARDSHIP STANDARD OF UKRAINE, 2019. FSC-STD-UKR-01-2019 V 1-0. https://fsc.org/en/document-centre/documents/resource/428

National FSC office in Ukraine. 2020. Official report: Facts and Figures FSC UA (01.03.2020). available online. https://ua.fsc.org/

Concept of the State Program for Forestry Development of Ukraine for 2016-2020 [Electronic resource]: Project for discussion - Mode of access to the resource: http://dklg.kmu.gov.ua/forest/control/uk/publish/article?art_id=113516&cat_id=82872

Law of Ukraine "On the Fundamental Principles (Strategy) of the State Environmental Policy of Ukraine for the Period up to 2030". February 28, 2019 № 2697-VIII

Даниленко Б. В. 2013. Створення екологічних коридорів в Україні: земельноправовий аспект. Вісник Академії правових наук України. Харків: Право1(72):188

Nova Scotia (10)

By Malcolm Hunter and Robert Seymour

Case study description

Nova Scotia is the southeastern-most of Canada's 10 provinces and three territories. Forests cover about 75% of its 55,284 km^2 area. Non-forested native vegetation and aquatic ecosystems comprise about 15%; and almost 10% of the area is dominated by human uses such as agricultural and urban areas (novascotia.ca/natr/forestry/reports/State_of_the_Forest_2016.pdf).

Approximately 60% of the province is privately owned; small woodlot owners hold about 2/3rds of the private forests and the rest are owned by mill owners and those with holdings over 400 ha. Almost all of the public lands are managed by the province (~34% of land, 37% of forests); federal lands are only about 3% of the total.

Nova Scotia's forests are primarily in the Acadian (or hemiboreal) forest region that stretches westward through New Brunswick, Prince Edward Island, northern New England and northeastern New York (Loo and Ives 2003). It has a cool, relatively humid climate with precipitation evenly distributed over the year. Importantly, abundant rainfall during the growing season limits stand-replacing fires. The original pre-settlement forests were dominated by late-successional species: notably conifers such as red spruce (*Picea rubens*) (whose range largely coincides with the Acadian forest), eastern white pine (*Pinus strobus*), eastern hemlock (*Tsuga canadensis*), balsam fir (*Abies balsamea*) and deciduous hardwoods such as sugar maple (*Acer saccharum*), American beech (*Fagus grandifolia*), yellow birch (*Betula alleghaniensis*), and red maple (*Acer rubrum*). The region was entirely glaciated during the Pleistocene, leaving soils that are mostly tills with some outwash, marine sediments, and organic bogs. Europeans colonized the region in the early 17th century, with extensive areas cleared for agriculture that subsequently have reverted to forest. However, vast areas were never settled and have remained forested, but subject to repeated cutting for wood products.

Natural disturbance regimes are dominated by small-scale gap dynamics. Stand-replacing events are quite uncommon, with typical return intervals of many centuries to millennia (Fraver et al. 2009; Lorimer and White 2003; Seymour et al. 2002; Taylor et al. 2020). This is probably the key feature that distinguishes the Acadian forest region from the fire-prone boreal forests that dominate most of Canada. Even-aged systems are widely practiced for production forestry, but have been criticized for "borealizing" forest composition in favour of early succession forests dominated by shade-intolerant hardwoods (aspen, paper birch), along with the white and black spruce that dominate boreal forests (Noseworthy and Beckley 2020). "Ecological" forestry is also becoming more common on non-industrial lands; it is characterized by multi-aged stand structures maintained by partial harvesting, mainly variants of the irregular shelterwood system (Raymond and Bedard 2017; McGrath in revision).

Portfolios of conservation instruments and area proportions (question 1)

In 2019 the province officially accepted a "Triad" model to forest management that seeks to balance ecological and socio-economic goals by designing and managing forest landscapes (Seymour and Hunter 1992, 1999). In the "Triad" model, three basic approaches to management are recognized: 1) reserves in which forests are set aside from commercial extraction; 2) commodity production areas where growing timber is emphasized; and 3) a dominant matrix

where ecological forestry is practiced. The fundamental idea is that there is no single right way to manage (or not manage) forests, and that thoughtful, cooperative design of forest landscapes can both conserve native biodiversity and sustain forest-based economies as a base for rural and regional development. This concept that all reasonable forest uses have merit has proven to be a valuable vehicle to move beyond long-standing, polarized "preservation versus utilization" arguments that have fostered only conflict, not resolution. Implementation of the "Triad" model is currently underway on crown lands and there is also discussion of mechanisms to foster this approach on private lands as well.

The reserve portion of the "Triad" is easiest to define: 12.6% of the province's total area has been taken out of timber production, chiefly in provincial Wilderness Areas, but also in National Parks and provincial Nature Reserves & Parks and some private conservation areas. Canada supports meeting the CBD Aichi targets of 17% protection so the area of reserves is likely to increase a bit more (Table NS1); 13% protection is the current goal for the province.

Table NS1. Conservation instruments and their areas in Nova Scotia, Canada, expressed as proportions of the whole province which has an area of 55,284 km^2.

	Formal government protection (0.123 collectively)			Other conservation		Unprotected Forest**
Nova Scotia forests	National Parks	Provincial Nature Reserves & Parks	Provincial Wilderness Areas	Private protected areas	Provincial forests with little or no logging*	
0.75	0.025	0.007	0.091	0.003	0.045	0.58

*Tabulated here are old forests, watercourse buffers, wildlife habitat buffers and zones, and rare and sensitive ecosystems in which logging is prohibited or strictly regulated, but not non-forested ecosystems and forests that are not commercially exploitable.
** This figure includes private forests in which logging is constrained by green certification programs or environmental regulations, most notably 1700 km^2 of watercourse buffers.

Crown lands are leased for timber production to private licensees but the province has detailed operational requirements and must approve all operations. A plan under development will allocate a significant portion of the remaining, not-reserved crown land (11,950 km^2) to high production forests in which all the tools of silviculture (planting, clearcutting, etc.) are encouraged. Sites totalling 3,330 km^2 have been identified as potentially suitable for high production forestry, but the final allocation will be lower than that (https://novascotia.ca/ecological-forestry/high-production-forestry/docs/high-production-forestry-%20discussion-paper.pdf). The remaining unreserved crown land will either: 1) not be logged (because of ecological values or because of a dearth of trees suitable for management); or 2) will be managed under an ecological forestry regime that emulates natural disturbance regimes. In the Acadian forests, this will entail adoption of multi-aged silvicultural systems (irregular shelterwood-cutting mostly); in the Maritime Boreal region of Cape Breton, two-aged systems with significant retention of reserve trees will be employed.

Effectiveness (question 2)

It seems likely that the strategy outlined here will succeed in maintaining Nova Scotia's ecological integrity because the province enjoys a good situation (compared to many parts of the Earth) at two ends of the continuum of human use of ecosystems. On the one end only about 10% of the province's ecosystems have been completely converted to human non-forest use leaving ~90% in some approximation of native ecosystems (compromised primarily by periodic logging, in some places by conversion to agriculture, then recovery). This means that overall connectivity of forest land is relatively intact because fragmentation has been in the form of dissection by roads, not extensive conversion, in most regions of the province. (The Annapolis Valley with about 53% conversion to agriculture and urban use is the most notable exception). Protected areas are reasonably well distributed geographically and the province has a detailed vegetation classification that was instrumental in designing the protected area system (https://novascotia.ca/nse/protectedareas/ecoframe.asp).

Table NS2. Distribution of "Triad" management regimes in Nova Scotia, as specific proportions of 55,284 km^2 or as goals for change. The generic goal of increasing allocations to all three forms of "Triad" management reflects a commitment to move toward strategic landscape level planning. There is a missing column here, representing a goal of *decreasing* short rotation, even-aged management with little investment in increasing commodity production.

Categories of land	"Triad" category		
	Reserves	High production	Ecological matrix
Nova Scotia (55,284 km^2)	0.125	increase	increase
Large private forests (9,420 km^2)	increase	increase	increase
Smallholder forests (18,920 km^2)	increase	increase	increase
Provincial land (18,540 km^2)	0.1	0.06 *maximum*	0.16 *minimum*
Federal land (1,387 km^2)	0.025	None	None

Policy implementation tools (question 3)

Sticks: Negotiating and enforcing the terms for leasing crown forests can constitute a stick but on private forests ownership rights are strong, and the only sticks are regulations that protect public resources such as water and wildlife.

Carrots: There is discussion of shifting silvicultural subsidies away from even-aged production practices (e.g., planting, control of vegetation that competes with regeneration) to supporting the higher logging costs associated with ecological forestry such as tree marking and harvest layout, but this is still at an early stage. Green certification is fairly common with FSC certification for some crown land and one large industrial owner, Port Hawkesbury Paper.

Sermon: Developing a triad approach to management of provincially owned forests is well underway but no specific program has been promulgated to encourage thousands of private forest landowners to follow this path.

Net effect of conservation and intensification (question 4)

The simple goal of a "Triad" strategy is to compensate for any timber supply losses resulting from establishing reserves by increasing production from high-intensity silviculture. Additionally, shifting to multi-aged silviculture with high levels of retention will likely have short- to medium-term negative impacts on wood supply, but in the longer term, increased yields from the high-production forests should more than offset these losses. While short rotation, even-aged management will continue, with its consequences for "borealization" of stands, it is hoped that it will have a diminished role in future forest landscapes.

Works Cited:

Fraver, S., White, A.S. and Seymour, R.S. 2009. Patterns of natural disturbance in an old-growth landscape of northern Maine, USA. *Journal of Ecology* 97: 289–298.

Loo, J. and Ives, N. 2003. The Acadian forest: Historical condition and human impacts. *Forestry Chronicle* 79(3):462-474.

Lorimer, C. G. and A.S. White. 2003. Scale and frequency of natural disturbances in the northeastern US: implications for early successional habitats and regional age distributions. *Forest Ecology and Management* 185:41-64.

McGrath, T. In revision. Nova Scotia Silviculture Guide for the Ecological Matrix. Nova Scotia Dept. Lands and Forestry, Truro, NS.

Noseworthy, J. and Beckley, T.M. 2020. Borealization of the New England-Acadian forest: A review of the evidence. *Environmental Reviews* https://doi.org/10.1139/er-2019-0068

Raymond, P., and Bedard, S. 2017. The irregular shelterwood system as an alternative to clearcutting to achieve compositional and structural objectives in temperate mixedwood stands. *Forest Ecology and Management* 398:91-100.

Seymour, R. and Hunter Jr., M.L, 1999. Principles of ecological forestry. Pages 22-61 in Hunter, M.L. (ed.). Maintaining biodiversity in forest ecosystems. Cambridge University Press, Cambridge, England. 698pp.

Seymour, R. and Hunter, Jr., M.L. 1992. Principles and applications of New Forestry in spruce-fir forests of eastern North America. Maine Agricultural Experiment Station Miscellaneous Publication 716

Seymour, R.S., White, A.S. and deMaynadier, P.G. 2002. Natural disturbance regimes in northeastern North America – Evaluating silvicultural systems using natural scales and frequencies. *Forest Ecology and Management* 155:357-367.

Taylor, A.R., MacLean, D.A., Neily, P.D., Stewart, B., Quigley, E., Basquill, S.P., Boone, C.K., Gilby, D., and Pulsifer, M. 2020. A review of natural disturbances to inform implementation of ecological forestry in Nova Scotia, Canada. *Environmental Reviews* https://doi.org/10.1139/er-2020-0015

Costa Rica (11)

By Carlos L. Muñoz Brenes

Case study description

Costa Rica extends 51,100 km^2 with many vegetation types associated with variation in elevation, geomorphology, temperature, and precipitation patterns. According to Holdridge (1967) forests include Dry Forest in the Pacific lowlands, Subalpine Rain Paramo, to Tropical Wet Forest in the Caribbean lowlands (Janzen, 1983). Recent studies of forest cover show a decrease from 59.5% in 1960 to 40.8% in the 1980s, and with current cover of 51.4 % (Calvo-Obando and Ortiz-Malavasi 2012; Sánchez-Azofeifa 2015). Estimates by forest type indicate an area of 6,230 km^2 (12.2%) as primary forest, 17,410 km^2 (34.1%) of naturally regenerated forest, and 2,410 km^2 (4.7%) of planted forest, with 45% in public ownership overall all types (FAO, 2010). Costa Rica is well known for its rich biodiversity with 11,467 species of plants (2,000 species of trees), 189 amphibians, 854 birds, and 237 mammals (Kappelle, 2016).

Portfolios of conservation instruments and area proportions (question 1)

All protected areas (PAs) owned by the State are part of the National System of Protected Areas, which assigns management categories with varying levels of restrictions to land use and access to their natural resources. The total area under this system has increased over the years and reached 33% (17,000 km^2) of the country's territory in 2018. Forest protection is marginally increased by 2% (820 km^2) in private PAs and less than 1% (470 km^2) under payment for ecosystem services (Figure CR1).

Effectiveness (question 2)

Conservation approaches are limited by socio-political opposition to land expropriation or use restriction and government financial constraints. For example, policy-makers must make trade-offs in using limited resources for reducing poverty or expanding conservation. Thus, demonstrating that conservation investments are effective and do not exacerbate poverty is key. Several impact evaluations demonstrate how conservation efforts are effective at reducing deforestation (Hanauer & Canavire-Bacarreza, 2015; Robalino & Pfaff, 2013) while helping alleviate poverty (Paul J. Ferraro & Hanauer, 2011), and that conservation aligns with other larger societal benefits (P. J. Ferraro et al., 2013; Paul J. Ferraro, Hanauer, & Sims, 2011; Robalino & Pfaff, 2013; Robalino, Sandoval, Barton, Chacon, & Pfaff, 2015).

Policy implementation tools (question 3)

Carrot: There is a sense of national pride about conservation in Costa Rica for its contributions to protecting biodiversity and improving human well-being. Conservation is linked to the economics of tourism with annual increasing visitation to both State and privately protected areas (Figure CR2) (PEN-CONARE, 2018). In addition to the biodiversity values, water provisioning from protected sites is a positive spillover effect from conservation interventions, mainly for hydroelectric power generations, human consumption, and irrigation systems (Bernard, de Groot, & Campos, 2009).

Stick: While forest production is not a major driver of deforestation in Costa Rica, the national legal system (e.g., 1996 Forest Law) imposes land use restriction on private lands to avoid forest conversion. However, there are economic incentives that drive deforestation, mainly from agricultural activities (Fagan et al., 2013; Jones, 2003; PEN-CONARE, 2018).

Sermon: Conservation is expensive and hard to enforce. Nonetheless, forest protection creates opportunities for the country's ambitions around connectivity through voluntary schemes like biological corridors or climate change mitigation and adaptation goals. Thus, the government has new opportunities to justify conservation expansion and investment to improve the management capacity of the national system of PAs.

Net effect of conservation and intensification (question 4)

Costa Rica is an interesting natural experiment that shows how conservation approaches evolved from restrictive state-driven measures to more flexible mechanisms. Still, the country struggles with limited funding against the high costs of conservation and the opportunity costs associated to land use restrictions and state regulations to avoid ecosystem services degradation and pressures from a growing economy and human demands. Further studies are needed to understand conservation approaches effectiveness to deliver habitat quality, representativeness, connectivity, and climate change benefits that have not been realized (Paul J. Ferraro, 2011).

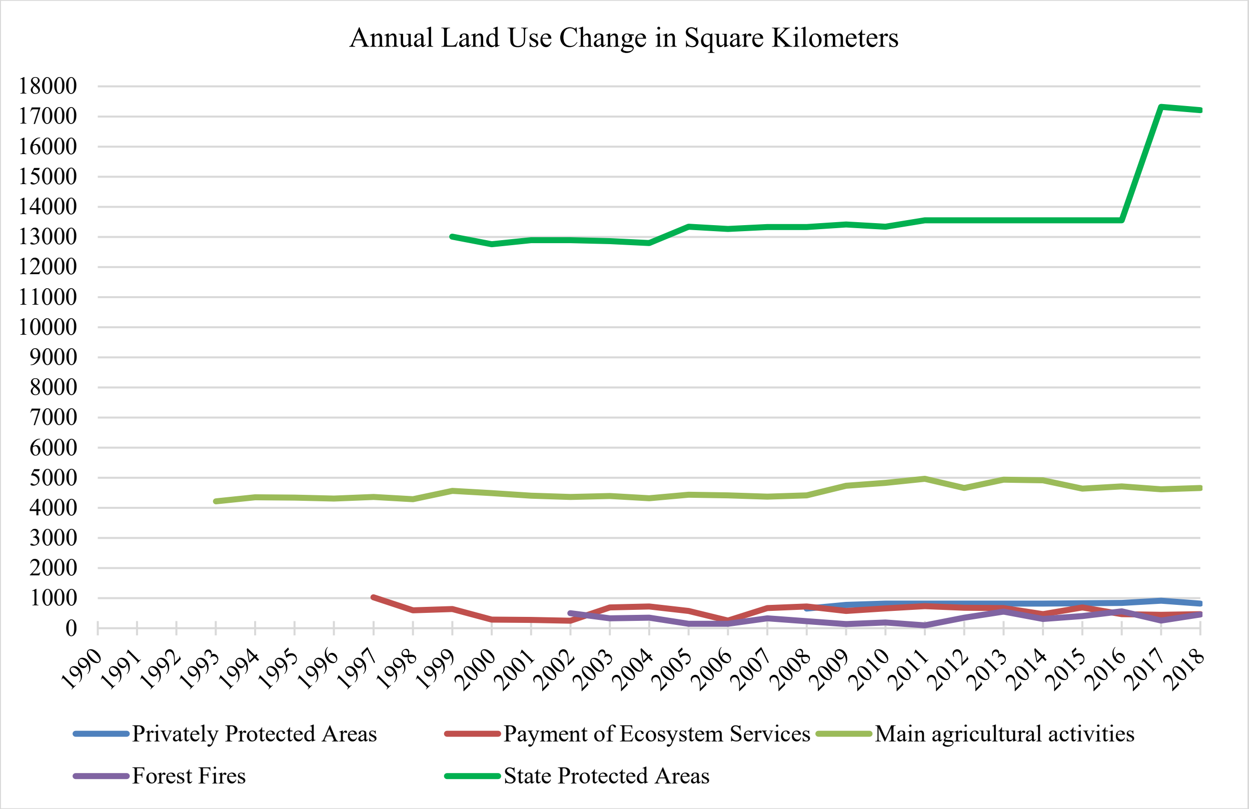

Figure CR1. Area coverage in km^2 by land use type in Costa Rica from 1990 to 2018.

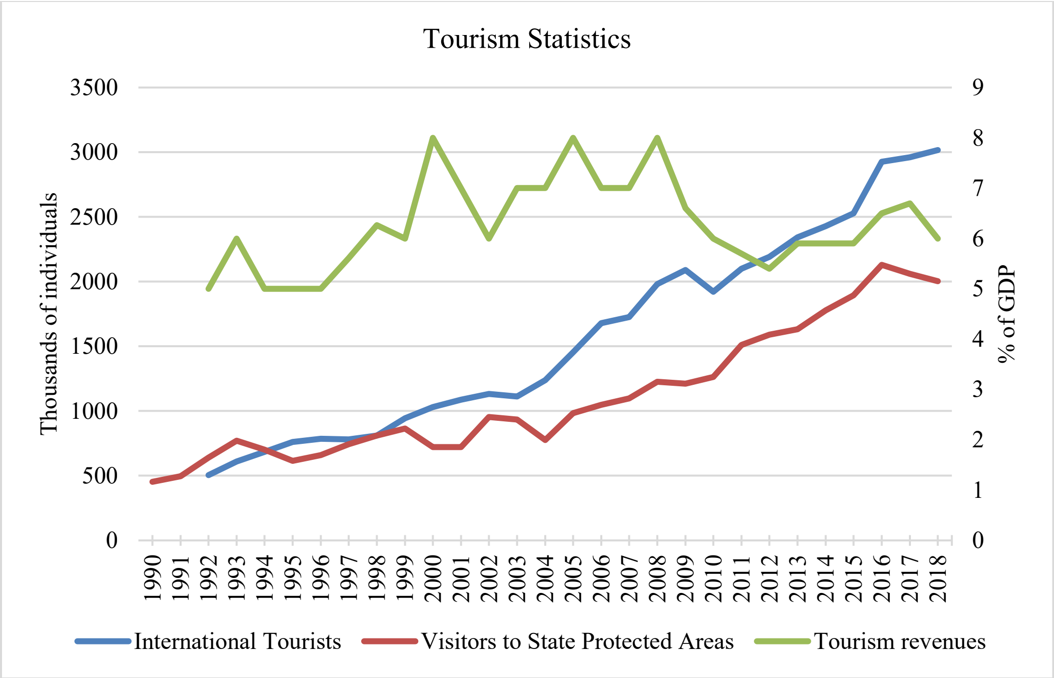

Figure CR2. Dynamics of international tourists, visitors to state protected areas and tourism revenues.

List of Holdridge's Life Zones in Costa Rica
Tropical Dry Forest
Tropical Moist Forest
Tropical Wet Forest
Tropical Premontane Moist Forest
Tropical Premontane Wet Forest
Tropical Premontane Rain Forest
Tropical Lower Montane Moist Forest
Tropical Lower Montane Wet Forest
Tropical Lower Montane Rain Forest
Tropical Montane Wet Forest
Tropical Montane Rain Forest
Tropical Subalpine Rain Paramo

References

Bernard, F., de Groot, R. S. and Campos, J. J. 2009. Valuation of tropical forest services and mechanisms to finance their conservation and sustainable use: A case study of Tapantí National Park, Costa Rica. *Forest Policy and Economics, 11*(3), 174-183.

Calvo-Obando, A. and Ortiz-Malavasi, E. 2012. Fragmentación de la cobertura forestal en Costa Rica durante los períodos 1997–2000 y 2000-2005. *Revista Forestal Mesoamericana Kurú 9*(22):10-21.

Evans, S. 2010. The green republic: A conservation history of Costa Rica. University of Texas Press.Fagan, M. E., DeFries, R. S., Sesnie, S. E., Arroyo, J. P., Walker, W., Soto, C., . . . Sanchun, A. (2013). Land cover dynamics following a deforestation ban in northern Costa Rica. *Environmental Research Letters, 8*(3), 034017. doi:10.1088/1748-9326/8/3/034017

FAO. 2010. *Global forest resources assessment 2010: main report*. Rome: Food and Agriculture Organization of the United Nations.

Ferraro, P. J. 2011. The Future of Payments for Environmental Services. *Conservation Biology, 25*(6), 1134-1138. doi:10.1111/j.1523-1739.2011.01791.x

Ferraro, P. J. and Hanauer, M. M. 2011. Protecting Ecosystems and Alleviating Poverty with Parks and Reserves: 'Win-Win' or Tradeoffs? *Environmental and Resource Economics, 48*(2), 269-286. doi:10.1007/s10640-010-9408-z

Ferraro, P. J., Hanauer, M. M., Miteva, D. A., Canavire-Bacarreza, G. J., Pattanayak, S. K. and Sims, K. R. E. 2013. More strictly protected areas are not necessarily more protective: evidence from Bolivia, Costa Rica, Indonesia, and Thailand. *Environmental Research Letters 23*(4):025011.

Ferraro, P. J., Hanauer, M. M. and Sims, K. R. E. 2011. Conditions associated with protected area success in conservation and poverty reduction. *Proceedings of the National Academy of Sciences, 108*(34), 13913-13918. doi:10.1073/pnas.1011529108

Hanauer, M. M. and Canavire-Bacarreza, G. 2015. Implications of heterogeneous impacts of protected areas on deforestation and poverty. *Philosophical Transactions of the Royal Society of London B: Biological Sciences, 370*(1681). doi:10.1098/rstb.2014.0272

Holdridge, L. R. 1967. Life zone ecology. *Life zone ecology.*(rev. ed.)).

Janzen, D. H. 1983. *Costa Rican natural history*. Chicago: University of Chicago Press.

Jones, J. 2003. *Cambios en el uso de la Tierra en Costa Rica: el mapeo y la deforestación*. San José, Costa Rica: Programa Estado de la Nación, PEN-CONARE.
Kappelle, M. 2016. *Costa Rican Ecosystems*: University of Chicago Press.
Lopez Gutierrez, B., Almeyda Zambrano, A.M., Mulder, G., Ols, C., Dirzo, R., Almeyda Zambrano, S.L., Quispe Gil, C.A., Cruz Díaz, J.C., Alvarez, D., Valdelomar Leon, V. and Villareal, E. 2019. Ecotourism: the 'human shield'for wildlife conservation in the Osa Peninsula, Costa Rica. Journal of Ecotourism, pp.1-20.
PEN-CONARE. 2018. *Estado de la Nación en desarrollo humano sostenible: un análisis amplio y objetivo sobre la Costa Rica que tenemos a partir de los indicadores más actuales 2018*. San José, Costa Rica: Programa Estado de la Nación, PEN-CONARE.
Powell, G.V., Barborak, J. and Rodriguez, M. 2000. Assessing representativeness of protected natural areas in Costa Rica for conserving biodiversity: a preliminary gap analysis. Biological Conservation, 93(1), pp.35-41.
Robalino, J. and Pfaff, A. 2013. Ecopayments and Deforestation in Costa Rica: A Nationwide Analysis of PSA's Initial Years. *Land Economics 89*(3):432-448.
Robalino, J., Sandoval, C., Barton, D. N., Chacon, A. and Pfaff, A. 2015. Evaluating Interactions of Forest Conservation Policies on Avoided Deforestation. *PLOS ONE 10*(4):e0124910. doi:10.1371/journal.pone.0124910
Sánchez-Azofeifa, A. 2015. Análisis de la cobertura forestal de Costa Rica entre 1960 y 2013. *Revista Ambientico, 253*, 4-14.
Sierra, R. and Russman, E. 2006. On the efficiency of environmental service payments: a forest conservation assessment in the Osa Peninsula, Costa Rica. Ecological economics, 59(1), pp.131-141.
Zahawi, R.A., Duran, G. and Kormann, U. 2015. Sixty-seven years of land-use change in southern Costa Rica. *PloS one*, 10(11).

The Amazon biome (12)

By Walter Cano Cardona, Wil de Jong and Benno Pokorny

Case study description

The Amazon biome, its forests, open waters and other habitats are unique for biodiversity conservation with its contiguous area of about 6.8 million km^2. It is part of nine countries with Brazil accounting for 59% of its territory, followed by Peru (11%) and Colombia (8%) (Figure AM1).

Figure AM1. Map of the Amazon biome (white line) and basin (blue line), and its constituting vegetation types. Sources: https://en.wikipedia.org/wiki/Amazon_biome and https://rainforests.mongabay.com/amazon/amazon_map.html

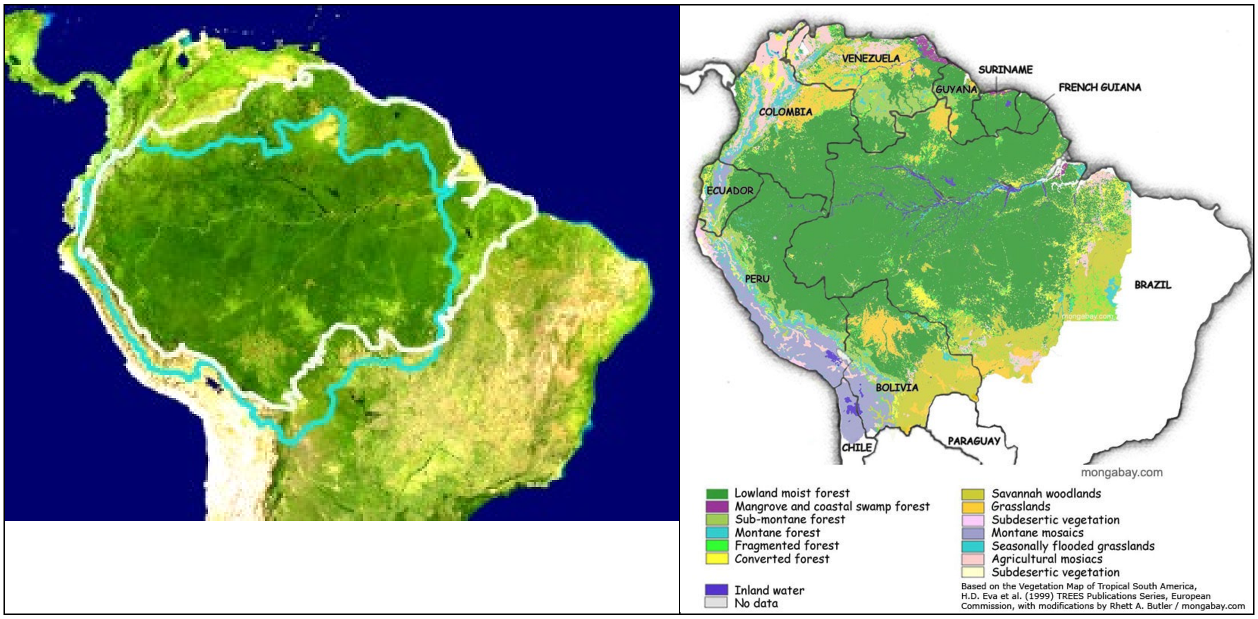

The Amazon biome's many ecosystems provide multiple services, as a terrestrial carbon repository, a regulator of near and distant weather patterns, and as a source of timber, Brazil nut, and other forest products traded in international supply chains. The region is also home to millions of people; alone in the Brazilian part of the Amazon live 30 million people[8], >76% of them in urban areas[9]. Approximately 1.7 million people belonging to 400 distinctive ethno-linguistic groups live in the Amazon, together with a large number of other rural residents. Their livelihoods highly depend on the biome's provisioning and regulatory ecosystem services. The Amazon biome is threatened by transformation to produce agricultural commodities, and expansion of roads, waterways, mineral extractions, and dams for hydropower (Walker et al. 2019). With more than 7.000 km² of annually converted forests alone in Brazil, the Amazon is one of the global hotspots of deforestation[10] (IBGE, 2020). Anticipated climate change may reduce the Amazon biome's resilience, which may possibly result in irreversible runaway degradation caused by droughts and fires (Lovejoy and Nobre, 2018).

---

[8] https://www.ibge.gov.br/apps/populacao/projecao/

[9] https://www.ibge.gov.br/estatisticas/sociais/populacao/25089-censo-1991-6.html?=&t=series-historicas

[10] https://mapbiomas.org/

Portfolios of conservation instruments and area proportions (question 1)

Amazonian countries have established several categories of protected areas that vary in accordance to objectives, functions and instruments. In general terms, there are two broader categories, integral protection and sustainable use. Within their Amazonian territories, countries have demarcated a total of 438 protected areas. The most common instruments are national parks (IUCN Category II) and protected areas with sustainable use of natural resources (IUCN Category VI) (Table AM2), the latter explicitly addressing traditional populations that receive collective rights including the possibility to continue their traditional forest uses. In many cases, also commercial timber harvesting is allowed based on a legally authorized management plan.

The magnitude of protected areas in the Amazon biome is unique worldwide, and many of the conservation instruments applied reflect the intellectual and technical up-to-date knowledge of science and practice. Brazilian protected areas alone represent a total of 1.18 million km², which corresponds to 17% of the total biome's area. Approximately 35% of these protected areas are labelled for integral protection, whereas in 65% sustainable use of the resources is legally possible.

Effectiveness of protected areas (question 2)

Studies have assessed the effectiveness of protected areas to conserve biodiversity (Maretti et al., 2014; Xavier da Silva et al., 2018; Geldmann et al., 2013), to contribute to conservation objectives (Soares-Filho, 2016), to reduce tropical forest fires (Nelson and Chomits, 2011) or deforestation (Adam et al., 2008), among others. In many countries, there are effectively working environmental institutions equipped with newest remote sensing technology. In Brazil, all conservation units together cover 28% of the national Amazonian territory, and sufficiently represent 31 out of 36 terrestrial ecoregions if considering the global average 17% Aichi Target 11 (Maretti et al., 2014). Nearly 90% of the known threatened plant species listed in the Global Strategy for Plant Conservation (GSPC) have at least one record inside at least one protected area or indigenous territory (Ribeiro et al. 2018). Also Guyana, Suriname and the French Territory have met the numerical Aichi target #11 of 17% land cover goal of protected areas (22%) at the regional level. The rest of the countries are making significant efforts to reach at least the Aichi protected area target. Progress towards more effective implementation of national protected area policies and programs is a common story in all countries of the Amazon basin, because of national commitments, increasing environmental funding, frequently with international support, more effective technologies, and better trained staff.

However, serious limitations of the effectiveness of conservation efforts include illegal activities by unscrupulous entrepreneurs, but sometimes also small producers who are eking out a meager livelihood and engage in, for instance, illegal opening of forest to grow food (Maretti et al. 2014). While Nepstad et al. (2014) point at high conservation effects of indigenous territories, more recent studies suggest that also populated conservation units lack the capacity to resist the continuously increasing pressure from economic actors interested in land and resources (Benyishay et al., 2017). Even lower is the conservation effect of the forest areas harvested for timber even in the case of a legally authorized management plan and FSC certification. Most of these areas, once logged, are further degraded and subsequently converted into pastures, except for only a handful of management units under the control of international companies (Rana and Sills 2018). A continuously growing road network, be it constructed by the state or, informally by timber companies, cattle ranchers, agro-industrial investors or speculators, provides access for

land users into the most remote locations. In the case of established accessibility, prospected oil, gas and mineral deposits, and the possibility for the construction of hydro power plants, eventually existing protection status of a forest is easily ignored. Legal and informal settlements play a role, however, to a lesser degree. More than 80% of newly deforested land within Brazilian's legal Amazon is initially used for cattle.

Table AM2. Relative amounts of protected area among countries in the Amazon biome (white line in Figure AM1) using IUCN's categories (Prüssmann et al., 2017).

**Country (National Amazon territory in 1000 km²)**	**Proportion of IUCN categories in Amazon biome (%)**								**Total**	
	**Ia**	**Ib**	**II**	**III**	**IV**	**V**	**VI**	**Not reported**	**Protected (%)**	**Not protected[11] (%)**
Brazil (4.050)	13.7		21.32		0.02	13.26	45.38	6.33	27.19	72.81
Venezuela (460)			28.62	6.98		15.66	48.54	0.21	57.58	42.42
Bolivia (410)		9.94	39.78		10.17		9.35	30.75	26.53	73.47
French Guiana (90)		2.54			6.76	44.22		46.48	52.09	47.91
Peru (770)			44.33	1.73			34.15	19.79	21.01	78.99
Colombia (540)	20.8		78.25					0.12	19.07	80.93
Ecuador (120)			64.48				35.41		24.80	75.20
Guiana (240)		3.47					96.53		8.32	91.68
Suriname (160)			45.94		9.73		7.28	37.06	14.26	85.74
**Total area (1000 km²)**	**175.8**	**1.3**	**550.9**	**25.0**	**14.1**	**188.4**	**767.0**	**134.5**	**1857.3**	**4982.7**

Progress towards more forest conservation areas is constrained by two main factors: lacks of political will in support of protected areas and social support for protected areas. The large extent of the Amazon territory in combination with budget limitations of the countries concerned result in a quasi-absence of the state on the ground. Persistent challenges for protected areas continue to be illegal mining operations, hunting, illegal logging, and, most important, conversion for agriculture, including overall cattle but also the illicit production of coca leaves. Lack of political continuity and commitment also plays a role. In Brazil, for example, the Bolsonaro administration in power since 2019 has in a very short time reversed important achievements in the protection of natural forests and Indigenous Peoples, which were worked out over a long period of time (Viola and Gonçalvez, 2019). Challenges are quite similar, for instance in Brazil, Bolivia, Colombia, Ecuador and Peru.

Policy implementation tools (question 3)

"Carrot": Sustainable forest management by professional timber companies continues in concessions inside some of the protected areas as well as other public forests.

"Stick": A number of strategies and tools have been defined to respond to fight against degradation of protected areas. Protected area demarcation continues, but has strongly slowed down. In Brazil, for example, since 2009, no new indigenous territory has been established (Maretti et al. 2014). In some countries such as Brazil, there is a parallel tendency to re-centralize key competences for the application of standardized sets of methodological procedures that allow for automatized monitoring and sanctioning based on remote sensing data. Efforts to

[11] The column shows the proportion of Amazon ecosystem under any category of protection of each country, compared with the area not protected

clarify the land status continues, like in Brazil, which has been investing in a rural environmental cadaster (Cadastro Ambiental Rural - CAR[12]). There appears, however, to be a threat that powerful actors take advantage of CAR to occupy land at the expense of small farmers, and traditional and indigenous communities (Jung et al. 2017).

"Sermon": Governmental agencies still technically support indigenous and traditional communities in and outside of protected areas to commercially use their forest. The idea to enhance local participation of protected area residents in the management of their resources to create interest in contributing to the protected area goals. However, in practice this has dramatically lost importance because of large transaction and opportunity costs for both sides, and a lack of willingness of governments to adapt legislation to local realities (Pokorny, 2013). There is ongoing investment in strengthening the institutional enforcement capacity of environmental agencies to monitor and control management plans and stop illegal deforestation. Another strategic effort to increase protected area effectiveness is regional integration. The "Integration of Protected Areas from Amazon Biome (IAPA)", for instance, is a joint effort between FAO, WWF, UICN and UNEP, that seeks to build a network of protected areas of the Amazon region. IAPA has supported the development of a protocol to measure management effectiveness. The main criteria applied to design the protocol pointed out to "why protected areas are effective" instead of the traditional simple understanding of "whether protected areas are effective or not". The protocol uses five main indicators: governance, climate change impacts, management strategies, socio-environmental impacts, and compliance with conservation objectives. The recurrently adapted protocol is expected to improve the integration of national decision-making in all Amazon countries to achieve a shared region-wide vision. First results of its application in 62 protected areas of Bolivia, Brazil, Colombia, Ecuador and Peru suggest that compliance with conservation objectives has improved, but that protected area management continues to be a major challenge.

Net effect of conservation and intensification (question 4)

Sustainable resource use in protected areas remains a challenge because maximum yields recommended in regulations are usually higher than those suggested by scientific studies, for instance in the case of timber extraction. It is not likely that this will change, even though data from permanent sample plots and improved and remote sensing are improving. The existing regulations and bureaucracies for the commercial harvest of natural resources (timber, NTFPs, fish) has created an overload of regulations for local resource users that they do not have the technical and financial capacities to comply with, and often force people to engage in unauthorized use and harvesting. To avoid dependency on NGOs or, even more common, commercial entrepreneurs, it is necessary to recognize local interests, knowledge, and practices as the basis for sustainable use of protected area's natural resources.

While an Amazon biome wide integration of protected areas is progressing, major challenges remain. Co-management and co-governance of protected areas is indispensable but still a distant goal because governments and their agencies continue to dominate both. The management of transboundary protected areas suffers from incompatible legal frameworks among countries and federative states in Brazil that need a profound revision to achieve common goals. Additionally, it is unclear how to continuously guarantee the funds needed by governments to properly manage

[12] http://www.car.gov.br/#/

protected areas, and what can be done to satisfy and effectively control the myriads of economic actors interested in land and resources to be protected. Biodiversity conservation is combined with declared sustainable development goals that actually include generating benefits to local residents, but also income to meet the economic needs of protected areas to reduce the economic burden on the state. This results in management that endangers biodiversity conservation goals and local livelihoods.

References

Andam, K.S., Ferraro, P.J., Pfaff, A., Sanchez-Azofeifa, G.A and Robalino, J.A. 2008. Measuring the effectiveness of protected area networks in reducing deforestation. *Proceedings of the National Academy of Sciences* 105(42):16089–16094.

Arguedas, S.; Vides, R. and Castaño, L. (Eds). 2015. Lecciones aprendidas y buenas prácticas para la gestión de áreas protegidas amazónicas. UICN-Fundación Gordon y Betty Moore. Quito, Ecuador. 264 pp.

Benyishay, A., Heuser, S., Runfola, D. and Trichler, R. 2017. Indigenous land rights and deforestation: Evidence from the Brazilian Amazon. *Journal of Environmental Economics and Management* 86:29-47. doi:10.1016/j.jeem.2017.07.008

FAO. 2016. Latin American Experiences in Natural Forest Management Concessions, by, Steve Gretzinger. Forestry Policy and Institutions Working Paper No. 35. Rome.

Fearnside, P.M. 2005. Deforestation in Brazilian Amazonia: History, rates and consequences. Conservation Biology 19(3):680-688. DOI:10.1111/j.1523-1739.2005.00697.x

Geldmann, J., Barnes, M., Coad, L., Craigie, I., Hockings, M. and Burgess, N. 2013. Effectiveness of terrestrial protected areas in reducing biodiversity and habitat loss. CEE 10-007. Collaboration for Environmental Evidence: www.environmentalevidence.org/ SR10007.html.

Hockings, M., Leverington, F. and James, R. 2003. Evaluando la efectividad de manejo: La conservación de las áreas protegidas ahora y en el futuro. Background report prepared for the V World Parks Congress, Durban. IUCN World Commission of Protected Areas.

Jung, S., Rasmussen, L.V., Watkins, C., Newton, P. and Agrawal, A. 2017. Brazil's national environmental registry of rural properties: Implications for livelihoods. *Ecological Economics* 13653–61.

Lovejoy, T.E. and Nobre, C. 2018. Amazon tipping point. Sci. Adv., 4: 2340

Martins, E., Loyola, R. and Martinell, G.2017. Challenges and perspectives for achieving the global strategy for plant conservation targets in Brazil. Annals Missouri Botanical Garden 102: 347–356.

Maretti, C.C., Riveros, S.J.C., Hofstede, R., Oliveira, D., Charity, S., Granizo, T., Alvarez, C., Valdujo, P. and Thompson C. 2014. State of the Amazon: Ecological Representation in Protected Areas and Indigenous Territories. Brasília and Quito: WWF Living Amazon (Global) Initiative. 82pp.

Metzger, J.P., et al. 2019. Why Brazil needs its Legal Reserves. *Perspectives in Ecology and Conservation* 17:91–103.

Nelson, A.and Chomitz, K.M. 2011. Effectiveness of Strict vs. Multiple Use Protected Areas in Reducing Tropical Forest Fires: A Global Analysis Using Matching Methods. PLoS ONE 6(8): e22722. doi:10.1371/journal.pone.0022722

Nepstad, D., McGrath, D., Stickler, C., Alencar, A., Azevedo, A., Swette, B., Bezerra, T., DiGiano, M., Shimada, J., Seroa da Motta, R., Armijo, E., Castello, L., Brando, P., Hansen,

M.C., McGrath-Horn, M., Carvalho, O. and Hess, L. 2014. Slowing Amazon deforestation through public policy and interventions in beef and soy supply chains. *Science*: 1118-1123
Navarrete, S. 2018. Protocolo y guía metodológica para medición de efectividad del manejo del bioma amazónico. Proyecto IAPA – Visión Amazónica. Unión Europea, REDPARQUES, WWF, FAO, UICN, ONU Medio Ambiente. Bogotá, Colombia.
Pokorny, B. 2013. Smallholders, forest management and rural development in the Amazon. Earthscan Forest Library/Routledge, Oxon. 212p
Prüssmann J., C. Suárez and M. Chaves. 2017. Atlas de oportunidades de conservación en el bioma amazónico bajo consideraciones de cambio climático. Iniciativa Visión Amazónica. REDPARQUES, WWF, FAO, UICN, PNUMA. Cali, Colombia. 80 p.
Rana, P. and Sills, E.O. 2018. Does Certification Change the Trajectory of Tree Cover in Working Forests in The Tropics? An Application of the Synthetic Control Method of Impact Evaluation. Forests 9, 98.
REDPARQUES. 2010. Informe: Avances en el desarrollo del Programa de Trabajo Sobre Áreas Protegidas - Región: Bioma Amazónico. Conservación de la Biodiversidad Biológica – UICN – WWF.
REDPARQUES. 2016. Regional report implementation of the program of work on protected areas 2011 - 2015: Amazonian biome region. Bogota, Colombia. 115 p.
REDPARQUES 2018. Avances en la implementación del Programa de Trabajo sobre Áreas Protegidas 2016-2017: Región Bioma Amazónico. Proyecto IAPA – Visión Amazónica. REDPARQUES, WWF, FAO, UICN, ONU Medio Ambiente. Bogotá, Colombia. 36p.
Ribeiro R. B., Martins, E., Martinelli, G. and Loyola, R. 2018. The effectiveness of protected areas and indigenous lands in representing threatened plant species in Brazil. Rodriguésia 69(4): 1539-1546. DOI: 10.1590/2175-7860201869404
Soares-Filho, B.S. 2016. Role of Amazon protected areas, especially the conservation units supported by ARPA, in reducing deforestation. Rio de Janeiro: Funbio, 2016.
Viola, E. and Korber Gonçalves, V. 2019. Brazil ups and downs in global environmental governance in the 21st century. Revista Brasileira de Política Internacional 62(2): e010
Visión Amazónica – REDPARQUES. 2017. Dossier: Informe de Avances del Proyecto Integración de las Áreas Protegidas del Bioma Amazónico (IAPA) 2015 – 2017.
Visión Amazónica – REDPARQUES. 2019. Dossier: Informe final de resultados del Proyecto Integración de las Áreas Protegidas del Bioma Amazónico (IAPA), 2015 – 2019.
Walker, T.R, Simmons, C., Arima, E., Galvan-Miyoshi, Y., Antunes, A., Waylen, M., Irigaray, M. 2019. Avoiding Amazonian Catastrophes: Prospects for Conservation in the 21st Century. *One Earth* 1 (2) 202-215.
WWF (Iniciativa Amazonía). 2014. Áreas protegidas y territorios indígenas.
Xavier da Silva, M., Paviolo, A., Tambosi, L.R. and Pardini, R. 2018. Effectiveness of Protected Areas for biodiversity conservation: Mammal occupancy patterns in the Iguaçu National Park, Brazil. J. *Nat. Conserv*. 41:51–62.

Argentina (13)

By Guillermo Martínez Pastur and Yamina Micaela Rosas

Case study description

Argentina present greatly changes in topography across more than 5000 km (north to south) which generated large variability in climate. These conditions allowed to present different forest types, from rainforests to temperate cold forests. For this, usually Argentina is divided at least in six forest regions. The forest cover of Argentina reaching to 54.19 mill ha, which include near 1 mill ha of plantations and other agricultural forests (e.g. windbreaks), which represent the 19.41% of the total territory.

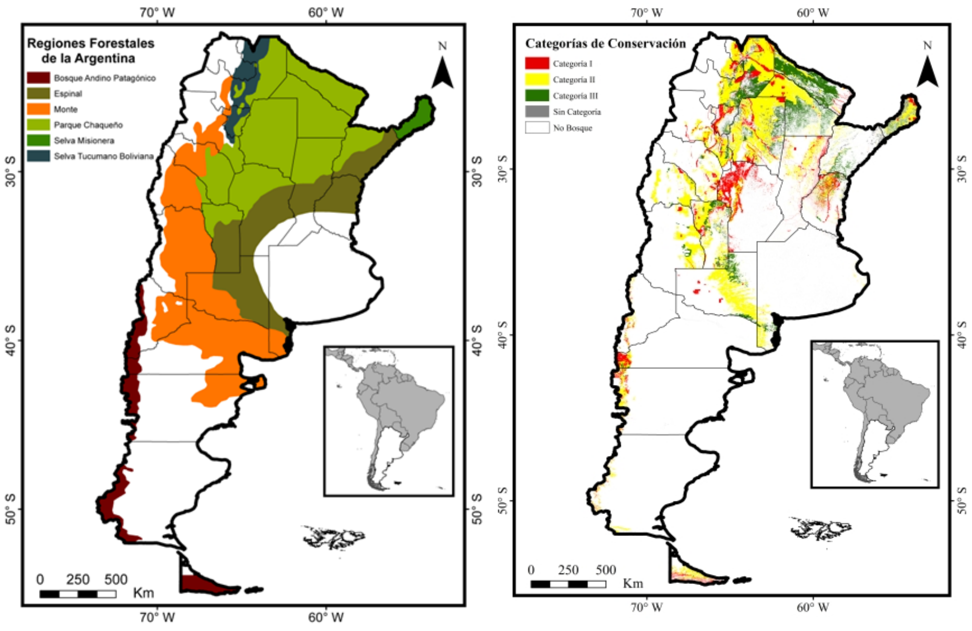

Figure ARG1. Argentina forest regions (UMSEF 2014), and National Ordination for Native Forest (Law 26.331) including the three categories (red = strict protection, yellow = sustainable management, green = allowed to land use change or not) (Martínez Pastur et al. 2020).

Portfolios of conservation instruments and area proportions (question 1)

In Argentina set-aside forests can be divided in three types. One is represented by the formal protection network (national parks, provincial or municipality reserves, and private initiatives). The formal protection reaches to 5.48% of the total forest cover in Argentina, but was unequally represented in the different forest regions (1.67% to 22.94%). The other categories are defined by the national law 26.331 and provincial forest regulations. This law categorize all the forests in Argentina according to three colours (article 9): (i) red (high conservation value forests for ancestral uses, gathering of non-timber forest products, scientific research, conservation plans, ecological restoration); (ii) yellow (medium conservation value forests for sustainable productive activities and tourism under the guidelines of management and conservation plans); and (iii) green (low conservation value forest where land-use change is allowed). The red forests can be considered as voluntary set-aside or unproductive lands due to any economic activity can be conducted in these woods. This protection category reaches to 17.33% of the total forest cover in Argentina, but was unequally represented in the different forest regions (0.43% to 27.34%). In

the yellow areas the forests can be used, harvested or managed according sustainable practices that greatly varied across the territory and forest types, e.g. shelterwood cuts or variable retention cuts in the temperate forests of the South, to selective cuts in the rainforests or silvopastoral systems in the Chaco forests. Beside this, all the proposals lead to maintain some retention percentage (15% to 50%), where clear-cuts are no longer possible in most of the natural forests areas. The percentage of retention in the yellow areas can be considered as the tree retention category of this work. The retention protection reaches to 13.09% of the total forest cover in Argentina, but was unequally represented in the different forest regions due to the different silvicultural practices (8.16% to 30.14%). As a whole, the different set aside forests in Argentina reached to 35.90% of the total forest area.

COUNTRY	Argentina has 279.13 mill ha, and has 54.19 mill ha of forest cover (natural forests, plantations and other types).					A Sum
REGION	Forest Area	Formal protection (%)	Voluntary set-aside (%)	Tree retention (%)	Unproductive (%)	(%)
**Argentina**	**54.19 mill ha**	**5.48**	**17.33**	**13.09**		**35.90**
AR-Patagonian Forests	3.37 mill ha	22.94	27.34	18.9		69.18
AR-Espinal Region	7.99 mill ha	1.67	16.47	14.68		32.82
AR-Monte Region	4.82 mill ha	6.1	8.77	19.59		34.46
AR-Chaco Park	30.69 mill ha	3.57	17.88	8.16		29.61
AR-Misiones Rainforests	1.58 mill ha	15.22	0.43	24.24		39.89
AR-Yunga Rainforests	4.82 mill ha	7.86	20.56	30.14		58.56
AR-None	0.92 mill ha	5.88	26.82	0		32.70

Formal protection includes national parks, provincial reserves and private reserves
Voluntary set-aside represent the red forests according to national law 26.331 that push to forest owner to maintain forests with uses for conservation.
Tree retention is based on the different silviculture practices of forest regions, and the % of basal area that must be maintained, and that can be possible to apply in yellow forests (note: maybe less than 50% of forests actually is under forest management, however, they are intensively used for other purposes).
Unproductive, i.e. most of the forests outside reserves are used for different purposes, lumber, cattle, forestry

Effectiveness (question 2)

The Argentinian National Law 26,331/07 constitutes a legal norm in which the use of natural resources for productive purposes was prioritised without limitations. This generated different reactions in the society, and the national and provincial authorities had to adjust their structure and operations to implement the law. The implementation of the law delayed or paralyzed processes of approving forest plans. While this process did not stop of deforestation processes (Figure ARG2), it led to a general decrease in the forest loss rate. Payments made for conservation of native forests thus became a useful tool to reduce the conversion rates, but this tool did not stop the deforestation process completely. On the other hand, forest loss may be due to natural factors (e.g., landslides, windthrows) or derived from human actions, both indirect (e.g., fires) and directly related to productive activities. These factors varied among the provinces, mainly associated with population density, agricultural activity and livestock, which is also related to the fires and harvesting. In the particular case of soybean crops, they can explain most of the deforestation in many provinces.

Policy implementation tools (question 3)

"Carrot": Payment of the ecosystem services can be used to improve the well-being of the society (Engel et al. 2008; Zheng et al. 2013). In this context, the national law 26,331/07 was promulgated in Argentina, and was named as "Minimum Budgets for Environmental Protection of the Native Forests". This law involves many challenges (MAyDS, 2017): (i) changes in forest management and forest cover proposed by the owners must be complemented by a great social awareness (e.g. protection of natural environments classified as high conservation value); (ii) modifications in the original forest cover must be accompanied by the proposal of new practices that must be in accordance with the law (e.g., silvopastoral systems instead of forest removal and pasture implantation); (iii) changes in the forest cover imply several administrative restrictions that will only see the benefits in the medium term; and (iv) the policies must be designed for the long-term, so the proposals must be solid and resilient to the socio-economic changes over the years. Three categories were defined in this national law (article 9) (Martínez Pastur et al. 2020): (i) Category I (red) are areas of high conservation value that must not be modified and conserved untouched. This category must include areas for landscape connectivity, biological values and protection of watersheds that merit their persistence as a forest in perpetuity, although these sectors can be habitat for indigenous communities and be the subject of scientific research. (ii) Category II (yellow) are areas of medium conservation value, which may be degraded in the past, but which in the judgment of the jurisdictional authority can be restored due to their high conservation value, and may be subject to the following uses: sustainable use, tourism or scientific research. (iii) Category III (green) are areas with low conservation value that can be partial or completely transformed.

"Stick": Regulatory mechanisms to promote the provision of ecosystem services of regulation and the biodiversity conservation (Saarikoski et al. 2018).

"Sermon": These synergies and trade-offs of land use generate higher controversies in the society, which demands a well-being based on productive activities (e.g., provision ecosystem services), but also claim for the benefits of other ecosystem services (e.g., regulatory, support or cultural ecosystem services) and the biodiversity conservation (Martínez Pastur et al. 2017, 2018; Turkelboom et al. 2018). To find a solution for these trade-offs, the governments established a dialogue among the different actors of the society.

Net effect of conservation and intensification (question 4)

Argentina present a great diversity of climates and environments, that favoured the establishment of different productive initiatives, mainly related to agricultural and forestry activities based on exotic species (e.g. cattle, extensive crops and forest plantations). When these productive initiatives are based on goods and services provided by the natural ecosystems, positive synergies can be achieved, that can lead to the effective conservation of these natural environments, and leading in the searching of sustainable management proposals (Martínez Pastur et al. 2020). However, most of these productive proposals are opposed to the persistence of the natural ecosystems, generating trade-offs that leading to the changes in land use over time (Luque et al. 2010). These changes generate alterations in the structure and assemblage of the typical species of the natural ecosystems, which culminates in the local extinction, generating an artificialization of the natural landscapes (e.g. monocultures for agriculture or forestry production). Recently, new alternatives have been proposed that combine economic and conservation purposes in the same area (e.g. Lindenmayer et al. 2012). However, this new management perspective has not declined the artificialization of the natural ecosystems (Martínez Pastur et al. 2020).

The first approach of conservation both in biodiversity values and in the provision of ecosystem services was based on the preservation of wild or natural environments within strict reserves. This strategy creates a division in the management and planning of the landscape: (i) within the reserves where the inalterable nature of the ecosystem was promoted, and (ii) outside reserves where all transformation to maximize provisioning services are feasible to implemented (Swallow et al. 2009). However, this strategy was ineffective to conserve the provision of non-monetary ecosystem services and all the biodiversity at a regional level, and fails in protect the species for which many of these reserves were created (Lindenmayer et al. 2012). Argentina, like many other countries, has based its conservation strategy on the creation of National Parks located in remote or border areas (Hopkins 1995; Izquierdo and Grau 2009; Swallow et al., 2009), while the rest of the landscape was and is under a continuous processes of deforestation and land use changes, which increased over time since the beginning of European colonization. These processes generate a significant regression of the natural ecosystems, as well as for the communities of original inhabitants that used them to live on (Boletta et al. 2006; Gasparri and Grau 2009; Cáceres 2015). In this international context, the national law 26,331 (November 2007) was enacted to regulate the protection, enrichment, restoration, use and management of native forests and the environmental services that they produce (Seghezzo et al. 2011). This law finances actions, which were designed as a strengthening of the institutions and an extra income for the forest producers (Martínez Pastur et al. 2020). This program was financed by the national government for the owners of the native forest (provincial governments, institutions and private sector) to ensure and maintain the provision of ecosystem services over time. This payment includes: (i) to limit the land use change in the native forestlands, (ii) to conduct sustainable management practices, and (iii) to increase biodiversity preservation areas within the matrix of the productive landscape. Few initiatives of this kind exists in the World, and it is of interest to understand how these investments have been implemented within the framework of this national law, and to analyze the impact of this initiative in accordance with the proposed objectives. It is necessary to analyze the payments implemented by the national government to the beneficiaries according to their jurisdiction, considering the previous sections related to forest cover losses (before, during and after the enactment of national and provincial laws).

The national law 26,331/07 constitutes an unusual legal norm for a country in which the use of natural resources for productive purposes was priorized without limitations along their history. This generated different reactions in the society, and the national and provincial authorities had to adjust their structure and operations to successfully implement the law along the country. It is not possible to relate the application of the law to the stop of the deforestation processes; however, we can relate this process to a decrease in the forest loss rate. Still, we don't know if in the future the price in some crops can pressure again over the forests with the consequent deforestation. The law, through the OTBN, achieved in the practice the effective ordination of new agricultural production initiatives, limiting their operations to those areas classified as green. Beside this, an intense debate was installed in the society, as a result of which many sectors were able to increase their knowledge about native forests and their importance as producers of ecosystem goods. However, the implementation of the law was not perfect. It is very important to detect the points in which the process of approval of plans was delayed or paralyzed, and it is necessary to remove those bottlenecks and achieve times compatible with the expectations of the producers (Martínez Pastur et al. 2020).

In few words, the natural environmental frontier in Argentina is moving backward, we continue loos natural forest cover every year, and both provincial and national initiatives are still not enough to stop these changes. We must consider that the country main incomes are based on commodities (e.g. soybean).

Silvicultural systems allowed maintaining the natural forests inside the productive matrix (e.g., silvopastoral systems) can generate an equilibrium between some of the most important rural activities (e.g. livestock) and conservation (e.g. 30-50% retention). In some specific forest regions (e.g. Patagonian Forests) the timber are the main economic activity in some natural forest types, and in these specific forests, the silviculture play an important role, both to support the provision of ecosystem services and for biodiversity conservation (e.g. variable retention approach). Many silvicultural proposals are based on natural forest disturbance regimes (e.g. gap or windthrow), which are the basis to the development of the different proposals (e.g. shelterwood cuts, group selection cuts or aggregate retention). However, some other silvicultural proposals are deliberate to modify the naturalness of the stands, eliminating some undesirable components (this is the most common situation in Argentina), e.g. silvopastoral systems in Chaco Park remove the shrub component, leaving the large trees and planting new palatable species in the understory layer. We can conclude that this point is quite relevant for the incoming years discussion in Argentina, and due to it quiet variable among regions, it is necessary specific considerations among the different forest types and the management objectives of the different areas (conservation, timber, cattle, tourism, etc.).

Have policy instruments been effective to stop deforestation in Argentina? At country level, there is a continuous loss of forest cover for the analysed period, from 42.4 to 37.7 million hectares. In the analysis of the balance of gains and losses in forest cover, these magnitudes and rates are dissimilar. The rates of gains varied between 240 and 450 $km^2 year^{-1}$, while losses varied from 1000 to 5750 km^2 annually. The gains are associated with recoveries of the native forest in their natural dynamics after natural (e.g., windthrows) or anthropogenic disturbances (e.g., growth of regeneration after harvesting), or even indirect human effects (e.g., fires). In addition,

these gains can be due to exotic forest plantations for commercial or other purposes (e.g. windbreaks plantations to protect crops).

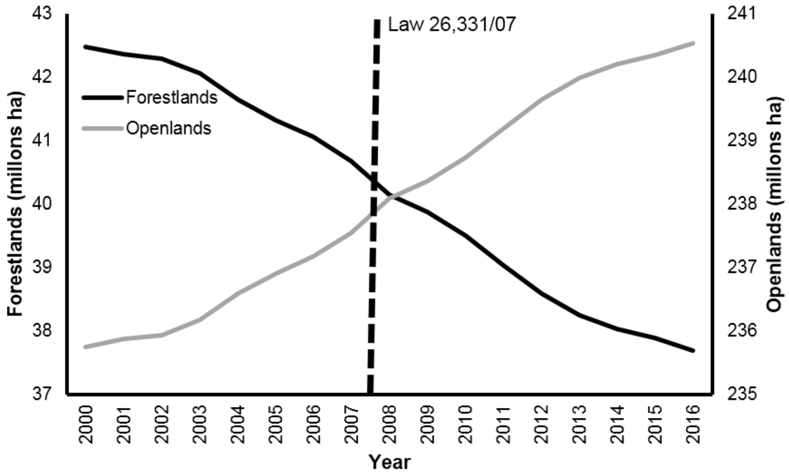

Figure ARG2. Changes in forest cover and openlands of Argentina (period 2000-2016), before and after the implementation of the lay 26,331/07 (Martínez Pastur et al., 2020).

References

Boletta, P., Ravelo, A., Planchuelo, A. and Grilli, M. 2006. Assessing deforestation in the Argentine Chaco. Forest Ecology and Management 228, 108-114.

Cáceres, D. 2015. Accumulation by dispossession and socio-environmental conflicts caused by the expansion of agribusiness in Argentina. Journal of Agrarian Change 15(1), 116-147.

Engel, S., Pagiola, S. and Wunder, S. 2008. Designing payments for environmental services in theory and practice: An overview of the issues. Ecol. Econ. 65(4), 663-674.

Gasparri, N.I. and Grau, R. 2009. Deforestation and fragmentation of Chaco dry forest in NW Argentina (1972-2007). Forest Ecology and Management 258, 913-921.

Hopkins, J.W. 1995. Policymaking for conservation in Latin America: National Parks, reserves, and the environment. Ed. Praeger, Westport, US. 216 pp.

Izquierdo, A.E., De Angelo, C. and Aide, M. 2008. Thirty years of human demography and land-use change in the Atlantic forest of Misiones, Argentina: An evaluation of the forest transition model. Ecology and Society 13(2):3.

Lindenmayer, D., Franklin, J., Lõhmus, A., Baker, S., Bauhus, J., Beese, W., Brodie, A., Kiehl, B., Kouki, J., Martínez Pastur, G., Messier, Ch., Neyland, M., Palik, B., Sverdrup-Thygeson, A., Volney, J., Wayne, A. and Gustafsson, L. 2012. A major shift to the retention approach for forestry can help resolve some global forest sustainability issues. Conservation Letters 5(6), 421-431.

Luque, S., Martínez Pastur, G., Echeverría, C. and Pacha, M.J. 2010. Overview of biodiversity loss in South America: A landscape perspective for sustainable forest management and conservation in temperate forests. In: Landscape Ecology and Forest Management: Challenges and Solutions in a Changing Globe (Li, C., Lafortezza, R., Chen, J., Eds.). HEP-Springer. Amsterdam, Holanda. Cap. 15, pp 352-379.

Martínez Pastur, G., Peri, P.L., Huertas Herrera, A., Schindler, S., Díaz Delgado, R., Lencinas M.V. and Soler, R. 2017. Linking potential biodiversity and three ecosystem services in

silvopastoral managed forests landscapes of Tierra del Fuego, Argentina. International Journal of Biodiversity Science, Ecosystem Services and Management 13(2), 1-11.

Martínez Pastur, G., Perera, A., Peterson, U. and Iverson, L. 2018. Ecosystem services from forest landscapes: An overview. In: Ecosystem services from forest landscapes: Broadscale considerations (Perera, A., Peterson, U., Martínez Pastur, G., Iverson, L., Eds.). Pp 1-10. Ed. Springer, Cham, Switzerland.

Martínez Pastur, G., Schlichter, T., Matteucci, SD., Gowda, J., Huertas Herrera, A., Toro Manríquez, M., Lencinas, M.V., Cellini, J.M. and Peri, P.L. 2020. Synergies and trade-offs of national conservation policy and agro-forestry management over forest loss in Argentina during the last decade. In: Latin America in times of global environmental change (C Lorenzo, Ed.). Pp 135-155. The Latin American studies book series. Ed. Springer, Cham, Switzerland (ISBN 978-3-030-24253-4).

Ministerio de Ambiente y Desarrollo Sustentable (MAyDS), 2017. Informe de estado de implementación 2010-2016 de la Ley N°26.331 de Presupuestos Mínimos de Protección Ambiental de los Bosques Nativos. MAyDS, Buenos Aires, Argentina. 41 pp.

Saarikoski, H., Schleyer, Ch., Primmer, E., Aszalós, R., Baró, F., Berry, P., Garcia Blanco, G., Goméz-Baggethun, E., Carvalho, L., Dick, J., Dunford, R., Hanzu, M., Izakovicova, Z., Kertész, M., Kopperoinen, L., Köhler, B., Langemeyer, J., Liquete, C., Luque, S., Mederly, P., Niemela, J., Palomo, I., Martínez Pastur, G., Peri, P.L., Preda, E., Priess, J., Saarela, S.R., Turkelboom, F., Vadineanu, A., Verheyden, W., Vikström, S. and Young, J. 2018. Institutional challenges in putting ecosystem services in practice. Ecosystem Services 29:579-598.

Seghezzo, L., Volante, J., Paruelo, J., Somma, D., Buliubasich, C., Rodríguez, H., Gagnon, S. and Hufty, M. 2011. Native forests and agriculture in Salta (Argentina): Conflicting visions of development. Journal of Environment and Development 20(3), 251-277.

Swallow, B., Kallesoe, M., Iftikhar, U., van Noordwijk, M., Bracer, C., Scherr, S., Raju, K., Poats, S., Kumar Duraiappah, A., Ochieng, B., Mallee, H. and Rumley, R. 2009. Compensation and rewards for environmental services in the developing world: Framing pan-tropical analysis and comparison. Ecology and Society 14(2), e26.

Turkelboom, F., Jacobs, S., Leone, M., Kelemen, E., García Llorente, M., Baró, F., Berry, P., Termansen, M., Barton, D., Stange, E., Thoonen, M., Kalóczkai, A., Vadineanu, A., Castro, A., Czúcz, B., Röckmann, C., Wurbs, D., Odee, D., Preda, E., Gómez-Baggethun, E., Rusch, G., Martínez Pastur, G., Palomo, I., Dick, J., Casaer, J., Van Dijk, J., Priess, J., Langemeyer, J., Mustajoki, J., Kopperoinen, L., Baptist, M., Peri, P.L., Mukhopadhyay, R., Aszalós, R., Roy, S., Luque, S., Rusch, V., 2018. When we cannot have it all: Ecosystem services trade-offs in real-life planning contexts. Ecosystem Services 29, 566-578.

Unidad de Manejo del Sistema de Evaluación Forestal (UMSEF), 2014. Ministerio de Ambiente y Desarrollo Sustentable de la Nación (MAyDS). Regiones Forestales de Argentina. Mapas Ambientales (http://mapas.ambiente.gob.ar/).

Zheng, H., Robinson, B., Liang, Y., Polasky, S., Ma, D., Wang, F., Ruckelshaus, M., Ouyang, Z. and Daily, G., 2013. Benefits, costs, and livelihood implications of a regional payment for ecosystem service program. PNAS 110(41), 16681-16686.

Madagascar (14)

By Lucienne Wilmé, Ollier D. F. Andrianambinina, Serge C. Rafanoharana and Patrick O. Waeber

Case study description

Madagascar is a large geologically ancient island, separated from any other land since the Cretaceous, and mostly pantropical. Its biodiversity is unique and most groups of plants and animals exhibit endemism rates ranging from at least 50% to almost 100%, with high level of micro-endemism (Wilmé et al. 2006, 2012). Most of this biodiversity occurs in forests (80%) while the forests cover barely 15% of the island, and continue to decline every year (Waeber et al. 2020). The main threats to Madagascar's forests are deforestation and the permanent transformation of the habitat through slash and burn cultivation (Styger et al. 2007).

Portfolios of conservation instruments and area proportions (question 1)

A majority of the >14 000 plant species are encountered in forests, which encompass mangroves, humid and sub-humid forests, dry forests and dry spiny forests and thickets as well as coastal bushland (Figure MA1). Since the onset of the 20th century, naturalists have alerted on the consequences of the deforestation for biodiversity conservation, and the system of protected areas has expanded (Figure MA1).

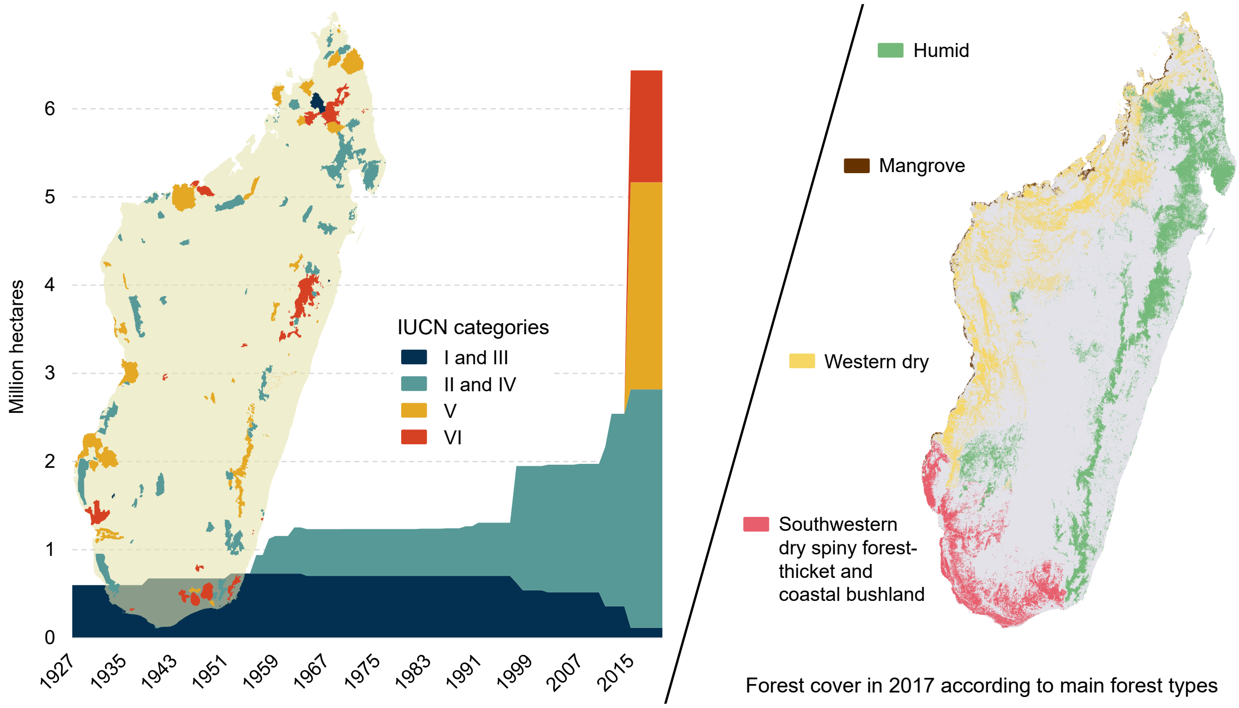

Figure MA1. Distribution of the main types of forests (left) and evolution of the conservation status of the system of protected areas in Madagascar (right). Forest types according to Moat & Smith (2007); forest cover according to Vieilledent et al. (2018); modified from Waeber et al. (2020).

The area of the parks and reserves have increased over the last decades with protected areas of various conservation status, with few excluding people—IUCN category I—and a large increase in people inclusive protected area—IUCN categories V and VI (Gardner et al. 2018; Figure MA2).

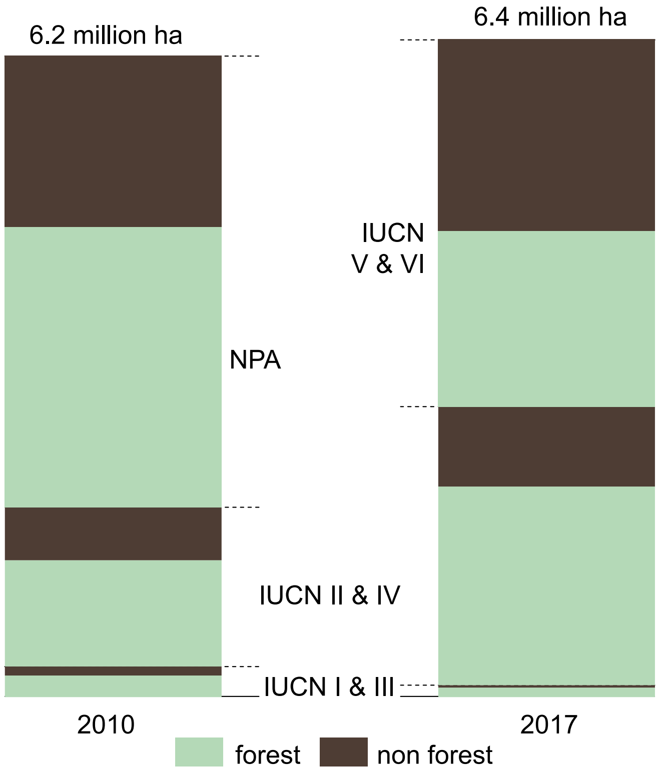

Figure MA2. Evolution of the IUCN conservation status of the system of protected areas in Madagascar from 2010 to 2017, and representation of forest within the categories. (NPA = new protected area; I to VI = IUCN conservation categories.

Effectiveness (question 2)

The new protected areas with IUCN V or VI conservation status follow a shared governance model (cf. Gardner et al. 2018) in which the regional government and local communities are accompanied by a legally recognized promoter (e.g., an international NGO, national NGO, universities, or mining companies). To date, out of the 122 protected sites, 43 parks and reserves in the IUCN categories I to IV are managed by Madagascar National Parks (MNP, parastatal organization), i.e., 37% of the total land protected area and some 44% of protected forests as of 2017. The rest of the parks and reserves are mainly under the lead of international NGOs such as WWF International, Conservation International, Wildlife Conservation Society, Missouri Botanical Garden, The Peregrine Fund, Durrell.

The current system of protected areas encompasses a total land area of 6.4 million hectares, of which 3.6 million hectares were forests in 2017. However, compared to 2010 this means a reduction of protected forests by 6% (Table MA1). The IUCN V and VI protected areas show the largest deforestation over this period (-8.3% forest cover, a normal phenomenon given the inclusive aspect of these protected areas (Table MA1). Nevertheless, protected areas do slow down for loss, because forest disappears almost twice as fast outside compared to within the

protected areas (Table MA1). Humid forests are found in the east and north of Madagascar, dry forests along the west side and dry-spiny forests-thickets in the south (Figure MA1, Table MA2).

Table MA1. Forest loss between 2010 and 2017 in protected areas according to their IUCN status, change in their status, and forest loss outside of protected areas. (forest cover in 2010 and 2017 from Vieilledent et al. 2018; NPA = new protected areas designed as early as 2008 but formal protection under IUCN and the decree of classification were postponed until 2015 following the January 2009 coup; I = IUCN category I, … VI = IUCN category VI).

IUCN status	Forest cover (ha)		Forest change 2010–2017
	2010	2017	
NPA (2010) to IUCN I or III (2017)	54 664	51 239	-6.3%
NPA (2010) to IUCN II or IV (2017)	758 462	722 505	-4.7%
NPA (2010) to IUCN V or VI (2017)	1 845 119	1 691 829	-8.3%
IUCN I (2010) to IUCN II (2017)	165 590	158 293	-4.4%
IUCN I or III	43 501	40 668	-6.5%
IUCN II or IV	990 181	959 928	-3.1%
Forests in protected areas	3 857 517	3 624 462	-6.0%
Forests outside protected areas	5 444 722	4 805 407	-11.7%
Total	9 302 239	8 429 869	-9.4%

Table MA2. Forest loss in protected areas according to the main forest types (modified from Moat & Smith (2007)).

Forest type	Forest cover (ha) and % of total protected forests		% loss 2010–2017
	2010	2017	
Humid forest	2 365 168 (61.3%)	2 269 917 (62.6%)	4.0%
Sub-humid forest	40 094 (1.0%)	37 980 (1.0%)	5.3%
Western dry forest	704 934 (18.3%)	637 015 (17.6%)	9.6%
Southwestern dry forest-thicket	691 728 (17.9%)	630 681 (17.4%)	8.8%
Mangrove	55 592 (1.4%)	48 870 (1.3%)	12.1%
Total forest in protected areas	3 857 517	3 624 462	6.0%

Policy implementation tools (question 3)

"Carrot": A scheme widely used and stemming from the end of the NEAP era are REDD+ (Reducing Emissions from Deforestation and Degradation) initiatives (Ferguson 2009). The effectiveness of these approaches to both deliver for improved livelihoods and increased conservation benefits show mixed effects (Brimont et al. 2017, Harvey et al. 2018, MacKinnon et al. 2018, Neudert et al. 2018).

"Stick": Madagascar was the first country in the World to develop and implement a roadmap for conservation and development. During the New Environmental Action Plan (NEAP), 1990–2007, the international donor community funnelled over 700M USD for conservation to Madagascar (Waeber et al. 2016). A reorganization of natural resources management in the country led to a paradigm shift in how to manage the forests aimed at decentralization of forest governance in Madagascar (Mercier 2006, Raik 2007). A new System of Protected Areas of Madagascar (Système des Aires Protégées de Madagascar SAPM) was established in 2002. For this, the protected area legislation Code des Aires Protégées (N. 848-05/N. 2001/05) was legalized in 2003. Revisions of the hunting law (2006-098) and the Convention on International Trade in Endangered Species of Wild Fauna and Flora CITES were undertaken to control the exploitation of wildlife and regulating in-situ conservation (Decree 2006-400) (Rakotoarivelo et al. 2011). The legislation code for protected areas has been revised in 2015 under the Refonte du Code des Aires Protégées (N. 2015-005) (Waeber et al. 2015).

Another threat that made international news in the past decade is the overexploitation of rosewood and ebony species (Randriamalala and Liu 2011, Waeber et al. 2019). Similar to gold rushes, thousands of people entered protected forests (Randriamalala and Liu 2011), causing forest degradation and hunting of wildmeat. Governance responses in terms of monitoring, reporting, policing, and policy changes were ambiguous as highest political echelons had vested interests in the export of the precious woods (Randriamalala and Liu 2010, Wilmé and Waeber 2019, Waeber et al. 2019). During the latest CITES CoP18 meeting Madagascar was eager to redress its reputation, and agreed to stop all international as well as national forest transportation of any kind of wood to get a grip on the illegal trafficking of wood and ebony. A latest sign to follow international standards is the commitment to an uplifting of all rosewood species to CITES Appendix I (Wilmé et al. 2020).

"Sermon": Community-based forest management schemes—known as GELOSE, e.g., Pollini et al. 2014, Neudert et al. 2017—intended as local incentives, have been established. To date, over 450 such community agreements are in place (Horning 2018). However, little is known about its efficacy (Rasolofoson et al. 2017).

Net effect of conservation and intensification (question 4)

Madagascar has ratified the CBD in 1995. To reach the Aichi Biodiversity Targets, the country has substantially increased the number and area of protected forests. Protected areas are the single most effective forest conservation tool in Madagascar while forests outside protection are losing quality and shrinking quickly (Table MA1, Vieilledent et al. 2018). However, globally highest human fertility rates leading to fast growing population in need of increased food and energy security—putting mainly PAs of categories V and VI under increased pressure; vastness and remoteness of the forest frontiers, as well as international dependence over financial and

technical support, and land grabbing, pose the greatest challenges to forest conservation and governance in Madagascar.

References

Brimont, L., Ezzine-de-Blas, D. and Karsenty, A. 2017. The cost of making compensation payments to local forest populations in a REDD+ pilot project in Madagascar. *Madagascar Conservation & Development* 12: 25–33. https://doi.org/10.4314/mcd.v12i1.3

Dudley, N., Parrish, J. D., Redford, K. H. and Stolton, S. 2010. The revised IUCN protected area management categories: the debate and ways forward. Oryx 44: 485–490. https://doi.org/10.1017/S0030605310000566

Ferguson, B. 2009. REDD comes into fashion in Madagascar. *Madagascar Conservation & Development* 4: 132–137. https://doi.org/10.4314/mcd.v4i2.48654

Gardner, C. J., Nicoll, M. E., Birkinshaw, C., Harris, A., Lewis, R. E., et al. 2018. The rapid expansion of Madagascar's protected area system. *Biological Conservation* 220: 29–36. https://doi.org/10.1016/j.biocon.2018.02.011

Hansen, M.C., Potapov, P. V., Moore, R., Hancher, M., Turubanova, S. A., et al. 2013. High-resolution global maps of 21st-century forest cover change. *Science* 342: 850–853. https://doi.org/10.1126/science.1244693

Harvey, C. A., Rambeloson, A. M., Andrianjohaninarivo, T., Andriamaro, L., Rasolohery, A., et al. 2018. Local perceptions of the livelihood and conservation benefits of small-scale livelihood projects in Rural Madagascar. Society & Natural Resources 31: 1045–1063 https://doi.org/10.1080/08941920.2018.1484974

Horning, N. R. 2018. Across the great divide: Collaborative forest management. In: The Politics of Deforestation in Africa: Madagascar, Tanzania, and Uganda. Horning, N. R. (ed.), pp. 135–163. Palgrave Mcmillan: Basingstoke, UK. https://doi.org/10.1007/978-3-319-76828-1_4

MacKinnon, J. L., Andriamaro, L., Rambeloson, A., Razafindrazakasoa, M. and Harvey, C. A. 2018. Costs of delivery approaches for providing livelihood projects to local communities as part of REDD+ programmes: an analysis from Madagascar. *Environmental Conservation* 45: 324–332. https://doi.org/10.1017/S0376892917000571

Mercier, J.-R. 2006. Madagascar moving towards sustainable development. The preparation of the National Environmental Action Plan (NEAP): Was it a false start? *Madagascar Conservation & Development* 1: 50–54. https://doi.org/10.4314/mcd.v1i1.44122

Moat, J. and Smith, P. (eds.) 2007. Atlas of the vegetation of Madagascar. Atlas de la végétation de Madagascar. Kew Publishing, Royal Botanic Gardens, Kew. Available at https://www.researchgate.net/publication/312443439_Atlas_of_the_vegetation_of_madagascar; https://kew.iro.bl.uk/work/22ee6576-f4c2-4fa8-a2b9-843f473bcfaf

Neudert, R., Ganzhorn, J. U. and Waetzold, F. 2017. Global benefits and local costs: The dilemma of tropical forest conservation. A review of the situation in Madagascar. Environmental Conservation 44, 82–96. https://doi.org/10.1017/S0376892916000552

Neudert, R., Olschofsky, K., Kübler, D., Prill, L., Köhl, M. and Wätzold, F. 2018. Opportunity costs of conserving a dry tropical forest under REDD+: The case of the spiny dry forest in southwestern Madagascar. *Forest Policy and Economics* 95: 102–114. https://doi.org/10.1016/j.forpol.2018.07.013

Pollini, J., Hockley, N., Muttenzer, F. D. and Ramamonjisoa, B. S. 2014. The transfer of natural resource management rights to local communities. In: Conservation and Environmental Management in Madagascar. Scales, I. (ed.), p. 172–192. Routledge, London and New York.

Raik, D. 2007. Forest management in Madagascar: An historical overview. Madagascar Conservation & Development 2: 5–10. https://doi.org/10.4314/mcd.v2i1.44123

Rakotoarivelo, A. R., Razafimanahaka, J. H., Rabesihanaka, S., Jones, J. P. and Jenkins, R. K. 2011. Lois et règlements sur la faune sauvage à Madagascar : Progrès accomplis et besoins du futur. *Madagascar Conservation & Development* 6: 37–44. https://doi.org/10.4314/mcd.v6i1.68063

Randriamalala, H. and Liu, Z. 2010. Rosewood of Madagascar: Between democracy and conservation. *Madagascar Conservation & Development* 5: 11–22. https://doi.org/10.4314/mcd.v5i1.57336

Rasolofoson, R. A., Ferraro, P. J., Ruta, G., Rasamoelina, M. S., Randriankolona, P. L., et al. 2017. Impacts of community forest management on human economic well-being across Madagascar. Conservation Letters 10: 346–353. https://doi.org/10.1111/conl.12272

Styger, E., Rakotondramasy, H. M., Pfeffer, M. J., Fernandes, E. C. M. and Bates, D. M. 2007. Influence of slash-and-burn farming practices on fallow succession and land degradation in the rainforest region of Madagascar. *Agriculture, Ecosystems and Environment* 119: 257–269. https://doi.org/10.1016/j.agee.2006.07.012

Vieilledent, G., Grinand, C., Rakotomalala, F. A., Ranaivosoa, R., Rakotoarijaona, J. R., et al. 2018. Combining global tree cover loss data with historical national forest-cover maps to look at six decades of deforestation and forest fragmentation in Madagascar. *Biological Conservation* 222: 189–197. https://doi.org/10.1016/j.biocon.2018.04.008

Waeber, P. O., Wilmé, L., Ramamonjisoa, B., Garcia, C., Rakotomalala, D., et al. 2015. Dry forests in Madagascar, neglected and under pressure. *International Forestry Review* 17: 127–148. https://doi.org/10.1505/146554815815834822

Waeber, P. O., Wilmé, L., Mercier, J.-R., Camara, C. and Lowry II, P. P. 2016. How effective have thirty years of internationally driven conservation and development efforts been in Madagascar? *PLoS ONE* 11: e0161115. https://doi.org/10.1371/journal.pone.0161115

Waeber, P. O., Schuurman, D., Ramamonjisoa, B., Langrand, M., Barber, C.V., et al. 2019. Uplisting of Malagasy precious woods critical for their survival. *Biological Conservation* 235: 89–92. https://doi.org/10.1016/j.biocon.2019.04.007

Waeber, P. O., Rafanoharana, S., Rasamuel, H. A., Wilmé, L. 2020. Parks and Reserves in Madagascar: Managing Biodiversity for a Sustainable Future. In: Protected Areas, National Parks and Sustainable Future. Bakar, A., Suratman, M. N. (eds.). IntechOpen, London UK. https://doi.org/10.5772/intechopen.85348

Wilmé, L. and Waeber, P. O. 2019. Madagascar: Guard last of the forests. *Nature* 565: 567. https://doi.org/10.1038/d41586-019-00323-6

Wilmé, L., Goodman, S. M. and Ganzhorn, J. U. 2006. Biogeographic evolution of Madagascar's microendemic biota. *Science* 312: 1063–1065. https://doi.org/10.1126/science.1122806

Wilmé, L., Ravokatra, M., Dolch, R., Schuurman, D., Mathieu, E., et al. 2012. Toponyms for centers of endemism in Madagascar. *Madagascar Conservation & Development* 7: 30–40. https://doi.org/10.4314/mcd.v7i1.6

Wilmé, L., Innes, J. L., Schuurman, D., Ramamonjisoa, B., Langrand, M., et al. 2020. The elephant in the room: Madagascar's rosewood stocks and stockpiles. *Conservation Letters* e12714. https://doi.org/10.1111/conl.12714

Australia's Mountain Ash and Alpine Ash trees in Victoria (15)
By David Lindenmayer

Case study description
Australia supports a wide range of native forest types and associated biodiversity with different silvicultural systems employed across different ecosystems (Florence 1996). The focus of this case study is on the montane ash forests of the Central Highlands region of Victoria (south-eastern Australia). Montane ash forests are dominated by either Mountain Ash (*Eucalyptus regnans*) or Alpine Ash (*Eucalyptus delegatensis*) trees. These forests were selected as a case study because they are the focus of more than 70% of all timber harvesting in the State of Victoria. The montane ash forests in the Central Highlands of Victoria are not only important for wood production but they also generate most of the water for the >5 million people of Melbourne (Taylor et al. 2019) and provide habitat for an array of forest-dependent species of conservation concern (Taylor and Lindenmayer 2019).

Montane ash forests are a key part of what is termed the Damp Forest Ecological Vegetation Class (EVC) under the ecosystem classification system adopted by the Victorian Government (Taylor and Lindenmayer, 2020). There is approximately 171 000 ha of Mountain Ash and Alpine Ash forest in the Central Highlands of Victoria. The overall spatial extent of montane ash forests in the Central Highlands region have remained little changed in the 220 years since European settlement (Burns et al., 2015), although the condition of the forest has been extensively modified in that time, especially the extent of old growth which has declined from 30-60% to approximately 1.2% of the estate.

However, the condition of the montane ash forest estate is poor and many threatened species are declining very rapidly (e.g. 50-80% population declines in approximately 20 years) (Lindenmayer and Sato 2018). In addition, just 1.16% of the Mountain Ash ecosystem and 0.47% of the Alpine Ash ecosystem is old growth, this is 1/30th-1/60th of what it was before European settlement (Lindenmayer and McCarthy 2002). Montane ash forests supports several dozen threatened forest-dependent species as identified under Victorian government laws (Taylor and Lindenmayer 2019). Indeed, the Mountain Ash ecosystem is classified as Critically Endangered under the IUCN Red Listed Ecosystem approach and is close to ecological collapse as a result of recurrent wildfire and widespread clearcut logging (Lindenmayer and Sato 2018). (Taylor et al. 2017).

Portfolios of conservation instruments and area proportions (question 1)
Currently, 80% of the montane ash forest estate is in wood production areas, and 20% of the forest is formally reserved (Lindenmayer et al. 2015). Outside the reserve system, steep and rocky areas, streamside zones and old growth forest are protected (Lindenmayer et al. 2013). In addition, habitat for threatened species is protected (Blair et al. 2018). Thus, the extent of the formal protected areas exceeds the 17% AICHI reservation target.

Effectiveness (question 2)
"Carrot": Variable retention harvest strategies have been found to be useful in contributing to the on-site persistence of some bird and terrestrial mammal species (Lindenmayer et al. 2019) but nevertheless all kinds of logging have largely negative impacts on threatened species (Taylor and Lindenmayer 2019). For example, trees retained on logged areas have a high risk of collapsing

within a few years of an areas being logged (Lindenmayer et al. 2018), as found in other jurisdictions (e.g. Estonia, Rosenvald et al., 2019). In addition, logging leads to very high levels of forest fragmentation – the mean distance from a retained area in wood production forest to a disturbance edge is 71 m versus 1700 m in reserved forest (Taylor and Lindenmayer, 2020). Logging also occurs in areas of high conservation value as determined through species distribution models for species of conservation concern (Taylor and Lindenmayer 2019). Finally, empirical studies have revealed that logging makes forest that have regenerated after harvesting more prone to subsequent high-severity (crown-burning and crown-scorching) wildfire (Taylor et al. 2014). These effects are relatively long-lived and persist for up to 40 years following logging and stand regeneration (Taylor et al. 2014).

"Stick": Given this array of impacts, some analyses have indicated that almost all of the existing montane ash forest estate needs to be protected with logging removed from Mountain Ash and Alpine Ash ecosystems (Todd et al. 2016; Taylor et al. 2017).

"Sermon": Legacies of forest conflicts in Australia have constrained policy, governance and tenure so that many policy objectives have not been realized (Colloff et al. 2016). Analysing policy implementation gaps for the maintenance of hollow-bearing trees, Treby et al. (2014) observed that at the local council level <5% of had plans for the conservation and management of this important habitat structure.

Policy implementation tools (question 3)

The Victorian Government has several options available to it in dealing with the conservation, problems that have emerged in montane ash forests. First, the State Government agency responsible for managing logging operations has sought to gain Forest Stewardship Council certification its wood, but has failed to do so several times in the past decade, in part because of impact on forest biodiversity and key ecological processes. Key buyers of wood products have indicated that they will no longer buy paper and timber sourced from montane ash forests unless certification has been achieved. Second, environmental and economic accounting (Keith et al. 2017) has shown that the value of water generated from montane ash forests is 25.5. times the value of the timber produced from the same forests. This is important because logging results in a highly significant reduction in water yield (Taylor et al. 2019). The water offset cost through sourcing water from a desalination is large and a significant extra financial burden for taxpayers (Vardon et al. 2019). Notably, widespread past overcutting, combined with recurrent wildfire means that resources available for continued sawlog production are rapidly being exhausted. Indeed, in late 2019, the Victorian Government announced that timber production from all native forests, and not just montane ash forests would cease by 2030. This provides an opportunity to increase levels of protection and expand the old growth estate, which will be an important part of attempts to restore natural fire regimes and limit the risks of frequent reburning of forests (Taylor et al. 2014).

Net effect of conservation and intensification (question 4)

Several analyses have shown that existing reserve systems are not sufficient for supporting viable populations of several key threatened species in montane ash forests and need to be significantly expanded (Todd et al. 2016), particularly in the event of further wildfires. The case study from the Central Highlands of Victoria has some important wider general implications for the global forest environment frontier. First, it shows that even reservation levels somewhat

higher than those specified in the Aichi targets may be insufficient to adequacy conserve some key elements of biodiversity and maintain critical ecological processes at their natural levels (e.g. fire regimes). Second, the case study shows that a failure to account for the effects of natural disturbances such as wildfire on resource availability can result in overcutting and the eventual collapse of an industry (Lindenmayer 2017). Third, the condition of a forest resource is fundamentally important. In the case of montane ash forests, the overall extent of these ecosystems has remained largely unchanged over the past 220 years, but the extent of loss of old growth forest (which provide key long-term habitat for the majority of bird species and many arboreal marsupials including those of conservation concern) has meant there is a high risk of collapse of forest biodiversity (Lindenmayer and Sato 2018). Finally, ongoing logging operations, even reduced intensity practices such as Variable Retention Harvesting, have a negative impact on forest biodiversity and ecological processes such as through contributing to added fire risks (Taylor et al. 2014) and further fragmenting already highly modified environments (Taylor and Lindenmayer 2020). The only way to resolve these issues, including attempting to restore natural fire regimes, is to instigate greater protection of the forest and begin to recover large areas of intact old growth forest.

References

Blair, D., McBurney, L. and Lindenmayer, D. B. 2018. Failing to conserve Leadbeater's Possum and its Mountain Ash forest habitat. *Australian Zoologist* 39:443-448.

Colloff, M. J., Doherty, M. D., Lavorel, S., Dunlop, M., Wise, R. M. and Prober, S. M. 2016. Adaptation services and pathways for the management of temperate montane forests under transformational climate change. *Climatic Change* 138(1-2):267-282.

Florence, R. G. 1996. Ecology and Silviculture of Eucalypt Forests. CSIRO Publishing, Melbourne.

Keith, H., Vardon, M., Stein, J.A.R., Stein, J.L. and Lindenmayer, D. B. 2017. Ecosystem accounts define explicit and spatial trade-offs for managing natural resources. *Nature Ecology and Evolution* 1:1683-1692.

Lindenmayer, D. 2017. Halting natural resource depletion: Engaging with economic and political power. *The Economic and Labour Relations Review* 28:41-56.

Lindenmayer, D. B., Blair, D. and McBurney, L. 2019. Variable retention harvesting in Victoria's Mountain Ash (Eucalyptus regnans) forests (southeastern Australia). *Ecological Processes* 8.

Lindenmayer, D.B., Blair, D., McBurney, L., Banks, S. C., Stein, J. A. R., Hobbs, R. J., Likens, G. E. and J. F. Franklin. 2013. Principles and practices for biodiversity conservation and restoration forestry: a 30 year case study on the Victorian montane ash forests and the critically endangered Leadbeater's Possum. *Australian Zoologist* 36:441-460.

Lindenmayer, D. B., Blair, D. P., McBurney, L. and Banks, S.C. 2015. Mountain Ash: Fire, Logging and the Future of Victoria's Giant Forests. CSIRO Publishing, Melbourne.

Lindenmayer, D. B., Blanchard, W., Blair D. and McBurney, L. 2018. The road to oblivion – quantifying pathways in the decline of large old trees. *Forest Ecology and Management* 430:259-264.

Lindenmayer, D. B. and McCarthy, M.A. 2002. Congruence between natural and human forest disturbance: a case study from Australian montane ash forests. *Forest Ecology and Management* 155:319-335.

Lindenmayer, D. B. and Sato, C. 2018. Hidden collapse is driven by fire and logging in a socioecological forest ecosystem. *Proceedings of the National Academy of Sciences* 115:5181-5186.

Taylor, C., Blair, D., Keith, H. and Lindenmayer, D.B. 2019. Modelling water yields in response to logging and Representative Climate Futures. *Science of the Total Environment* 688:890-902.

Taylor, C., Cadenhead, N., Lindenmayer, D.B. and Wintle B.A. 2017. Improving the design of a conservation reserve for a critically endangered species. *PLOS One* 12:e0169629.

Taylor, C. and D. B. Lindenmayer. 2019. The adequacy of Victoria's protected areas to conserve its forest-dependent fauna? *Austral Ecology* 44:1076-1090.

Taylor, C. and Lindenmayer, D. B. 2020. Temporal fragmentation of a critically endangered forest ecosystem. *Austral Ecology* 45(3):340-354.

Taylor, C., M. McCarthy, A. and Lindenmayer, D. B.. 2014. Non-linear effects of stand age on fire severity. *Conservation Letters* 7:355-370.

Todd, C. R., Lindenmayer, D. B., Stamation, K., Acevedo-Catteneo, S., Smih, S. and Lumsden, L.F. 2016. Assessing reserve effectiveness: Application to a threatened species in a dynamic fire prone forest landscape. *Ecological Modelling* 338:90-100.

Treby, D. L., Castley, J. G., & Hero, J. M. 2014. Forest conservation policy implementation gaps: consequences for the management of hollow-bearing trees in Australia. *Conservation and Society* 12(1):16-26.

Vardon, M., May, S., Keith, H. and Lindenmayer, D. B. 2019. Accounting and valuing the ecosystem services related to water supply in the Central Highlands of Victoria, Australia. *Ecosystem Services* 39:101004.

New Zealand (16)
By Dejan Firm and Juan Monge

Case study description
New Zealand's forest resources can be divided into two distinct, and almost mutually exclusive, forest ecosystems (MfE & Stats NZ 2018, MPI-NEFD 2019): planted and indigenous forests accounting for a total forested area of 8.71 10^6 hectares (33% of total land area). Exotic production forests are dominated by radiata/Monterey pine (*Pinus radiata*) planted in 90% of the plantation area. Indigenous forests have four major physiognomic elements: southern beeches (*Fuscospora spp.* and *Lophozonia sp.*), broadleaved angiosperm trees, kauri (*Agathis australis*), and other conifers (predominantly podocarps) (Wardle 1991). The indigenous forest cover is mainly preserved in hilly and mountainous areas, i.e. less accessible and non-arable areas; most notably along the western side of the South Island. Only small fragments of indigenous forests remain in lowland and coastal environments (MfE & Stats NZ 2018). Having a large planted forest resource (1.75 10^6 ha or 20%) enables New Zealand to manage its Crown (public ownership) and privately-owned indigenous forests primarily for conservation, as they are a key part of the nation's environment and heritage, and harbour indigenous biodiversity.

Portfolios of conservation instruments and area proportions (question 1)
Indigenous forests account for approximately 80% (6.96 10^6 ha) of the total forest area. The Crown is the major owner of tall indigenous forests and manages 77% of them through the Department of Conservation (DOC) for biodiversity conservation, environmental benefits, tourism, and recreation. Furthermore, regional and local authorities administer significant tracts of protected areas and are responsible for the sustainable management of these areas through different acts (e.g. Resource Management Act (RMA) 1991, Reserves Act 1977).

Other formally protected forests are privately-, Māori- or community-owned and managed natural areas, which are usually established through government and/or privately funded covenant schemes (e.g. Ngā Whenua Rahui, Nature Heritage Fund, Queen Elizabeth II (QEII) National Trust). Forests protected under the QEII mechanism (81 10^3 ha) are an example of private land covenants, which allows individuals to keep ownership of a piece of land and protect portions of it in perpetuity regardless of future land ownership change. Another example of conservation land covenants, specifically designed for Māori owners, are forests protected under the Ngā Whenua Rāhui Fund scheme (124 10^3 ha); these 25-year reviewable covenants are for land with important ecological and cultural significance to Māori (i.e. indigenous people of New Zealand).

Production forestry in privately-owned indigenous forests has a very limited extent, as there are no real incentives for this activity, and harvest levels have been in a steady decline since 1990s (Allen et al. 2013). Currently, timber production could be exercised by using ecologically sustainable forest management principles (e.g. continuous-cover forestry) on less than 5% of privately-owned indigenous forests that are not part of the protected areas network, i.e. ≈50 10^3 hectares.

Biodiversity conservation within intensively managed exotic forests is usually not a priority, although, in certain landscapes they do provide habitat for some indigenous species such as the endemic New Zealand falcon (*Falco novaeseelandiae*) and bats (Pawson et al. 2010, Seaton et

al. 2009, Seaton et al. 2010). Some plantation owners started incorporating different measures to mitigate the negative impacts of harvesting on the New Zealand falcon, as this is deemed beneficial for sustainable management certification purposes, e.g. Forest Stewardship Council.

Table NZ1. Basic information about 3 groups of indigenous forest conservation instruments in New Zealand.

	Public conservation forests and local council reserves	Voluntary – private and Māori-owned conservation forests	Private sustainably managed forests
Area (ha) and proportion of all forest land in 2019 (6.96 10^6 ha)	5.4 10^6 (77%)	205 10^3 (3%)	50 10^3 (0.7%)
Aim	Conserve and manage indigenous biodiversity on public land	Conserve and manage indigenous biodiversity on private land	Consideration of biodiversity conservation in privately owned forests managed for timber production
Establishment	1977, 1980, 1987	1977, 1990	1993
Target size	Variable	Variable	Variable
Duration	Permanent	Permanent or variable	Variable (10 or 50 years)
Decision by	Central or local government	Voluntary set-aside by the land owner	Central government
Control	Government agencies (DOC), local and regional governance bodies	Government agencies (DOC), QEII Trust, Ngā Whenua Rāhui	Government agencies (MPI-Te Uru Rakau, DOC)
Monitoring	National spatial databases	National spatial databases, covenanting authorities	Spatial databases, field inventory, forest management plans and permits

Table NZ2. Proportion of all indigenous forest conservation instruments in New Zealand, and their effectiveness.

	Conservation instruments (% of all indigenous forest land)				
	Formal protection (i.e. public conservation forests)	Voluntary protection (i.e. private and Māori land covenants, regional council conservation forests)	Unproductive forest	Tree retention (indigenous forests managed by CCF principles)	SUM
New Zealand	77	3	NA	<1	81
South Island	89	<1	NA	<1	90
South Island – Western ecoregion	93	<1	NA	NA	93
North Island	58	6	NA	<1	65
North Island – Northern ecoregion	45	3	NA	NA	48

Effectiveness (question 2)

New Zealand has the third highest proportion of national land area 'under environmental protection' across OECD countries (OECD 2017). The area of public conservation land (includes other ecosystems, not just forests) has increased from approximately 7.4 10^6 hectares in 1990 to 8.5 10^6 hectares in 2016 (32% of New Zealand's total land area) (MfE & Stats NZ 2018). However, the extent of protected areas varies quite strongly between different regions, e.g. 44% of the South Island and 15% of the North Island.

In regard to forests, there has been a net loss of native forests of about 0.2% between 1996 and 2012, equivalent to 16 10^3 hectares (MfE & Stats NZ 2018). Moreover, New Zealand's protected areas network does not cover a fully representative range of forest ecosystem types – it includes a higher proportion of alpine ecosystems and montane indigenous forest and ecosystems such as lowland forest or coastal forest are under-represented (DOC & MfE 2000, Cieraad et al. 2015).

Policy implementation tools (question 3)

Carrot: Besides owning conservation land, DOC also administers incentive mechanisms for conservation projects on private land by providing financial assistance for private landowners and covenanting schemes such as the Ngā Whenua Rahui Fund. The funding provided by covenanting schemes usually helps cover the administrative costs of legal protection, costs of fencing, pest and weed management. For post-1989 (early successional) forests owners can claim carbon credits (incentive to not clear) within the New Zealand Emission Trading Scheme (Carver

& Kerr 2017), which is currently the only well-established market mechanism. There is also a variety of nationwide and community-based initiatives that promote and partially fund native tree plantings and restoration activities for enhancing biodiversity. These are organized and enabled mainly through publicly funded programmes (e.g. One Billion Trees Programme) or public-private partnerships (e.g. Trees That Count).

Stick: Administrative mechanisms include different conservation classifications established by DOC based on three main legal acts (i.e. National Parks Act 1980, Conservation Act 1987, Reserves Act 1977). The level of protection stretches from the more lenient category of stewardship land in conservation areas (where activities are permitted) to the more stringent category of wilderness areas in national parks (where no infrastructure is permitted). There are also different levels of management that are a combination of the different conservation categories and threat levels to protected ecosystem and species. The management levels range from intensive ecosystem management to smaller scale action. Forests that are designated as above described conservation areas (i.e. land subsequently reserved by local or central government) or are part of one of the covenanting schemes can be regarded as formally legally protected.

The level of protection for forest vegetation on private land is provided by clearance and management rules in district plans (e.g. effects of land use on biodiversity) based on the provisions of the RMA, but these protection measures are much less rigorously enforced. District and regional councils have rules constraining the clearance of native vegetation, albeit the criteria, implementation and the actual completeness of spatial databases vary widely between councils.

Sermon: Besides DOC there are several well-known non-governmental organizations that advocate for the conservation of forest biodiversity in the landscapes and communicate the importance of it to the public. They also encourage and provide advice to interested landowners and communities about forest restoration and biodiversity management related issues and activities, e.g. tree planting, pest and weed management. Currently, practicing of ecologically sustainable forest management in indigenous forests is highly controversial, even on private land, and this activity has not been particularly promoted since 1990s.

Net effect of conservation and intensification (question 4)

New Zealand society and governing bodies achieved a tremendous conservation goal in the period between 1970s and late 1990s by completely stopping exploitative logging activities in native forests and protecting more than ¾ of the remnant area, including all the old-growth areas of different forest types (Swarbrick 2007, King et al. 2015). This was realized by adopting the land sparing strategy.

One of the open questions in New Zealand remains how to maintain the existing second-growth indigenous forest (approximately 1.4 10^6 ha) and shrubland cover on private and Māori land that is not adequately protected, without impeding the opportunities for sustainable economic development of rural communities. For example, the proportion of Māori land covered with indigenous forest and shrubland is much higher than any other land, apart from public conservation land (MfE & Stats NZ 2018). Development of future conservation strategies for these forests will require a careful consideration of the whole social-ecological context,

especially how might the decisions on protecting and managing biodiversity impact the use and development of Māori land. Through New Zealand's history a range of hurdles impeding the full and optimal use of Māori land for economic development have arisen. Moreover, indigenous forests represent a central role in their culture and values, which determine how they interact with and utilize the natural environment (e.g. a strong emphasis on inter-generational equity and sustainability). Therefore, deploying a set of stringent protection measures like measures in public conservation forests and without providing for activities could unfairly impact on Māori, and worsen disadvantages created by historic confiscation and loss of land.

Another pressing issue is how to increase the indigenous forest cover in certain landscapes, as forest cover extent is highly variable between different regions and landscapes. While, the overall protected forest area percent is exceptional, there are landscapes with very little or zero indigenous forest habitat remaining, and areas where the cover of early- to mid-successional vegetation (e.g. shrublands) is extensive, but not formally legally protected (Cieraad et al. 2015). Defining and implementing a strategy for halting further loss and improving indigenous forest habitat conditions in these landscapes will be very challenging, as this is a wicked problem involving economic, socio-cultural, and political barriers. For example, currently there are no large enough financial compensation schemes that could provide an incentive for landowners to retire the land, i.e. stop reverting to a pasture or establishing an exotic plantation forest. Hence, it is likely that the existing tension between the pressure to clear shrublands for productive land use versus allowing them to revert to forest with consequent carbon storage and biodiversity benefits cannot be controlled solely by different legal provisions (e.g. RMA 1991). Monks et al. (2019) showed that, while the vegetation clearance was constrained by formal legal protection, the positive effect of protection was mainly evident for areas and vegetation that private owners have no intention of clearing anyway. Hence, the success of different conservation initiatives on private land remains piecemeal.

References

Allen R.B., Bellingham, P.J., Holdaway, R.J. and Wiser, S.K. 2013. New Zealand's indigenous forests and shrublands. In: Dymond J.R. ed. Ecosystem services in New Zealand – conditions and trends. Manaaki Whenua Press, Lincoln, New Zealand: pp. 34–48.

Carver, T. and Kerr, S. 2017. Facilitating Carbon Offsets from Native Forests. Motu Working Paper 17-01 (April 2017), Motu Economic and Public Policy Research (Wellington, New Zealand): 32 pp.

Cieraad, E., Walker, S., Price, R. and Barringer, J. 2015. An updated assessment of indigenous cover remaining and legal protection in New Zealand's land environments. *New Zealand Journal of Ecology* 39(2):309–315.

DOC (Department of Conservation), MfE (Ministry for the Environment). 2000. The New Zealand biodiversity strategy: February 2000. Wellington, Department of Conservation and the Ministry for the Environment: 144 pp.

King, C.M., Gaukrodger, D.J. and Ritchie, N.A. (Eds.). 2015. The Drama of Conservation: The History of Pureora Forest, New Zealand. Springer & New Zealand Department of Conservation, Cham: 357 pp.

MfE (Ministry for the Environment), Stats NZ. 2018. New Zealand's Environmental Reporting Series: Our land 2018. Ministry for the Environment and Stats NZ. Retrieved from www.mfe.govt.nz and www.stats.govt.nz.

Monks, A., Hayman, E. and Walker, S. 2019. Attrition of recommended areas for protection: clearance of ecologically significant vegetation on private land. *New Zealand Journal of Ecology* 43(2):1–11.
MPI-NEFD. 2019. National Exotic Forest Description – Report. Ministry for Primary Industries, New Zealand, Edition 36 (December 2019): 63 pp.
OECD. 2017. OECD Environmental Performance Reviews: New Zealand 2017 – Highlights. OECD Publishing, Paris. Retrieved from www.oecd.org/env/country-reviews/Highlights_OECD_EPR_NewZealand.pdf.
Pawson, S.M., Ecroyd, C.E., Seaton, R., Shaw, W.B. and Brockerhoff, E.G. 2010. New Zealand's exotic plantation forests as habitats for threatened indigenous species. *New Zealand Journal of Ecology* 34(3):342–355.
Seaton, R., Holland, J.D., Minot, E.O. and Springett, B.P. 2009. Breeding success of New Zealand falcons (*Falco novaeseelandiae*) in a pine plantation. New *Zealand Journal of Ecology* 33(1):32–39.
Seaton, R., Minot, E.O. and Holland, J.D. 2010. Variation in bird species abundance in a commercial pine plantation in New Zealand. *New Zealand Journal of Forestry* 54(4): 3–11.
Swarbrick, N. 2007. Logging native forests (published 24 September 2007). Te Ara - The Encyclopedia of New Zealand: http://www.TeAra.govt.nz/en/logging-native-forests (accessed 9 April 2020).
Wardle, P. 1991. Vegetation of New Zealand. Cambridge University Press, Cambridge, UK: 672 pp.
